# Supplementary figures and images for: Granick revisited: Synthesizing evolutionary and ecological evidence for the late origin of bacteriochlorophyll via ghost lineages and horizontal gene transfer
Source: PLoS One. 2021 Jan 28;16(1):e0239248. doi: 10.1371/journal.pone.0239248 (PMC7842958; doi:10.1371/journal.pone.0239248)

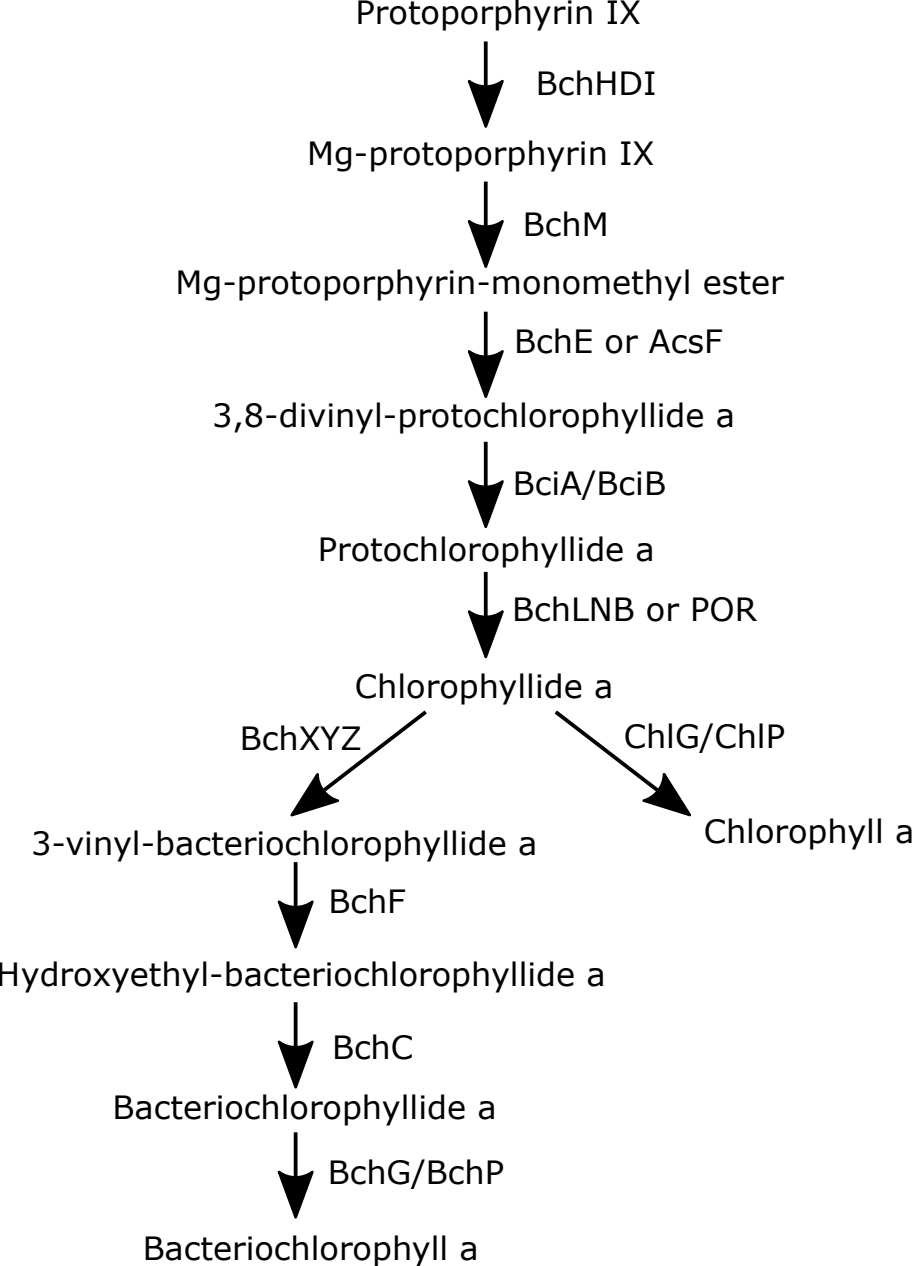

Supplement: S1 Fig — Steps leading to chlorophyllide a synthesis are shared by all photosynthetic bacteria. From chlorophyllide a, a single enzymatic step can produce chlorophyll a (right branch) as performed in Cyanobacteria, or multiple steps can be taken to produce bacteriochlorophyll a (left branch) as performed in most characterized anoxygenic phototrophs. While some steps can be performed by multiple enzymes and some enzymes may act on multiple substrates, allowing some steps to be performed in different orders in different organisms or in parallel in a single organism, the (b)chl biosynthesis pathway is depicted here as a simplified linear pathway for the sake of clarity. Not shown are branch points leading to more evolutionarily derived alternative pigments including bchl c, bchl g, chl b, and chl d. (PDF) [file pone.0239248.s001.pdf]

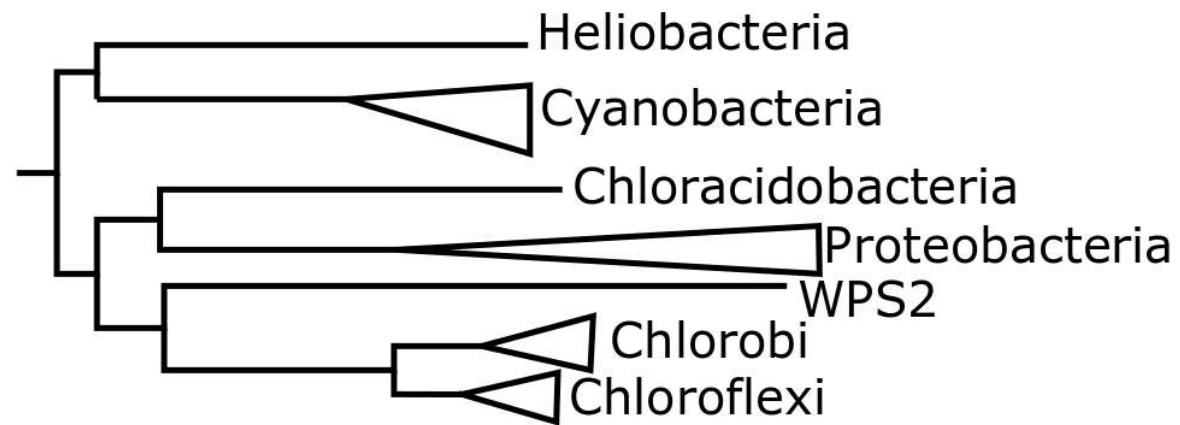

Supplement: S2 Fig — A) Consensus phylogenies of steps in (bacterio)chlorophyll synthesis. Major radiations are collapsed at the phylum level. The Chloroflexi clade includes phototrophic Chloroflexia as well as multiple lineages of phototrophic Anaerolineae. The Proteobacteria clade includes Gemmatimonadetes and in some cases (BchL/N/B) the Synechococcus/Prochlorococcus clade of Cyanobacteria. Consensus tree for earliest dedicated steps in (bacterio)chlorophyll synthesis, including BchH, BchI, and BchD for conversion of protoporphyrin IX to Mg-protoporphyrin IX, and BchM for further conversion to Mg-protoporphyrin monomethyl ester. Consensus tree is rooted based on consistent topology between most branches in all trees, with robust root derived from CobN as an outgroup to BchH. Branch lengths are approximate, derived from the BchM phylogeny. B) Consensus phylogenies of steps in (bacterio)chlorophyll synthesis. Major radiations are collapsed at the phylum level. The Chloroflexi clade includes phototrophic Chloroflexia as well as multiple lineages of phototrophic Anaerolineae. The Proteobacteria clade includes Gemmatimonadetes and in some cases (BchL/N/B) the Synechococcus/Prochlorococcus clade of Cyanobacteria. Consensus tree for BchL, BchN, and BchB, subunits of the DPOR complex for conversion of protochlorophyllide a to chlorophyllide a, the last step shared in chlorophyll and bacteriochlorophyll synthesis pathways. Long branches between BchL/N/B and closest outgroups (BchX/Y/Z) resulted in poorly supported root placement, so tree is presented unrooted. The topology of the BchL/N/B tree is identical at the phylum level to the BchH/D/I/M tree, providing support for interpretation of a shared history of the entire “backbone” (bacterio)chlorophyll synthesis pathway and an inferred root for the BchL/N/B tree on the branch between the Cyanobacteria+Heliobacteria clade and the other phyla. Branch lengths are approximate, derived from the BchL phylogeny. C) Consensus phylogenies of steps in ( [file pone.0239248.s002.pdf]

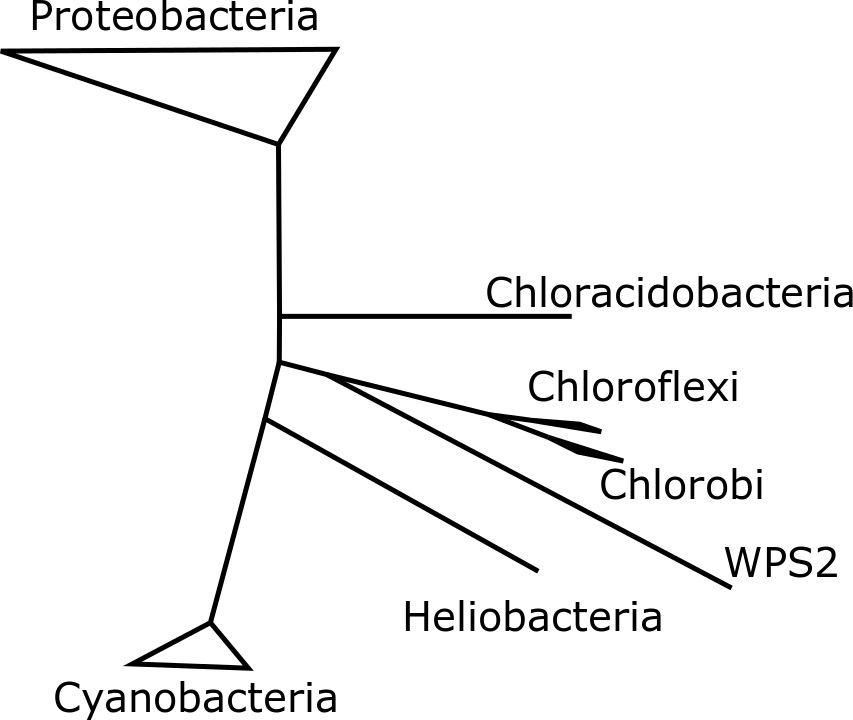

Supplement: S3 Fig — (JPG) [file pone.0239248.s003.jpg]

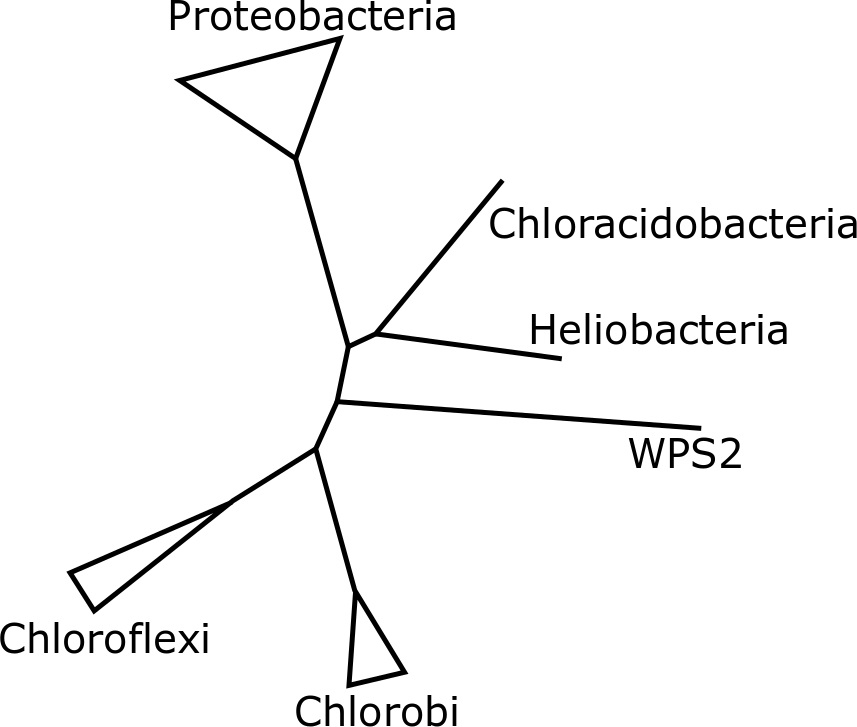

Supplement: S4 Fig — (JPG) [file pone.0239248.s004.jpg]

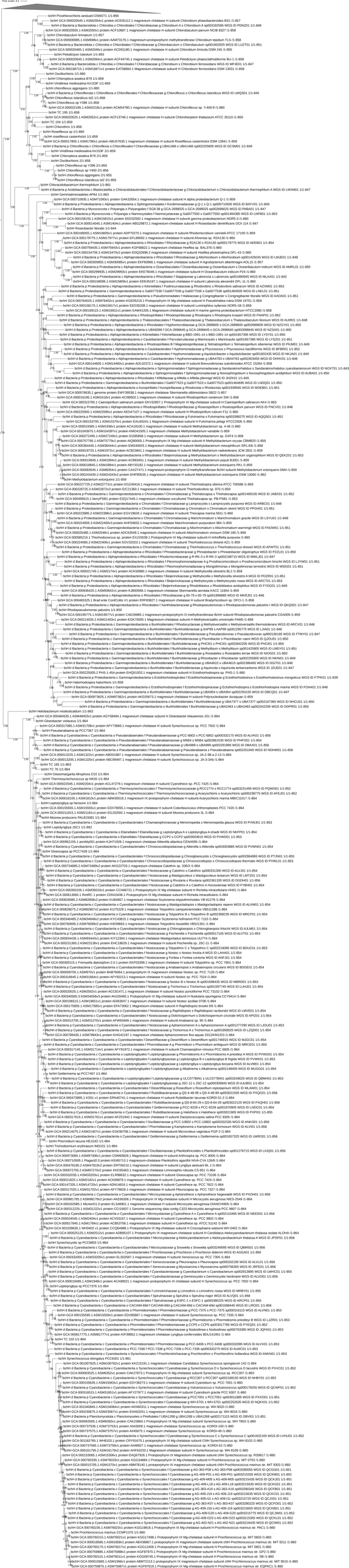

Supplement: S5 Fig — (PDF) [file pone.0239248.s005.pdf]

Tree scale: 1

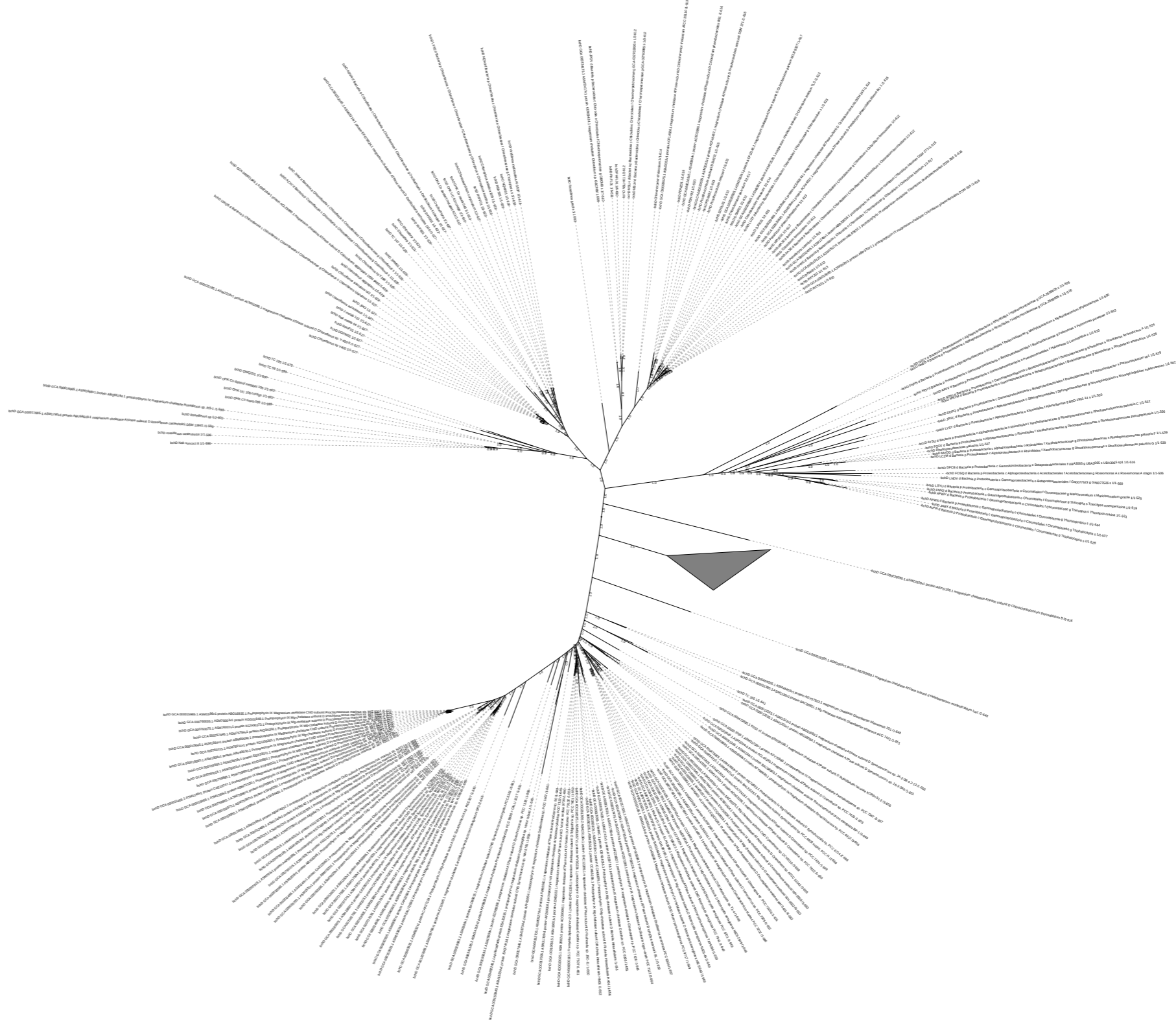

Supplement: S6 Fig — (PDF) [file pone.0239248.s006.pdf]

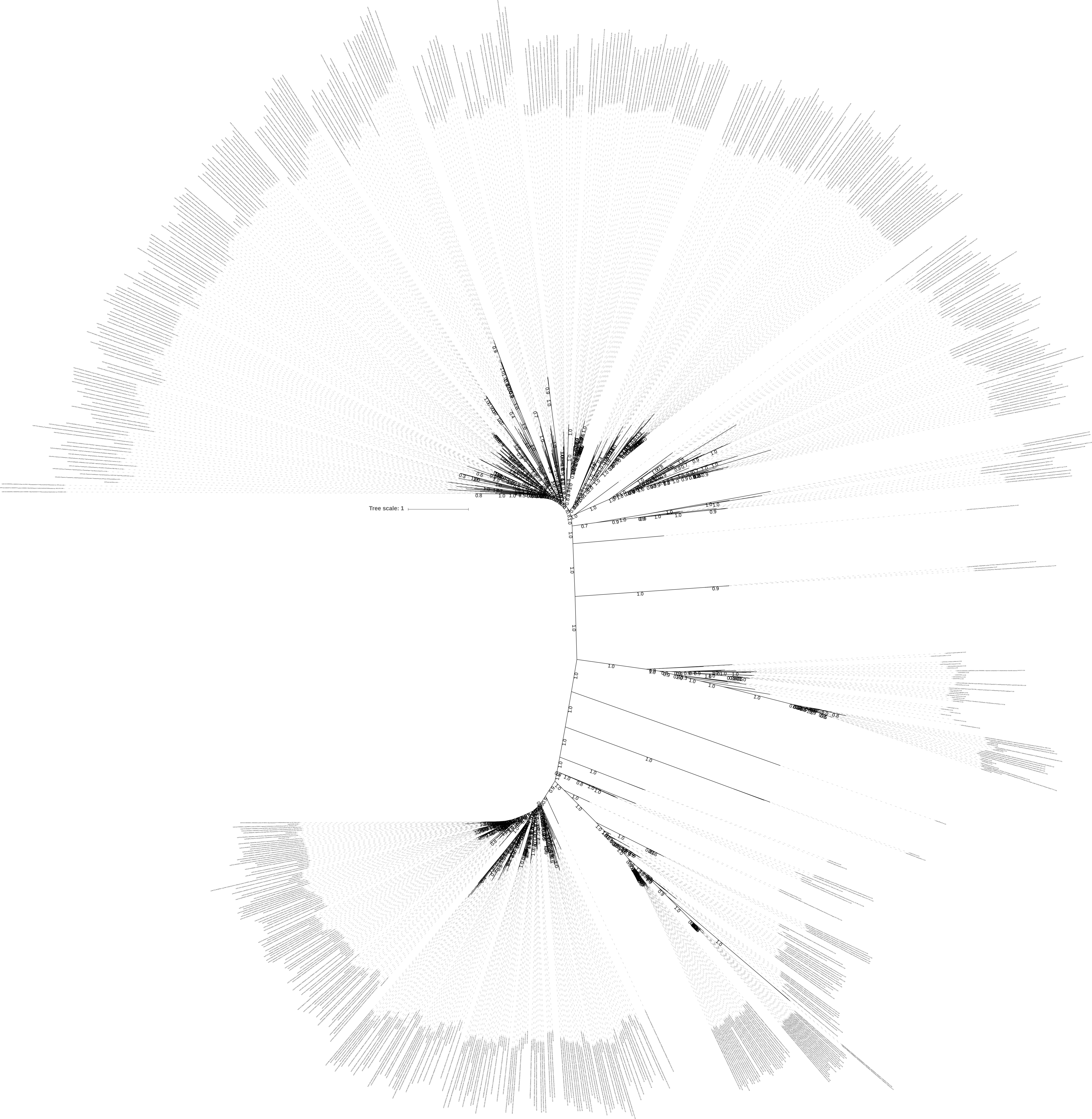

Supplement: S7 Fig — (PDF) [file pone.0239248.s007.pdf]

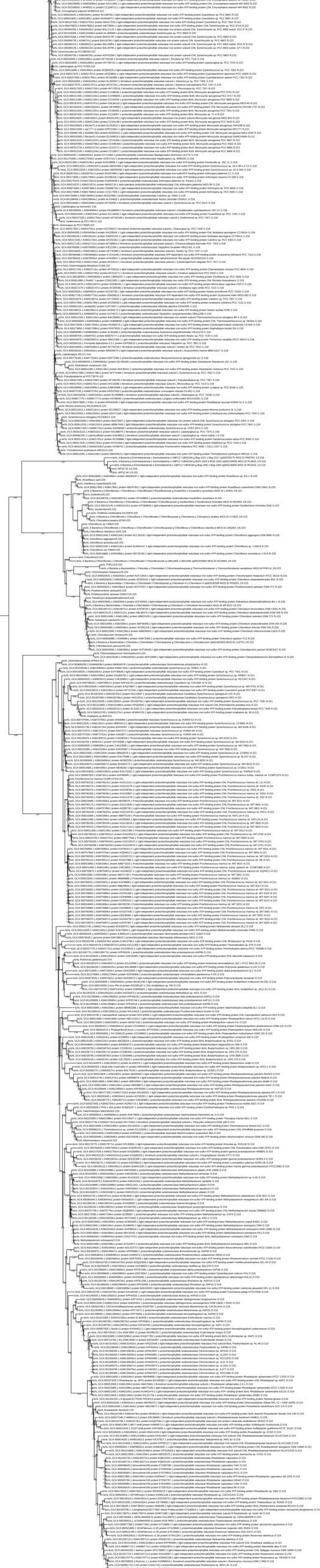

Supplement: S8 Fig — (PDF) [file pone.0239248.s008.pdf]

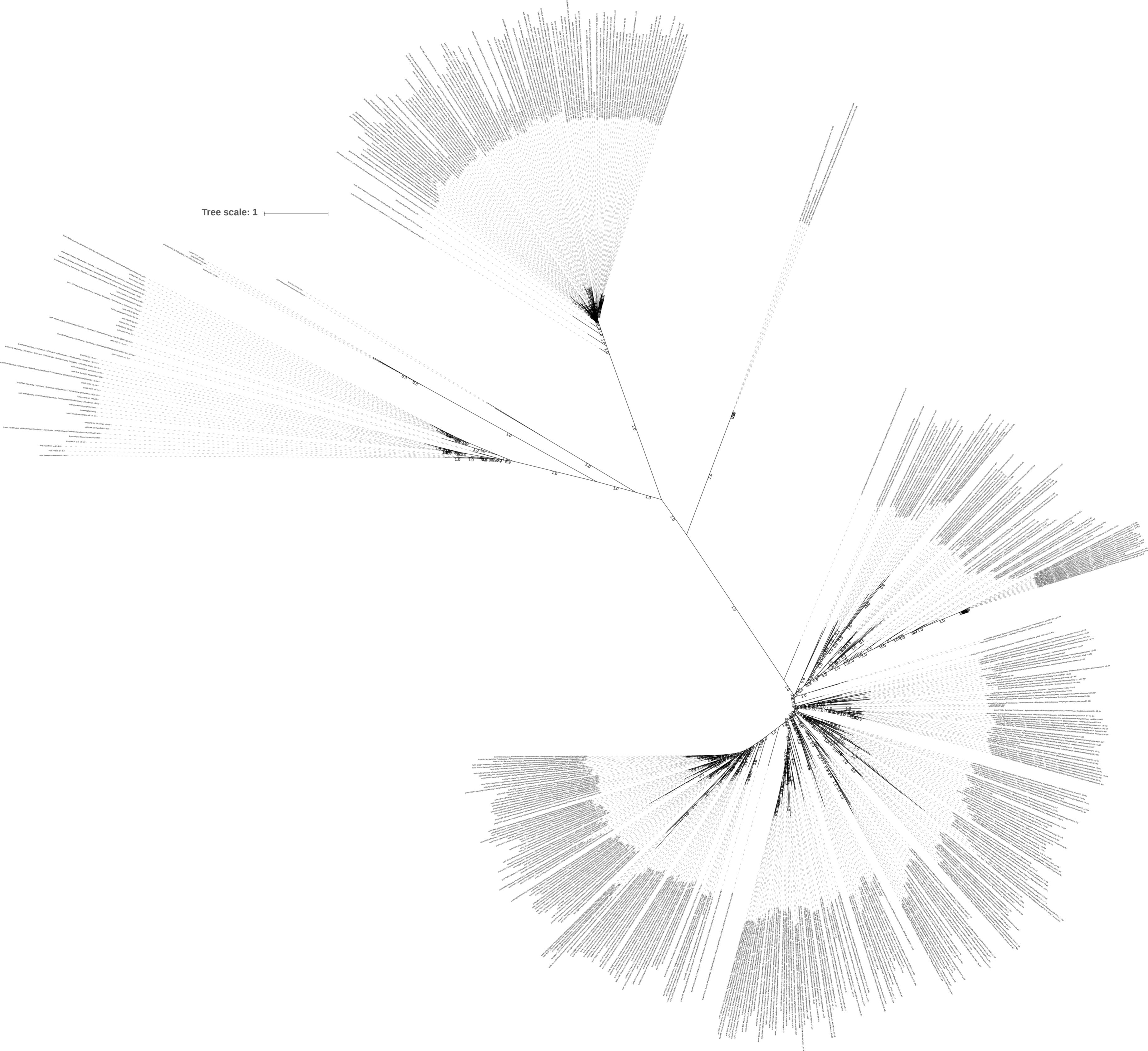

Supplement: S9 Fig — (PDF) [file pone.0239248.s009.pdf]

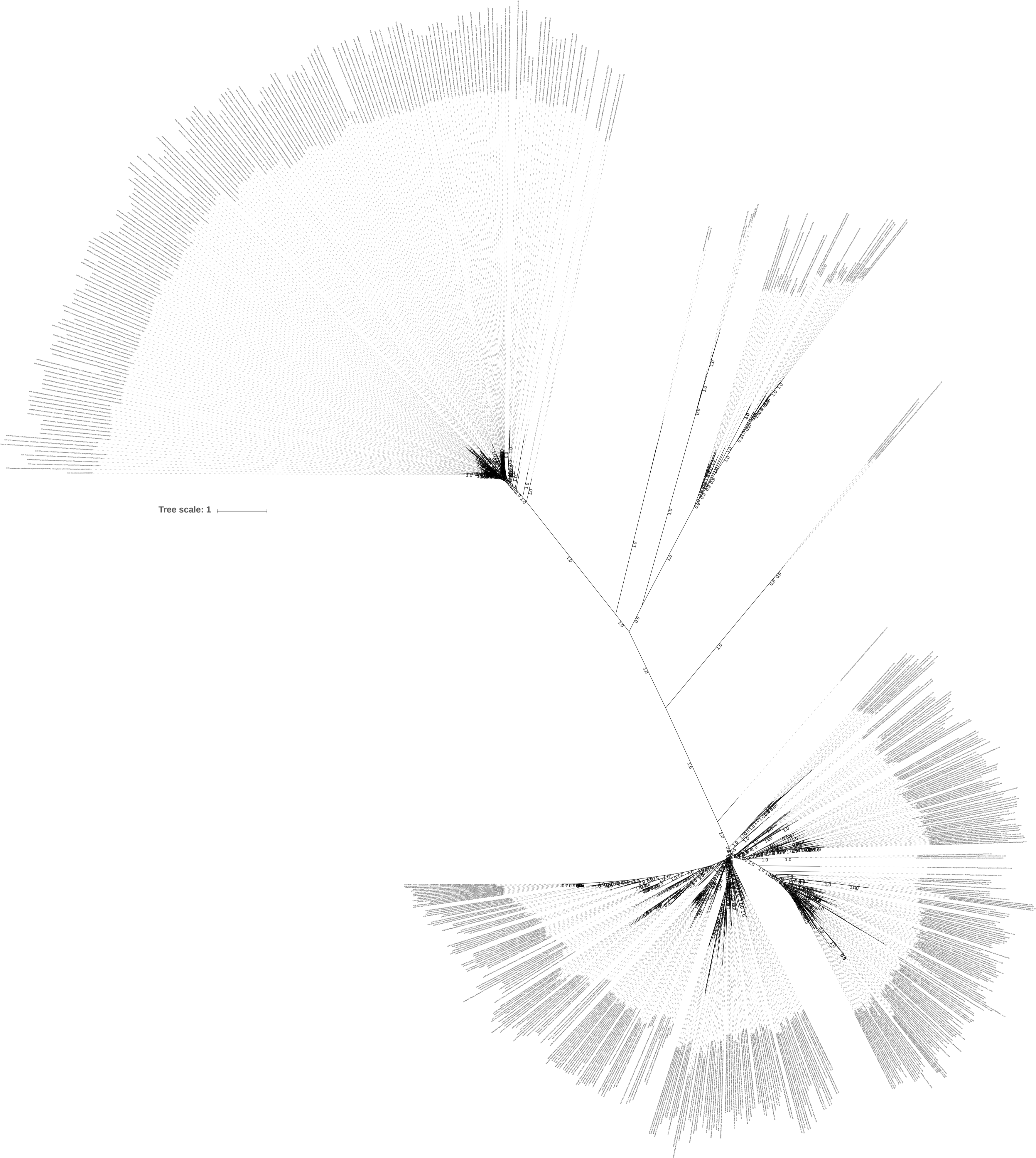

Supplement: S10 Fig — (PDF) [file pone.0239248.s010.pdf]

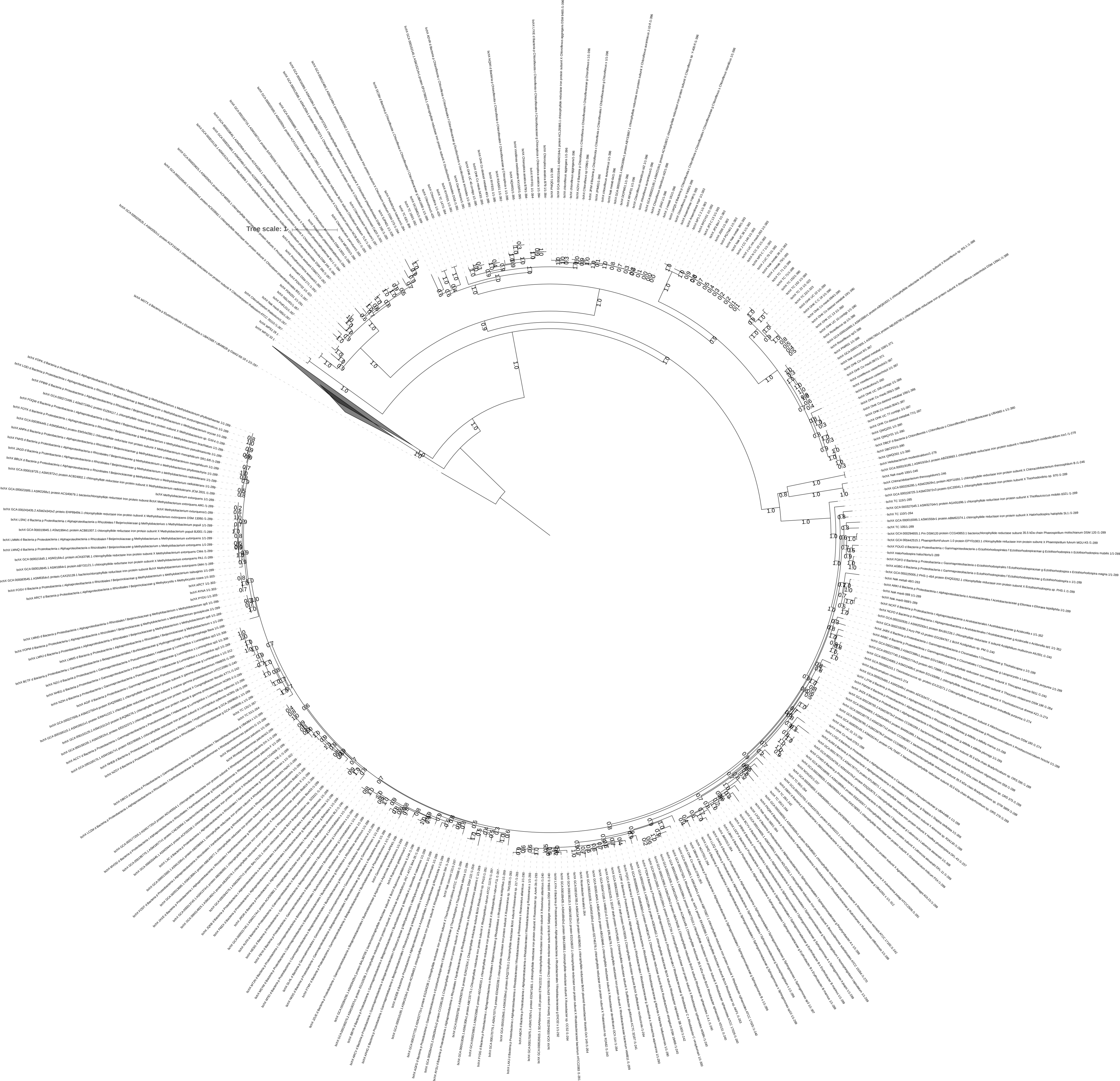

Supplement: S11 Fig — (PDF) [file pone.0239248.s011.pdf]

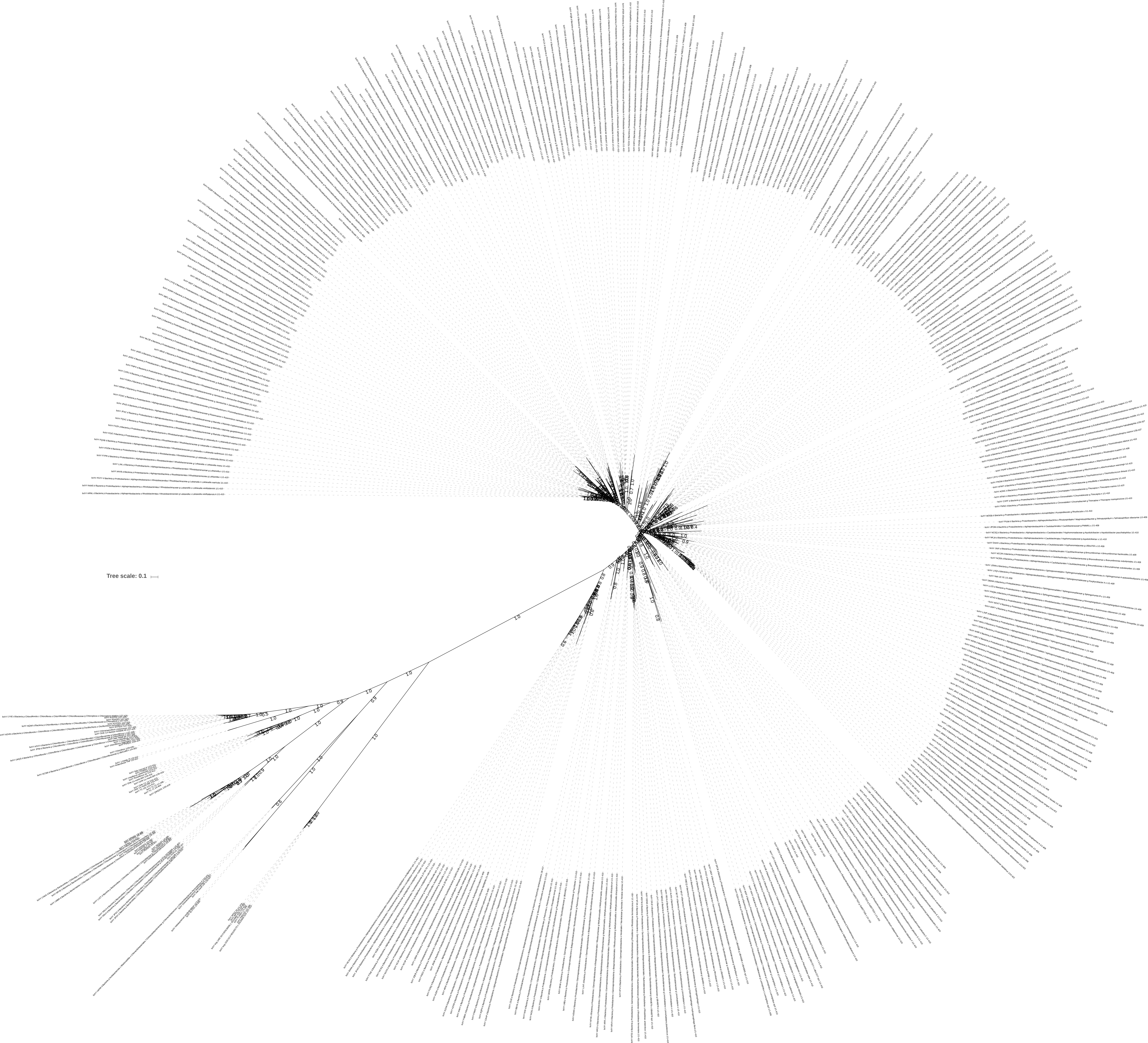

Supplement: S12 Fig — (PDF) [file pone.0239248.s012.pdf]

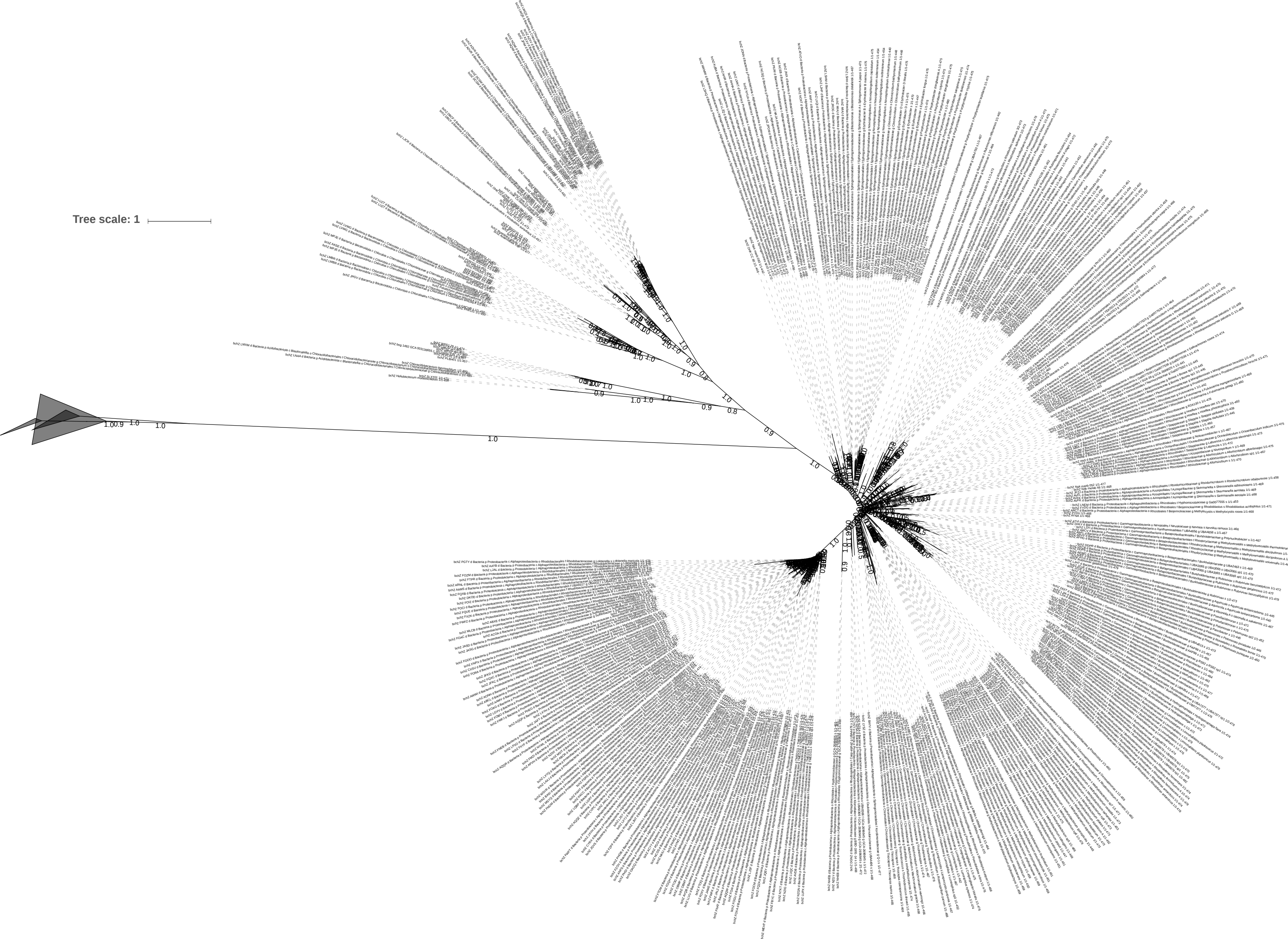

Supplement: S13 Fig — (PDF) [file pone.0239248.s013.pdf]
