# Supplementary material for: Granick revisited: Synthesizing evolutionary and ecological evidence for the late origin of bacteriochlorophyll via ghost lineages and horizontal gene transfer
Source: PLoS One. 2021 Jan 28;16(1):e0239248. doi: 10.1371/journal.pone.0239248 (PMC7842958; doi:10.1371/journal.pone.0239248)
Supplement: S1 Table — (DOCX) [file pone.0239248.s018.docx]

| Gene | Substrate | Product | Presence | Evolutionary relatedness | Phylogeny figure |
| --- | --- | --- | --- | --- | --- |
| bchH | Protoporphyrin IX | Mg-Protoporphyrin IX | All phototrophs (3 copies in Chlorobi and Chloroflexi) |  | Supplemental Figure 3 |
| bchD | Protoporphyrin IX | Mg-Protoporphyrin IX | All phototrophs |  | Supplemental Figure 4 |
| bchI | Protoporphyrin IX | Mg-Protoporphyrin IX | All phototrophs |  | Supplemental Figure 5 |
| bchM | Mg-Protoporphyrin IX | Mg-protoporphyrinmonomethyl ester | All phototrophs except Anaerolineae |  | Supplemental Figure 6 |
| bchL | Protochlorophyllide a | Chlorophyllide a | All phototrophs except Anaerolineae | Homologous to nifH, bchX | Supplemental Figure 7 |
| bchN | Protochlorophyllide a | Chlorophyllide a | All phototrophs except Anaerolineae | Homologous to nifD bchB, bchY, bchZ | Supplemental Figure 8 |
| bchB | Protochlorophyllide a | Chlorophyllide a | All phototrophs except Anaerolineae | Homologous to nifD bchN, bchY, bchZ | Supplemental Figure 9 |
| bchX | Chlorophyllide a | 3-vinyl-bacteriochlorophyllide a | All anoxygenic phototrophs | Homologous to nifH, bchL | Supplemental Figure 10 |
| bchY | Chlorophyllide a | 3-vinyl-bacteriochlorophyllide a | All anoxygenic phototrophs | Homologous to nifD bchB, bchN, bchZ | Supplemental Figure 11 |
| bchZ | Chlorophyllide a | 3-vinyl-bacteriochlorophyllide a | All anoxygenic phototrophs | Homologous to nifD bchB, bchY, bchN | Supplemental Figure 12 |

**S1 Table:** Proteins involved in (bacterio)-chlorophyll synthesis from protoporphyrin IX.
