# Supplementary material for: Granick revisited: Synthesizing evolutionary and ecological evidence for the late origin of bacteriochlorophyll via ghost lineages and horizontal gene transfer
Source: PLoS One. 2021 Jan 28;16(1):e0239248. doi: 10.1371/journal.pone.0239248 (PMC7842958; doi:10.1371/journal.pone.0239248)
Supplement: S14 Fig — (PDF) [file pone.0239248.s014.pdf]

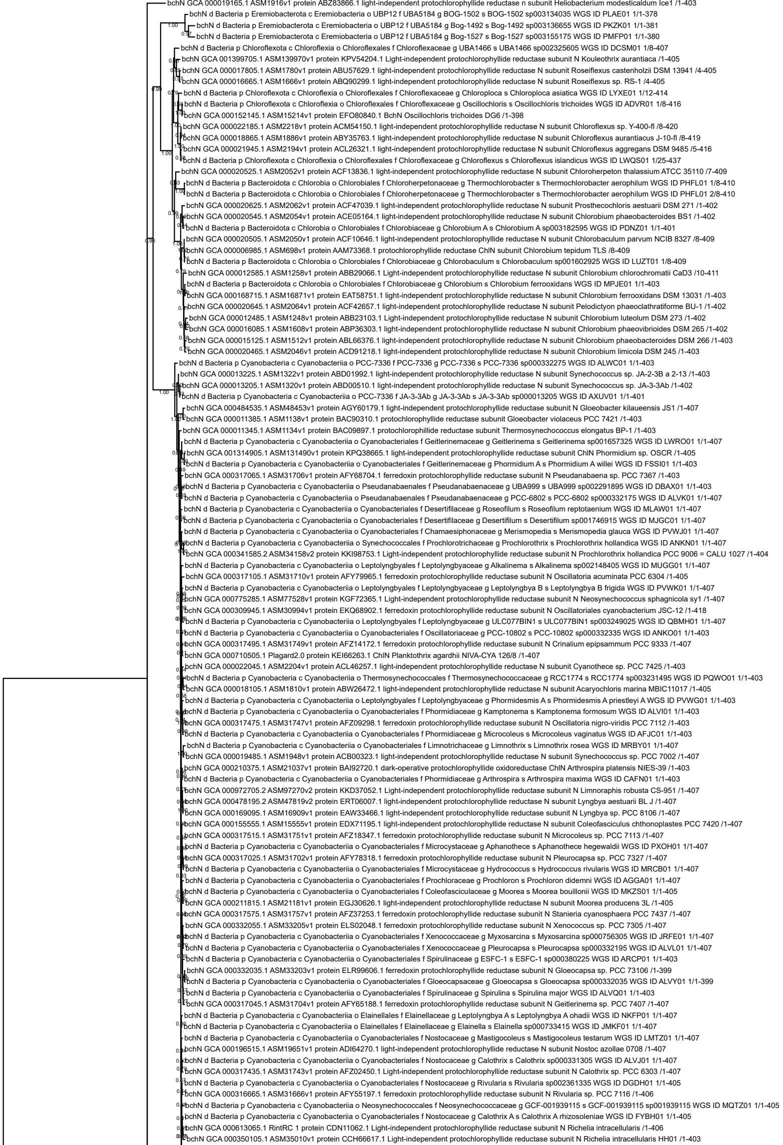
bchN GCA 000019165.1 ASM1916v1 protein ABZ83866.1 light-independent protochlorophyllide reductase n subunit Heliobacterium modesticaldum Ice1 /1-403
bchN d Bacteria p Eremiobacterota c Eremiobacteria o UBP12 f UBA5184 g BOG-1502 s BOG-1502 sp003134035 WGS ID PLAE01 1/1-378
bchN d Bacteria p Eremiobacterota c Eremiobacteria o UBP12 f UBA5184 g Bog-1492 s Bog-1492 sp003136655 WGS ID PKZK01 1/1-381
bchN d Bacteria p Eremiobacterota c Eremiobacteria o UBP12 f UBA5184 g Bog-1527 s Bog-1527 sp003155175 WGS ID PMFP01 1/1-380
bchN d Bacteria p Chloroflexota c Chloroflexia o Chloroflexales f Chloroflexaceae g UBA1466 s UBA1466 sp002325605 WGS ID DCSM01 1/8-407
bchN GCA 001399705.1 ASM139970v1 protein KPV54204.1 Light-independent protochlorophyllide reductase subunit N Kouleothrix aurantiaca /1-405
bchN GCA 000017805.1 ASM1780v1 protein ABU57629.1 light-independent protochlorophyllide reductase N subunit Roseiflexus castenholzii DSM 13941 /4-405
bchN GCA 000016665.1 ASM1666v1 protein ABQ90299.1 light-independent protochlorophyllide reductase N subunit Roseiflexus sp. RS-1 /4-405
bchN d Bacteria p Chloroflexota c Chloroflexia o Chloroflexales f Chloroflexaceae g Chloroploca s Chloroploca asiatica WGS ID LYXE01 1/12-414
bchN d Bacteria p Chloroflexota c Chloroflexia o Chloroflexales f Chloroflexaceae g Oscillochloris s Oscillochloris trichoides WGS ID ADVR01 1/8-416
bchN GCA 000152145.1 ASM15214v1 protein EFO80840.1 BchN Oscillochloris trichoides DG6 /1-398
bchN GCA 000022185.1 ASM2218v1 protein ACM54150.1 light-independent protochlorophyllide reductase N subunit Chloroflexus sp. Y-400-fl /8-420
bchN GCA 000018865.1 ASM1886v1 protein ABY35763.1 light-independent protochlorophyllide reductase N subunit Chloroflexus aurantiacus J-10-fl /8-419
bchN GCA 000021945.1 ASM2194v1 protein ACL26321.1 light-independent protochlorophyllide reductase N subunit Chloroflexus aggregans DSM 9485 /5-416
bchN d Bacteria p Chloroflexota c Chloroflexia o Chloroflexales f Chloroflexaceae g Chloroflexus s Chloroflexus islandicus WGS ID LWQS01 1/25-437
bchN GCA 000020525.1 ASM2052v1 protein ACF13836.1 light-independent protochlorophyllide reductase N subunit Chloroherpeton thalassium ATCC 35110 /7-409
bchN d Bacteria p Bacteroidota c Chlorobia o Chlorobiales f Chloroherpetonaceae g Thermochlorobacter s Thermochlorobacter aerophilum WGS ID PHFL01 1/8-410
bchN d Bacteria p Bacteroidota c Chlorobia o Chlorobiales f Chloroherpetonaceae g Thermochlorobacter s Thermochlorobacter aerophilum WGS ID PHFL01 2/8-410
bchN GCA 000020625.1 ASM2062v1 protein ACF47039.1 light-independent protochlorophyllide reductase N subunit Prosthecochloris aestuarii DSM 271 /1-402
bchN GCA 000020545.1 ASM2054v1 protein ACE05164.1 light-independent protochlorophyllide reductase N subunit Chlorobium phaeobacteroides BS1 /1-402
bchN d Bacteria p Bacteroidota c Chlorobia o Chlorobiales f Chlorobiaceae g Chlorobium A s Chlorobium A sp003182595 WGS ID PDNZ01 1/1-401
bchN GCA 000020505.1 ASM2050v1 protein ACF10646.1 light-independent protochlorophyllide reductase N subunit Chlorobaculum parvum NCIB 8327 /8-409
bchN GCA 000006985.1 ASM698v1 protein AAM73368.1 protochlorophyllide reductase ChlN subunit Chlorobium tepidum TLS /8-409
bchN d Bacteria p Bacteroidota c Chlorobia o Chlorobiales f Chlorobiaceae g Chlorobaculum s Chlorobaculum sp001602925 WGS ID LUZT01 1/8-409
bchN GCA 000012585.1 ASM1258v1 protein ABB29066.1 Light-independent protochlorophyllide reductase N subunit Chlorobium chlorochromatii CaD3 /10-411
bchN d Bacteria p Bacteroidota c Chlorobia o Chlorobiales f Chlorobiaceae g Chlorobium s Chlorobium ferrooxidans WGS ID MPJE01 1/1-403
bchN GCA 000168715.1 ASM16871v1 protein EAT58751.1 light-independent protochlorophyllide reductase N subunit Chlorobium ferrooxidans DSM 13031 /1-403
bchN GCA 000020645.1 ASM2064v1 protein ACF42657.1 light-independent protochlorophyllide reductase N subunit Pelodictyon phaeoclathratiforme BU-1 /1-402
bchN GCA 000012485.1 ASM1248v1 protein ABB23103.1 Light-independent protochlorophyllide reductase N subunit Chlorobium luteolum DSM 273 /1-402
bchN GCA 000016085.1 ASM1608v1 protein ABP36303.1 light-independent protochlorophyllide reductase N subunit Chlorobium phaeovibrioides DSM 265 /1-402
bchN GCA 000015125.1 ASM1512v1 protein ABL66376.1 light-independent protochlorophyllide reductase N subunit Chlorobium phaeobacteroides DSM 266 /1-403
bchN GCA 000020465.1 ASM2046v1 protein ACD91218.1 light-independent protochlorophyllide reductase N subunit Chlorobium limicola DSM 245 /1-403
bchN d Bacteria p Cyanobacteria c Cyanobacteriia o PCC-7336 f PCC-7336 g PCC-7336 s PCC-7336 sp000332275 WGS ID ALWC01 1/1-403
bchN GCA 000013225.1 ASM1322v1 protein ABD01992.1 light-independent protochlorophyllide reductase N subunit Synechococcus sp. JA-2-3B a 2-13 /1-403
bchN GCA 000013205.1 ASM1320v1 protein ABD00510.1 light-independent protochlorophyllide reductase N subunit Synechococcus sp. JA-3-3Ab /1-402
bchN d Bacteria p Cyanobacteria c Cyanobacteriia o PCC-7336 f JA-3-3Ab g JA-3-3Ab s JA-3-3Ab sp000013205 WGS ID AXUV01 1/1-401
bchN GCA 000484535.1 ASM48453v1 protein AGY60179.1 light-independent protochlorophyllide reductase subunit N Gloeobacter kilaueensis JS1 /1-407
bchN GCA 000011385.1 ASM1138v1 protein BAC90310.1 protochlorophyllide reductase subunit Gloeobacter violaceus PCC 7421 /1-403
bchN GCA 000011345.1 ASM1134v1 protein BAC09897.1 protochlorophillide reductase subunit Thermosynechococcus elongatus BP-1 /1-403
bchN d Bacteria p Cyanobacteria c Cyanobacteriia o Cyanobacteriales f Geitlerinemaceae g Geitlerinema s Geitlerinema sp001657325 WGS ID LWRO01 1/1-407
bchN GCA 001314905.1 ASM131490v1 protein KPQ38665.1 light-independent protochlorophyllide reductase subunit ChlN Phormidium sp. OSCR /1-405
bchN d Bacteria p Cyanobacteria c Cyanobacteriia o Cyanobacteriales f Geitlerinemaceae g Phormidium A s Phormidium A willei WGS ID FSSI01 1/1-403
bchN GCA 000317065.1 ASM31706v1 protein AFY68704.1 ferredoxin protochlorophyllide reductase subunit N Pseudanabaena sp. PCC 7367 /1-403
bchN d Bacteria p Cyanobacteria c Cyanobacteriia o Pseudanabaenales f Pseudanabaenaceae g UBA999 s UBA999 sp002291895 WGS ID DBAX01 1/1-403
bchN d Bacteria p Cyanobacteria c Cyanobacteriia o Pseudanabaenales f Pseudanabaenaceae g PCC-6802 s PCC-6802 sp000332175 WGS ID ALVK01 1/1-407
bchN d Bacteria p Cyanobacteria c Cyanobacteriia o Cyanobacteriales f Desertifilaceae g Roseofilum s Roseofilum reptotaenium WGS ID MLAW01 1/1-407
bchN d Bacteria p Cyanobacteria c Cyanobacteriia o Cyanobacteriales f Desertifilaceae g Desertifilum s Desertifilum sp001746915 WGS ID MJGC01 1/1-407
bchN d Bacteria p Cyanobacteria c Cyanobacteriia o Cyanobacteriales f Chamaesiphonaceae g Merismopedia s Merismopedia glauca WGS ID PVWJ01 1/1-407
bchN d Bacteria p Cyanobacteria c Cyanobacteriia o Synechococcales f Prochlorotrichaceae g Prochlorothrix s Prochlorothrix hollandica WGS ID ANKN01 1/1-407
bchN GCA 000341585.2 ASM34158v2 protein KKI98753.1 Light-independent protochlorophyllide reductase subunit N Prochlorothrix hollandica PCC 9006 = CALU 1027 /1-404
bchN d Bacteria p Cyanobacteria c Cyanobacteriia o Leptolyngbyales f Leptolyngbyaceae g Alkalinema s Alkalinema sp002148405 WGS ID MUGG01 1/1-407
bchN GCA 000317105.1 ASM31710v1 protein AFY79965.1 ferredoxin protochlorophyllide reductase subunit N Oscillatoria acuminata PCC 6304 /1-405
bchN d Bacteria p Cyanobacteria c Cyanobacteriia o Leptolyngbyales f Leptolyngbyaceae g Leptolyngbya B s Leptolyngbya B frigida WGS ID PVWK01 1/1-407
bchN GCA 000775285.1 ASM77528v1 protein KGF72365.1 Light-independent protochlorophyllide reductase subunit N Neosynechococcus sphagnicola sy1 /1-407
bchN GCA 000309945.1 ASM30994v1 protein EKQ68902.1 ferredoxin protochlorophyllide reductase subunit N Oscillatoriales cyanobacterium JSC-12 /1-418
bchN d Bacteria p Cyanobacteria c Cyanobacteriia o Leptolyngbyales f Leptolyngbyaceae g ULC077BIN1 s ULC077BIN1 sp003249025 WGS ID QBMH01 1/1-407
bchN d Bacteria p Cyanobacteria c Cyanobacteriia o Cyanobacteriales f Oscillatoriaceae g PCC-10802 s PCC-10802 sp000332335 WGS ID ANKO01 1/1-403
bchN GCA 000317495.1 ASM31749v1 protein AFZ14172.1 ferredoxin protochlorophyllide reductase subunit N Crinalium epipsammum PCC 9333 /1-407
bchN GCA 000710505.1 Plagard2.0 protein KEI66263.1 ChlN Planktothrix agardhii NIVA-CYA 126/8 /1-407
bchN GCA 000022045.1 ASM2204v1 protein ACL46257.1 light-independent protochlorophyllide reductase N subunit Cyanothece sp. PCC 7425 /1-403
bchN d Bacteria p Cyanobacteria c Cyanobacteriia o Thermosynechococcales f Thermosynechococcaceae g RCC1774 s RCC1774 sp003231495 WGS ID PQWO01 1/1-403
bchN GCA 000018105.1 ASM1810v1 protein ABW26472.1 light-independent protochlorophyllide reductase N subunit Acaryochloris marina MBIC11017 /1-405
bchN d Bacteria p Cyanobacteria c Cyanobacteriia o Leptolyngbyales f Leptolyngbyaceae g Phormidesmis A s Phormidesmis A priestleyi A WGS ID PVWG01 1/1-403
bchN d Bacteria p Cyanobacteria c Cyanobacteriia o Cyanobacteriales f Phormidiaceae g Kamptonema s Kamptonema formosum WGS ID ALVI01 1/1-403
bchN GCA 000317475.1 ASM31747v1 protein AFZ09298.1 ferredoxin protochlorophyllide reductase subunit N Oscillatoria nigro-viridis PCC 7112 /1-403
bchN d Bacteria p Cyanobacteria c Cyanobacteriia o Cyanobacteriales f Phormidiaceae g Microcoleus s Microcoleus vaginatus WGS ID AFJC01 1/1-403
bchN d Bacteria p Cyanobacteria c Cyanobacteriia o Cyanobacteriales f Limnotrichaceae g Limnothrix s Limnothrix rosea WGS ID MRBY01 1/1-407
bchN GCA 000019485.1 ASM1948v1 protein ACB00323.1 light-independent protochlorophyllide reductase N subunit Synechococcus sp. PCC 7002 /1-407
bchN GCA 000210375.1 ASM21037v1 protein BAI92720.1 dark-operative protochlorophyllide oxidoreductase ChlN Arthrospira platensis NIES-39 /1-403
bchN d Bacteria p Cyanobacteria c Cyanobacteriia o Cyanobacteriales f Phormidiaceae g Arthrospira s Arthrospira maxima WGS ID CAFN01 1/1-403
bchN GCA 000972705.2 ASM97270v2 protein KKD37052.1 Light-independent protochlorophyllide reductase subunit N Limnoraphis robusta CS-951 /1-407
bchN GCA 000478195.2 ASM47819v2 protein ERT06007.1 light-independent protochlorophyllide reductase N subunit Lyngbya aestuarii BL J /1-407
bchN GCA 000169095.1 ASM16909v1 protein EAW33466.1 light-independent protochlorophyllide reductase subunit N Lyngbya sp. PCC 8106 /1-407
bchN GCA 000155555.1 ASM15555v1 protein EDX71195.1 light-independent protochlorophyllide reductase N subunit Coleofasciculus chthonoplastes PCC 7420 /1-407
bchN GCA 000317515.1 ASM31751v1 protein AFZ18347.1 ferredoxin protochlorophyllide reductase subunit N Microcoleus sp. PCC 7113 /1-407
bchN d Bacteria p Cyanobacteria c Cyanobacteriia o Cyanobacteriales f Microcystaceae g Aphanothece s Aphanothece hegewaldii WGS ID PXOH01 1/1-407
bchN GCA 000317025.1 ASM31702v1 protein AFY78318.1 ferredoxin protochlorophyllide reductase subunit N Pleurocapsa sp. PCC 7327 /1-407
bchN d Bacteria p Cyanobacteria c Cyanobacteriia o Cyanobacteriales f Microcystaceae g Hydrococcus s Hydrococcus rivularis WGS ID MRCB01 1/1-407
bchN d Bacteria p Cyanobacteria c Cyanobacteriia o Cyanobacteriales f Prochloraceae g Prochloron s Prochloron didemni WGS ID AGGA01 1/1-407
bchN d Bacteria p Cyanobacteria c Cyanobacteriia o Cyanobacteriales f Coleofasciculaceae g Moorea s Moorea bouillonii WGS ID MKZS01 1/1-405
bchN GCA 000211815.1 ASM21181v1 protein EGJ30626.1 light-independent protochlorophyllide reductase N subunit Moorea producens 3L /1-405
bchN GCA 000317575.1 ASM31757v1 protein AFZ37253.1 ferredoxin protochlorophyllide reductase subunit N Stanieria cyanosphaera PCC 7437 /1-407
bchN GCA 000332055.1 ASM33205v1 protein ELS02048.1 ferredoxin protochlorophyllide reductase subunit N Xenococcus sp. PCC 7305 /1-407
bchN d Bacteria p Cyanobacteria c Cyanobacteriia o Cyanobacteriales f Xenococcaceae g Myxosarcina s Myxosarcina sp000756305 WGS ID JRFE01 1/1-407
bchN d Bacteria p Cyanobacteria c Cyanobacteriia o Cyanobacteriales f Xenococcaceae g Pleurocapsa s Pleurocapsa sp000332195 WGS ID ALVL01 1/1-407
bchN d Bacteria p Cyanobacteria c Cyanobacteriia o Cyanobacteriales f Spirulinaceae g ESFC-1 s ESFC-1 sp000380225 WGS ID ARCP01 1/1-403
bchN GCA 000332035.1 ASM33203v1 protein ELR99606.1 ferredoxin protochlorophyllide reductase subunit N Gloeocapsa sp. PCC 73106 /1-399
bchN d Bacteria p Cyanobacteria c Cyanobacteriia o Cyanobacteriales f Gloeocapsaceae g Gloeocapsa s Gloeocapsa sp000332035 WGS ID ALVY01 1/1-399
bchN d Bacteria p Cyanobacteria c Cyanobacteriia o Cyanobacteriales f Spirulinaceae g Spirulina s Spirulina major WGS ID ALVQ01 1/1-403
bchN GCA 000317045.1 ASM31704v1 protein AFY65188.1 ferredoxin protochlorophyllide reductase subunit N Geitlerinema sp. PCC 7407 /1-407
bchN d Bacteria p Cyanobacteria c Cyanobacteriia o Elainellales f Elainellaceae g Leptolyngbya A s Leptolyngbya A ohadii WGS ID NKFP01 1/1-407
bchN d Bacteria p Cyanobacteria c Cyanobacteriia o Elainellales f Elainellaceae g Elainella s Elainella sp000733415 WGS ID JMKF01 1/1-407
bchN d Bacteria p Cyanobacteria c Cyanobacteriia o Cyanobacteriales f Nostocaceae g Mastigocoleus s Mastigocoleus testarum WGS ID LMTZ01 1/1-407
bchN GCA 000196515.1 ASM19651v1 protein ADI64270.1 light-independent protochlorophyllide reductase N subunit Nostoc azollae 0708 /1-407
bchN d Bacteria p Cyanobacteria c Cyanobacteriia o Cyanobacteriales f Nostocaceae g Calothrix s Calothrix sp000331305 WGS ID ALVJ01 1/1-407
bchN GCA 000317435.1 ASM31743v1 protein AFZ02450.1 Light-independent protochlorophyllide reductase subunit N Calothrix sp. PCC 6303 /1-407
bchN d Bacteria p Cyanobacteria c Cyanobacteriia o Cyanobacteriales f Nostocaceae g Rivularia s Rivularia sp002361335 WGS ID DGDH01 1/1-405
bchN GCA 000316665.1 ASM31666v1 protein AFY55197.1 ferredoxin protochlorophyllide reductase subunit N Rivularia sp. PCC 7116 /1-406
bchN d Bacteria p Cyanobacteria c Cyanobacteriia o Neosynechococcales f Neosynechococcaceae g GCF-001939115 s GCF-001939115 sp001939115 WGS ID MQTZ01 1/1-405
bchN d Bacteria p Cyanobacteria c Cyanobacteriia o Cyanobacteriales f Nostocaceae g Calothrix A s Calothrix A rhizosoleniae WGS ID FYBH01 1/1-405
bchN GCA 000613065.1 RintRC 1 protein CDN11062.1 Light-independent protochlorophyllide reductase subunit N Richelia intracellularis /1-406
bchN GCA 000350105.1 ASM35010v1 protein CCH66617.1 Light-independent protochlorophyllide reductase subunit N Richelia intracellularis HH01 /1-403

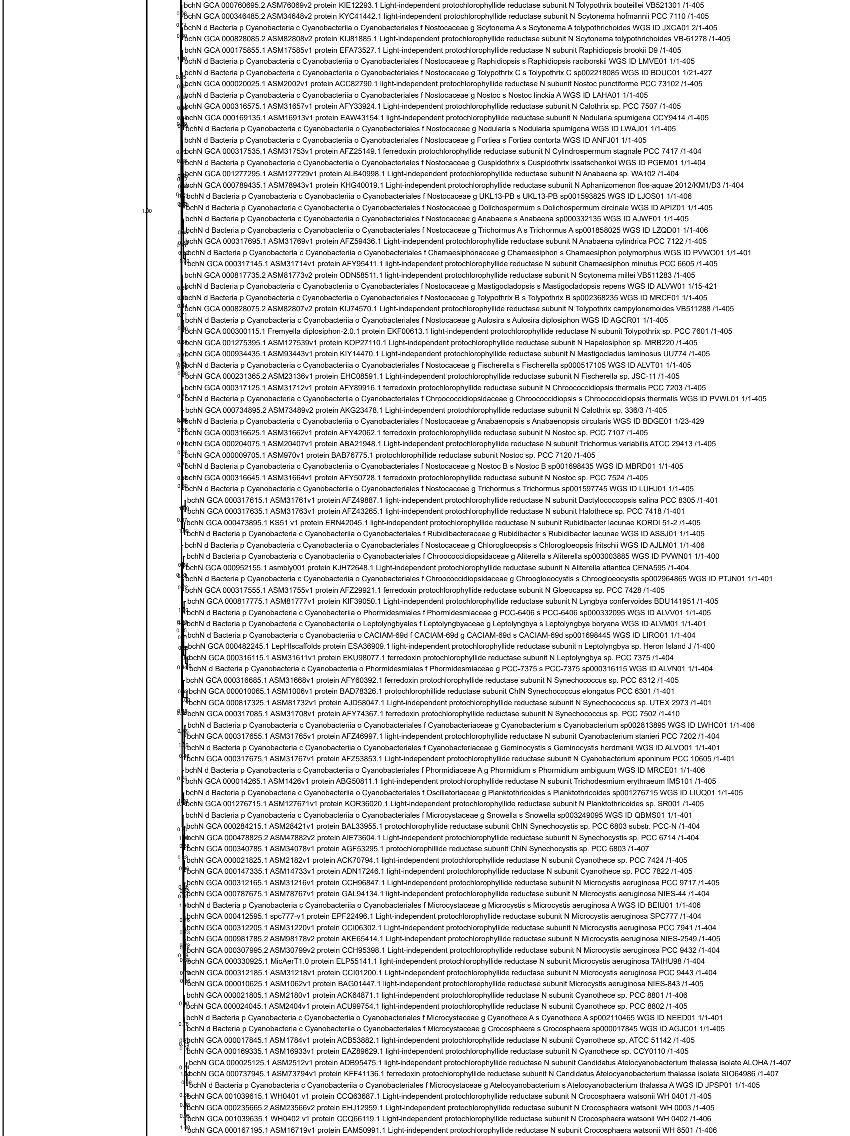
bchN GCA 000760695.2 ASM76069v2 protein KIE12293.1 Light-independent protochlorophyllide reductase subunit N Tolypothrix bouteillei VB521301 /1-405
bchN GCA 000346485.2 ASM34648v2 protein KYC41442.1 light-independent protochlorophyllide reductase subunit N Scytonema hofmannii PCC 7110 /1-405
bchN d Bacteria p Cyanobacteria c Cyanobacteriia o Cyanobacteriales f Nostocaceae g Scytonema A s Scytonema A tolypothrichoides WGS ID JXCA01 2/1-405
bchN GCA 000828085.2 ASM82808v2 protein KIJ81885.1 Light-independent protochlorophyllide reductase subunit N Scytonema tolypothrichoides VB-61278 /1-405
bchN GCA 000175855.1 ASM17585v1 protein EFA73527.1 Light-independent protochlorophyllide reductase subunit N Raphidiopsis brookii D9 /1-405
bchN d Bacteria p Cyanobacteria c Cyanobacteriia o Cyanobacteriales f Nostocaceae g Raphidiopsis s Raphidiopsis raciborskii WGS ID LMVE01 1/1-405
bchN d Bacteria p Cyanobacteria c Cyanobacteriia o Cyanobacteriales f Nostocaceae g Tolypothrix C s Tolypothrix C sp002218085 WGS ID BDUC01 1/21-427
bchN GCA 000020025.1 ASM2002v1 protein ACC82790.1 light-independent protochlorophyllide reductase N subunit Nostoc punctiforme PCC 73102 /1-405
bchN d Bacteria p Cyanobacteria c Cyanobacteriia o Cyanobacteriales f Nostocaceae g Nostoc s Nostoc linckia A WGS ID LAHA01 1/1-405
bchN GCA 000316575.1 ASM31657v1 protein AFY33924.1 Light-independent protochlorophyllide reductase subunit N Calothrix sp. PCC 7507 /1-405
bchN GCA 000169135.1 ASM16913v1 protein EAW43154.1 light-independent protochlorophyllide reductase subunit N Nodularia spumigena CCY9414 /1-405
bchN d Bacteria p Cyanobacteria c Cyanobacteriia o Cyanobacteriales f Nostocaceae g Nodularia s Nodularia spumigena WGS ID LWAJ01 1/1-405
bchN d Bacteria p Cyanobacteria c Cyanobacteriia o Cyanobacteriales f Nostocaceae g Fortiea s Fortiea contorta WGS ID ANFJ01 1/1-405
bchN GCA 000317535.1 ASM31753v1 protein AFZ25149.1 ferredoxin protochlorophyllide reductase subunit N Cylindrospermum stagnale PCC 7417 /1-404
bchN d Bacteria p Cyanobacteria c Cyanobacteriia o Cyanobacteriales f Nostocaceae g Cuspidothrix s Cuspidothrix issatschenkoi WGS ID PGEM01 1/1-404
bchN GCA 001277295.1 ASM127729v1 protein ALB40998.1 Light-independent protochlorophyllide reductase subunit N Anabaena sp. WA102 /1-404
bchN GCA 000789435.1 ASM78943v1 protein KHG40019.1 Light-independent protochlorophyllide reductase subunit N Aphanizomenon flos-aquae 2012/KM1/D3 /1-404
bchN d Bacteria p Cyanobacteria c Cyanobacteriia o Cyanobacteriales f Nostocaceae g UKL13-PB s UKL13-PB sp001593825 WGS ID LJOS01 1/1-406
1.00
bchN d Bacteria p Cyanobacteria c Cyanobacteriia o Cyanobacteriales f Nostocaceae g Dolichospermum s Dolichospermum circinale WGS ID APIZ01 1/1-405
bchN d Bacteria p Cyanobacteria c Cyanobacteriia o Cyanobacteriales f Nostocaceae g Anabaena s Anabaena sp000332135 WGS ID AJWF01 1/1-405
bchN d Bacteria p Cyanobacteria c Cyanobacteriia o Cyanobacteriales f Nostocaceae g Trichormus A s Trichormus A sp001858025 WGS ID LZQD01 1/1-406
bchN GCA 000317695.1 ASM31769v1 protein AFZ59436.1 Light-independent protochlorophyllide reductase subunit N Anabaena cylindrica PCC 7122 /1-405
bchN d Bacteria p Cyanobacteria c Cyanobacteriia o Cyanobacteriales f Chamaesiphonaceae g Chamaesiphon s Chamaesiphon polymorphus WGS ID PVWO01 1/1-401
bchN GCA 000317145.1 ASM31714v1 protein AFY95411.1 light-independent protochlorophyllide reductase N subunit Chamaesiphon minutus PCC 6605 /1-405
bchN GCA 000817735.2 ASM81773v2 protein ODN58511.1 light-independent protochlorophyllide reductase subunit N Scytonema millei VB511283 /1-405
bchN d Bacteria p Cyanobacteria c Cyanobacteriia o Cyanobacteriales f Nostocaceae g Mastigocladopsis s Mastigocladopsis repens WGS ID ALVW01 1/15-421
bchN d Bacteria p Cyanobacteria c Cyanobacteriia o Cyanobacteriales f Nostocaceae g Tolypothrix B s Tolypothrix B sp002368235 WGS ID MRCF01 1/1-405
bchN GCA 000828075.2 ASM82807v2 protein KIJ74570.1 Light-independent protochlorophyllide reductase subunit N Tolypothrix campylonemoides VB511288 /1-405
bchN d Bacteria p Cyanobacteria c Cyanobacteriia o Cyanobacteriales f Nostocaceae g Aulosira s Aulosira diplosiphon WGS ID AGCR01 1/1-405
bchN GCA 000300115.1 Fremyella diplosiphon-2.0.1 protein EKF00613.1 light-independent protochlorophyllide reductase N subunit Tolypothrix sp. PCC 7601 /1-405
bchN GCA 001275395.1 ASM127539v1 protein KOP27110.1 Light-independent protochlorophyllide reductase subunit N Hapalosiphon sp. MRB220 /1-405
bchN GCA 000934435.1 ASM93443v1 protein KIY14470.1 Light-independent protochlorophyllide reductase subunit N Mastigocladus laminosus UU774 /1-405
bchN d Bacteria p Cyanobacteria c Cyanobacteriia o Cyanobacteriales f Nostocaceae g Fischerella s Fischerella sp000517105 WGS ID ALVT01 1/1-405
bchN GCA 000231365.2 ASM23136v1 protein EHC08591.1 Light-independent protochlorophyllide reductase subunit N Fischerella sp. JSC-11 /1-405
bchN GCA 000317125.1 ASM31712v1 protein AFY89916.1 ferredoxin protochlorophyllide reductase subunit N Chroococcidiopsis thermalis PCC 7203 /1-405
bchN d Bacteria p Cyanobacteria c Cyanobacteriia o Cyanobacteriales f Chroococcidiopsidaceae g Chroococcidiopsis s Chroococcidiopsis thermalis WGS ID PVWL01 1/1-405
bchN GCA 000734895.2 ASM73489v2 protein AKG23478.1 Light-independent protochlorophyllide reductase subunit N Calothrix sp. 336/3 /1-405
bchN d Bacteria p Cyanobacteria c Cyanobacteriia o Cyanobacteriales f Nostocaceae g Anabaenopsis s Anabaenopsis circularis WGS ID BDGE01 1/23-429
bchN GCA 000316625.1 ASM31662v1 protein AFY42062.1 ferredoxin protochlorophyllide reductase subunit N Nostoc sp. PCC 7107 /1-405
bchN GCA 000204075.1 ASM20407v1 protein ABA21948.1 Light-independent protochlorophyllide reductase N subunit Trichormus variabilis ATCC 29413 /1-405
bchN GCA 000009705.1 ASM970v1 protein BAB76775.1 protochlorophillide reductase subunit Nostoc sp. PCC 7120 /1-405
bchN d Bacteria p Cyanobacteria c Cyanobacteriia o Cyanobacteriales f Nostocaceae g Nostoc B s Nostoc B sp001698435 WGS ID MBRD01 1/1-405
bchN GCA 000316645.1 ASM31664v1 protein AFY50728.1 ferredoxin protochlorophyllide reductase subunit N Nostoc sp. PCC 7524 /1-405
bchN d Bacteria p Cyanobacteria c Cyanobacteriia o Cyanobacteriales f Nostocaceae g Trichormus s Trichormus sp001597745 WGS ID LUHJ01 1/1-405
bchN GCA 000317615.1 ASM31761v1 protein AFZ49887.1 light-independent protochlorophyllide reductase N subunit Dactylococcopsis salina PCC 8305 /1-401
bchN GCA 000317635.1 ASM31763v1 protein AFZ43265.1 light-independent protochlorophyllide reductase N subunit Halothece sp. PCC 7418 /1-401
bchN GCA 000473895.1 KS51 v1 protein ERN42045.1 light-independent protochlorophyllide reductase N subunit Rubidibacter lacunae KORDI 51-2 /1-405
bchN d Bacteria p Cyanobacteria c Cyanobacteriia o Cyanobacteriales f Rubidibacteraceae g Rubidibacter s Rubidibacter lacunae WGS ID ASSJ01 1/1-405
bchN d Bacteria p Cyanobacteria c Cyanobacteriia o Cyanobacteriales f Nostocaceae g Chlorogloeopsis s Chlorogloeopsis fritschii WGS ID AJLM01 1/1-406
bchN d Bacteria p Cyanobacteria c Cyanobacteriia o Cyanobacteriales f Chroococcidiopsidaceae g Aliterella s Aliterella sp003003885 WGS ID PVWN01 1/1-400
bchN GCA 000952155.1 asmbly001 protein KJH72648.1 Light-independent protochlorophyllide reductase subunit N Aliterella atlantica CENA595 /1-404
bchN d Bacteria p Cyanobacteria c Cyanobacteriia o Cyanobacteriales f Chroococcidiopsidaceae g Chroogloeocystis s Chroogloeocystis sp002964865 WGS ID PTJN01 1/1-401
bchN GCA 000317555.1 ASM31755v1 protein AFZ29921.1 ferredoxin protochlorophyllide reductase subunit N Gloeocapsa sp. PCC 7428 /1-405
bchN GCA 000817775.1 ASM81777v1 protein KIF39050.1 Light-independent protochlorophyllide reductase subunit N Lyngbya confervoides BDU141951 /1-405
bchN d Bacteria p Cyanobacteria c Cyanobacteriia o Phormidesmiales f Phormidesmiaceae g PCC-6406 s PCC-6406 sp000332095 WGS ID ALVV01 1/1-405
bchN d Bacteria p Cyanobacteria c Cyanobacteriia o Leptolyngbyales f Leptolyngbyaceae g Leptolyngbya s Leptolyngbya boryana WGS ID ALVM01 1/1-401
bchN d Bacteria p Cyanobacteria c Cyanobacteriia o CACIAM-69d f CACIAM-69d g CACIAM-69d s CACIAM-69d sp001698445 WGS ID LIRO01 1/1-404
bchN GCA 000482245.1 LepHIscaffolds protein ESA36909.1 light-independent protochlorophyllide reductase subunit n Leptolyngbya sp. Heron Island J /1-400
bchN GCA 000316115.1 ASM31611v1 protein EKU98077.1 ferredoxin protochlorophyllide reductase subunit N Leptolyngbya sp. PCC 7375 /1-404
bchN d Bacteria p Cyanobacteria c Cyanobacteriia o Phormidesmiales f Phormidesmiaceae g PCC-7375 s PCC-7375 sp000316115 WGS ID ALVN01 1/1-404
bchN GCA 000316685.1 ASM31668v1 protein AFY60392.1 ferredoxin protochlorophyllide reductase subunit N Synechococcus sp. PCC 6312 /1-405
bchN GCA 000010065.1 ASM1006v1 protein BAD78326.1 protochlorophillide reductase subunit ChlN Synechococcus elongatus PCC 6301 /1-401
bchN GCA 000817325.1 ASM81732v1 protein AJD58047.1 Light-independent protochlorophyllide reductase subunit N Synechococcus sp. UTEX 2973 /1-401
bchN GCA 000317085.1 ASM31708v1 protein AFY74367.1 ferredoxin protochlorophyllide reductase subunit N Synechococcus sp. PCC 7502 /1-410
bchN d Bacteria p Cyanobacteria c Cyanobacteriia o Cyanobacteriales f Cyanobacteriaceae g Cyanobacterium s Cyanobacterium sp002813895 WGS ID LWHC01 1/1-406
bchN GCA 000317655.1 ASM31765v1 protein AFZ46997.1 light-independent protochlorophyllide reductase N subunit Cyanobacterium stanieri PCC 7202 /1-404
bchN d Bacteria p Cyanobacteria c Cyanobacteriia o Cyanobacteriales f Cyanobacteriaceae g Geminocystis s Geminocystis herdmanii WGS ID ALVO01 1/1-401
bchN GCA 000317675.1 ASM31767v1 protein AFZ53853.1 Light-independent protochlorophyllide reductase subunit N Cyanobacterium aponinum PCC 10605 /1-401
bchN d Bacteria p Cyanobacteria c Cyanobacteriia o Cyanobacteriales f Phormidiaceae A g Phormidium s Phormidium ambiguum WGS ID MRCE01 1/1-406
bchN GCA 000014265.1 ASM1426v1 protein ABG50811.1 light-independent protochlorophyllide reductase N subunit Trichodesmium erythraeum IMS101 /1-405
bchN d Bacteria p Cyanobacteria c Cyanobacteriia o Cyanobacteriales f Oscillatoriaceae g Planktothricoides s Planktothricoides sp001276715 WGS ID LIUQ01 1/1-405
bchN GCA 001276715.1 ASM127671v1 protein KOR36020.1 Light-independent protochlorophyllide reductase subunit N Planktothricoides sp. SR001 /1-405
bchN d Bacteria p Cyanobacteria c Cyanobacteriia o Cyanobacteriales f Microcystaceae g Snowella s Snowella sp003249095 WGS ID QBMS01 1/1-401
bchN GCA 000284215.1 ASM28421v1 protein BAL33955.1 protochlorophyllide reductase subunit ChlN Synechocystis sp. PCC 6803 substr. PCC-N /1-404
bchN GCA 000478825.2 ASM47882v2 protein AIE73604.1 Light-independent protochlorophyllide reductase subunit N Synechocystis sp. PCC 6714 /1-404
bchN GCA 000340785.1 ASM34078v1 protein AGF53295.1 protochlorophillide reductase subunit ChlN Synechocystis sp. PCC 6803 /1-407
bchN GCA 000021825.1 ASM2182v1 protein ACK70794.1 light-independent protochlorophyllide reductase N subunit Cyanothece sp. PCC 7424 /1-405
bchN GCA 000147335.1 ASM14733v1 protein ADN17246.1 light-independent protochlorophyllide reductase N subunit Cyanothece sp. PCC 7822 /1-405
bchN GCA 000312165.1 ASM31216v1 protein CCH96847.1 Light-independent protochlorophyllide reductase subunit N Microcystis aeruginosa PCC 9717 /1-405
bchN GCA 000787675.1 ASM78767v1 protein GAL94134.1 Light-independent protochlorophyllide reductase subunit N Microcystis aeruginosa NIES-44 /1-404
bchN d Bacteria p Cyanobacteria c Cyanobacteriia o Cyanobacteriales f Microcystaceae g Microcystis s Microcystis aeruginosa A WGS ID BEIU01 1/1-406
bchN GCA 000412595.1 spc777-v1 protein EPF22496.1 Light-independent protochlorophyllide reductase subunit N Microcystis aeruginosa SPC777 /1-404
bchN GCA 000312205.1 ASM31220v1 protein CCI06302.1 Light-independent protochlorophyllide reductase subunit N Microcystis aeruginosa PCC 7941 /1-404
bchN GCA 000981785.2 ASM98178v2 protein AKE65414.1 Light-independent protochlorophyllide reductase subunit N Microcystis aeruginosa NIES-2549 /1-405
bchN GCA 000307995.2 ASM30799v2 protein CCH95398.1 Light-independent protochlorophyllide reductase subunit N Microcystis aeruginosa PCC 9432 /1-404
bchN GCA 000330925.1 MicAerT1.0 protein ELP55141.1 light-independent protochlorophyllide reductase N subunit Microcystis aeruginosa TAIHU98 /1-404
bchN GCA 000312185.1 ASM31218v1 protein CCI01200.1 Light-independent protochlorophyllide reductase subunit N Microcystis aeruginosa PCC 9443 /1-404
bchN GCA 000010625.1 ASM1062v1 protein BAG01447.1 light-independent protochlorophyllide reductase subunit Microcystis aeruginosa NIES-843 /1-405
bchN GCA 000021805.1 ASM2180v1 protein ACK64871.1 light-independent protochlorophyllide reductase N subunit Cyanothece sp. PCC 8801 /1-406
bchN GCA 000024045.1 ASM2404v1 protein ACU99754.1 light-independent protochlorophyllide reductase N subunit Cyanothece sp. PCC 8802 /1-405
bchN d Bacteria p Cyanobacteria c Cyanobacteriia o Cyanobacteriales f Microcystaceae g Cyanothece A s Cyanothece A sp002110465 WGS ID NEED01 1/1-401
bchN d Bacteria p Cyanobacteria c Cyanobacteriia o Cyanobacteriales f Microcystaceae g Crocosphaera s Crocosphaera sp000017845 WGS ID AGJC01 1/1-405
bchN GCA 000017845.1 ASM1784v1 protein ACB53882.1 light-independent protochlorophyllide reductase N subunit Cyanothece sp. ATCC 51142 /1-405
bchN GCA 000169335.1 ASM16933v1 protein EAZ89629.1 light-independent protochlorophyllide reductase subunit N Cyanothece sp. CCY0110 /1-405
bchN GCA 000025125.1 ASM2512v1 protein ADB95475.1 light-independent protochlorophyllide reductase N subunit Candidatus Atelocyanobacterium thalassa isolate ALOHA /1-407
bchN GCA 000737945.1 ASM73794v1 protein KFF41136.1 ferredoxin protochlorophyllide reductase subunit N Candidatus Atelocyanobacterium thalassa isolate SIO64986 /1-407
bchN d Bacteria p Cyanobacteria c Cyanobacteriia o Cyanobacteriales f Microcystaceae g Atelocyanobacterium s Atelocyanobacterium thalassa A WGS ID JPSP01 1/1-405
bchN GCA 001039615.1 WH0401 v1 protein CCQ63687.1 Light-independent protochlorophyllide reductase subunit N Crocosphaera watsonii WH 0401 /1-405
bchN GCA 000235665.2 ASM23566v2 protein EHJ12959.1 Light-independent protochlorophyllide reductase subunit N Crocosphaera watsonii WH 0003 /1-405
bchN GCA 001039635.1 WH0402 v1 protein CCQ66119.1 Light-independent protochlorophyllide reductase subunit N Crocosphaera watsonii WH 0402 /1-406
bchN GCA 000167195.1 ASM16719v1 protein EAM50991.1 Light-independent protochlorophyllide reductase N subunit Crocosphaera watsonii WH 8501 /1-406

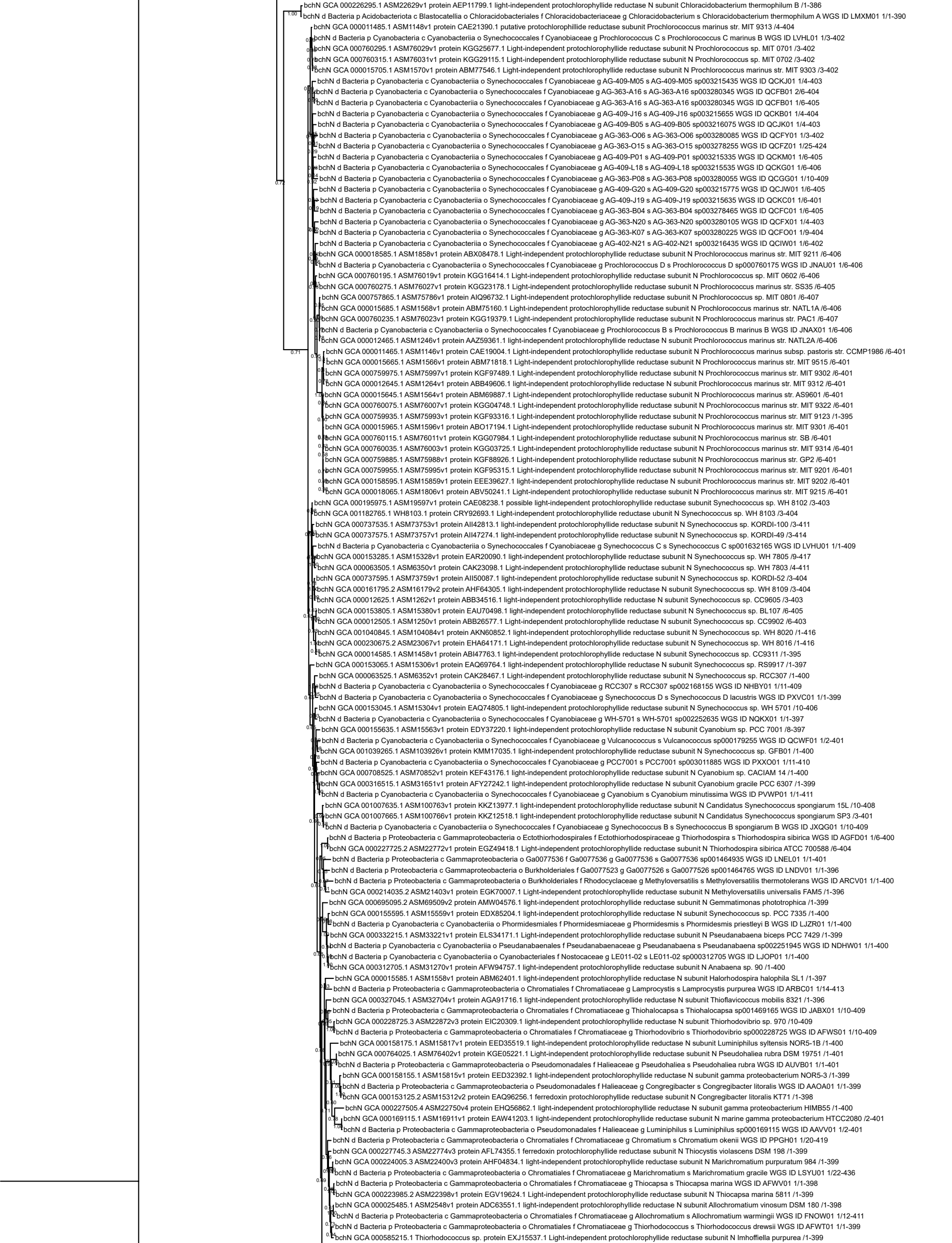

bchN GCA 000226295.1 ASM22629v1 protein AEP11799.1 light-independent protochlorophyllide reductase N subunit Chloracidobacterium thermophilum B /1-386
bchN d Bacteria p Acidobacteriota c Blastocatellia o Chloracidobacteriales f Chloracidobacteriaceae g Chloracidobacterium s Chloracidobacterium thermophilum A WGS ID LMXM01 1/1-390
bchN GCA 000011485.1 ASM1148v1 protein CAE21390.1 putative protochlorophyllide reductase subunit Prochlorococcus marinus str. MIT 9313 /4-404
bchN d Bacteria p Cyanobacteria c Cyanobacteriia o Synechococcales f Cyanobiaceae g Prochlorococcus C s Prochlorococcus C marinus B WGS ID LVHL01 1/3-402
bchN GCA 000760295.1 ASM76029v1 protein KGG25677.1 Light-independent protochlorophyllide reductase subunit N Prochlorococcus sp. MIT 0701 /3-402
bchN GCA 000760315.1 ASM76031v1 protein KGG29115.1 Light-independent protochlorophyllide reductase subunit N Prochlorococcus sp. MIT 0702 /3-402
bchN GCA 000015705.1 ASM1570v1 protein ABM77546.1 Light-independent protochlorophyllide reductase subunit N Prochlorococcus marinus str. MIT 9303 /3-402
bchN d Bacteria p Cyanobacteria c Cyanobacteriia o Synechococcales f Cyanobiaceae g AG-409-M05 s AG-409-M05 sp003215435 WGS ID QCKJ01 1/4-403
bchN d Bacteria p Cyanobacteria c Cyanobacteriia o Synechococcales f Cyanobiaceae g AG-363-A16 s AG-363-A16 sp003280345 WGS ID QCFB01 2/6-404
bchN d Bacteria p Cyanobacteria c Cyanobacteriia o Synechococcales f Cyanobiaceae g AG-363-A16 s AG-363-A16 sp003280345 WGS ID QCFB01 1/6-405
bchN d Bacteria p Cyanobacteria c Cyanobacteriia o Synechococcales f Cyanobiaceae g AG-409-J16 s AG-409-J16 sp003215655 WGS ID QCKB01 1/4-404
bchN d Bacteria p Cyanobacteria c Cyanobacteriia o Synechococcales f Cyanobiaceae g AG-409-B05 s AG-409-B05 sp003216075 WGS ID QCJK01 1/4-403
bchN d Bacteria p Cyanobacteria c Cyanobacteriia o Synechococcales f Cyanobiaceae g AG-363-O06 s AG-363-O06 sp003280085 WGS ID QCFY01 1/3-402
bchN d Bacteria p Cyanobacteria c Cyanobacteriia o Synechococcales f Cyanobiaceae g AG-363-O15 s AG-363-O15 sp003278255 WGS ID QCFZ01 1/25-424
bchN d Bacteria p Cyanobacteria c Cyanobacteriia o Synechococcales f Cyanobiaceae g AG-409-P01 s AG-409-P01 sp003215335 WGS ID QCKM01 1/6-405
bchN d Bacteria p Cyanobacteria c Cyanobacteriia o Synechococcales f Cyanobiaceae g AG-409-L18 s AG-409-L18 sp003215535 WGS ID QCKG01 1/6-406
bchN d Bacteria p Cyanobacteria c Cyanobacteriia o Synechococcales f Cyanobiaceae g AG-363-P08 s AG-363-P08 sp003280055 WGS ID QCGG01 1/10-409
bchN d Bacteria p Cyanobacteria c Cyanobacteriia o Synechococcales f Cyanobiaceae g AG-409-G20 s AG-409-G20 sp003215775 WGS ID QCJW01 1/6-405
bchN d Bacteria p Cyanobacteria c Cyanobacteriia o Synechococcales f Cyanobiaceae g AG-409-J19 s AG-409-J19 sp003215635 WGS ID QCKC01 1/6-401
bchN d Bacteria p Cyanobacteria c Cyanobacteriia o Synechococcales f Cyanobiaceae g AG-363-B04 s AG-363-B04 sp003278465 WGS ID QCFC01 1/6-405
bchN d Bacteria p Cyanobacteria c Cyanobacteriia o Synechococcales f Cyanobiaceae g AG-363-N20 s AG-363-N20 sp003280105 WGS ID QCFX01 1/4-403
bchN d Bacteria p Cyanobacteria c Cyanobacteriia o Synechococcales f Cyanobiaceae g AG-363-K07 s AG-363-K07 sp003280225 WGS ID QCFO01 1/9-404
bchN d Bacteria p Cyanobacteria c Cyanobacteriia o Synechococcales f Cyanobiaceae g AG-402-N21 s AG-402-N21 sp003216435 WGS ID QCIW01 1/6-402
bchN GCA 000018585.1 ASM1858v1 protein ABX08478.1 Light-independent protochlorophyllide reductase subunit N Prochlorococcus marinus str. MIT 9211 /6-406
bchN d Bacteria p Cyanobacteria c Cyanobacteriia o Synechococcales f Cyanobiaceae g Prochlorococcus D s Prochlorococcus D sp000760175 WGS ID JNAU01 1/6-406
bchN GCA 000760195.1 ASM76019v1 protein KGG16414.1 Light-independent protochlorophyllide reductase subunit N Prochlorococcus sp. MIT 0602 /6-406
bchN GCA 000760275.1 ASM76027v1 protein KGG23178.1 Light-independent protochlorophyllide reductase subunit N Prochlorococcus marinus str. SS35 /6-405
bchN GCA 000757865.1 ASM75786v1 protein AIQ96732.1 Light-independent protochlorophyllide reductase subunit N Prochlorococcus sp. MIT 0801 /6-407
bchN GCA 000015685.1 ASM1568v1 protein ABM75160.1 Light-independent protochlorophyllide reductase subunit N Prochlorococcus marinus str. NATL1A /6-406
bchN GCA 000760235.1 ASM76023v1 protein KGG19379.1 Light-independent protochlorophyllide reductase subunit N Prochlorococcus marinus str. PAC1 /6-407
bchN d Bacteria p Cyanobacteria c Cyanobacteriia o Synechococcales f Cyanobiaceae g Prochlorococcus B s Prochlorococcus B marinus B WGS ID JNAX01 1/6-406
bchN GCA 000012465.1 ASM1246v1 protein AAZ59361.1 light-independent protochlorophyllide reductase N subunit Prochlorococcus marinus str. NATL2A /6-406
bchN GCA 000011465.1 ASM1146v1 protein CAE19004.1 Light-independent protochlorophyllide reductase subunit N Prochlorococcus marinus subsp. pastoris str. CCMP1986 /6-401
bchN GCA 000015665.1 ASM1566v1 protein ABM71818.1 Light-independent protochlorophyllide reductase subunit N Prochlorococcus marinus str. MIT 9515 /6-401
bchN GCA 000759975.1 ASM75997v1 protein KGF97489.1 Light-independent protochlorophyllide reductase subunit N Prochlorococcus marinus str. MIT 9302 /6-401
bchN GCA 000012645.1 ASM1264v1 protein ABB49606.1 light-independent protochlorophyllide reductase N subunit Prochlorococcus marinus str. MIT 9312 /6-401
bchN GCA 000015645.1 ASM1564v1 protein ABM69887.1 Light-independent protochlorophyllide reductase subunit N Prochlorococcus marinus str. AS9601 /6-401
bchN GCA 000760075.1 ASM76007v1 protein KGG04748.1 Light-independent protochlorophyllide reductase subunit N Prochlorococcus marinus str. MIT 9322 /6-401
bchN GCA 000759935.1 ASM75993v1 protein KGF93316.1 Light-independent protochlorophyllide reductase subunit N Prochlorococcus marinus str. MIT 9123 /1-395
bchN GCA 000015965.1 ASM1596v1 protein ABO17194.1 Light-independent protochlorophyllide reductase subunit N Prochlorococcus marinus str. MIT 9301 /6-401
bchN GCA 000760115.1 ASM76011v1 protein KGG07984.1 Light-independent protochlorophyllide reductase subunit N Prochlorococcus marinus str. SB /6-401
bchN GCA 000760035.1 ASM76003v1 protein KGG03725.1 Light-independent protochlorophyllide reductase subunit N Prochlorococcus marinus str. MIT 9314 /6-401
bchN GCA 000759885.1 ASM75988v1 protein KGF88926.1 Light-independent protochlorophyllide reductase subunit N Prochlorococcus marinus str. GP2 /6-401
bchN GCA 000759955.1 ASM75995v1 protein KGF95315.1 Light-independent protochlorophyllide reductase subunit N Prochlorococcus marinus str. MIT 9201 /6-401
bchN GCA 000158595.1 ASM15859v1 protein EEE39627.1 light-independent protochlorophyllide reductase N subunit Prochlorococcus marinus str. MIT 9202 /6-401
bchN GCA 000018065.1 ASM1806v1 protein ABV50241.1 Light-independent protochlorophyllide reductase subunit N Prochlorococcus marinus str. MIT 9215 /6-401
bchN GCA 000195975.1 ASM19597v1 protein CAE08238.1 possible light-independent protochlorophyllide reductase subunit Synechococcus sp. WH 8102 /3-403
bchN GCA 001182765.1 WH8103.1 protein CRY92693.1 Light-independent protochlorophyllide reductase ubunit N Synechococcus sp. WH 8103 /3-404
bchN GCA 000737535.1 ASM73753v1 protein AII42813.1 light-independent protochlorophyllide reductase subunit N Synechococcus sp. KORDI-100 /3-411
bchN GCA 000737575.1 ASM73757v1 protein AII47274.1 light-independent protochlorophyllide reductase subunit N Synechococcus sp. KORDI-49 /3-414
bchN d Bacteria p Cyanobacteria c Cyanobacteriia o Synechococcales f Cyanobiaceae g Synechococcus C s Synechococcus C sp001632165 WGS ID LVHU01 1/1-409
bchN GCA 000153285.1 ASM15328v1 protein EAR20090.1 light-independent protochlorophyllide reductase subunit N Synechococcus sp. WH 7805 /9-417
bchN GCA 000063505.1 ASM6350v1 protein CAK23098.1 Light-independent protochlorophyllide reductase subunit N Synechococcus sp. WH 7803 /4-411
bchN GCA 000737595.1 ASM73759v1 protein AII50087.1 light-independent protochlorophyllide reductase subunit N Synechococcus sp. KORDI-52 /3-404
bchN GCA 000161795.2 ASM16179v2 protein AHF64305.1 light-independent protochlorophyllide reductase subunit N Synechococcus sp. WH 8109 /3-404
bchN GCA 000012625.1 ASM1262v1 protein ABB34516.1 light-independent protochlorophyllide reductase N subunit Synechococcus sp. CC9605 /3-403
bchN GCA 000153805.1 ASM15380v1 protein EAU70498.1 light-independent protochlorophyllide reductase subunit N Synechococcus sp. BL107 /6-405
bchN GCA 000012505.1 ASM1250v1 protein ABB26577.1 light-independent protochlorophyllide reductase N subunit Synechococcus sp. CC9902 /6-403
bchN GCA 001040845.1 ASM104084v1 protein AKN60852.1 light-independent protochlorophyllide reductase subunit N Synechococcus sp. WH 8020 /1-416
bchN GCA 000230675.2 ASM23067v1 protein EHA64171.1 light-independent protochlorophyllide reductase subunit N Synechococcus sp. WH 8016 /1-416
bchN GCA 000014585.1 ASM1458v1 protein ABI47763.1 light-independent protochlorophyllide reductase N subunit Synechococcus sp. CC9311 /1-395
bchN GCA 000153065.1 ASM15306v1 protein EAQ69764.1 light-independent protochlorophyllide reductase N subunit Synechococcus sp. RS9917 /1-397
bchN GCA 000063525.1 ASM6352v1 protein CAK28467.1 Light-independent protochlorophyllide reductase subunit N Synechococcus sp. RCC307 /1-400
bchN d Bacteria p Cyanobacteria c Cyanobacteriia o Synechococcales f Cyanobiaceae g RCC307 s RCC307 sp002168155 WGS ID NHBY01 1/11-409
bchN d Bacteria p Cyanobacteria c Cyanobacteriia o Synechococcales f Cyanobiaceae g Synechococcus D s Synechococcus D lacustris WGS ID PXVC01 1/1-399
bchN GCA 000153045.1 ASM15304v1 protein EAQ74805.1 light-independent protochlorophyllide reductase subunit N Synechococcus sp. WH 5701 /10-406
bchN d Bacteria p Cyanobacteria c Cyanobacteriia o Synechococcales f Cyanobiaceae g WH-5701 s WH-5701 sp002252635 WGS ID NQKX01 1/1-397
bchN GCA 000155635.1 ASM15563v1 protein EDY37220.1 light-independent protochlorophyllide reductase N subunit Cyanobium sp. PCC 7001 /8-397
bchN d Bacteria p Cyanobacteria c Cyanobacteriia o Synechococcales f Cyanobiaceae g Vulcanococcus s Vulcanococcus sp000179255 WGS ID QCWF01 1/2-401
bchN GCA 001039265.1 ASM103926v1 protein KMM17035.1 light-independent protochlorophyllide reductase subunit N Synechococcus sp. GFB01 /1-400
bchN d Bacteria p Cyanobacteria c Cyanobacteriia o Synechococcales f Cyanobiaceae g PCC7001 s PCC7001 sp003011885 WGS ID PXXO01 1/11-410
bchN GCA 000708525.1 ASM70852v1 protein KEF43176.1 light-independent protochlorophyllide reductase subunit N Cyanobium sp. CACIAM 14 /1-400
bchN GCA 000316515.1 ASM31651v1 protein AFY27242.1 light-independent protochlorophyllide reductase N subunit Cyanobium gracile PCC 6307 /1-399
bchN d Bacteria p Cyanobacteria c Cyanobacteriia o Synechococcales f Cyanobiaceae g Cyanobium s Cyanobium minutissima WGS ID PVWP01 1/1-411
bchN GCA 001007635.1 ASM100763v1 protein KKZ13977.1 light-independent protochlorophyllide reductase subunit N Candidatus Synechococcus spongiarum 15L /10-408
bchN GCA 001007665.1 ASM100766v1 protein KKZ12518.1 light-independent protochlorophyllide reductase subunit N Candidatus Synechococcus spongiarum SP3 /3-401
bchN d Bacteria p Cyanobacteria c Cyanobacteriia o Synechococcales f Cyanobiaceae g Synechococcus B s Synechococcus B spongiarum B WGS ID JXQG01 1/10-409
bchN d Bacteria p Proteobacteria c Gammaproteobacteria o Ectothiorhodospirales f Ectothiorhodospiraceae g Thiorhodospira s Thiorhodospira sibirica WGS ID AGFD01 1/6-400
bchN GCA 000227725.2 ASM22772v1 protein EGZ49418.1 Light-independent protochlorophyllide reductase subunit N Thiorhodospira sibirica ATCC 700588 /6-404
bchN d Bacteria p Proteobacteria c Gammaproteobacteria o Ga0077536 f Ga0077536 g Ga0077536 s Ga0077536 sp001464935 WGS ID LNEL01 1/1-401
bchN d Bacteria p Proteobacteria c Gammaproteobacteria o Burkholderiales f Ga0077523 g Ga0077526 s Ga0077526 sp001464765 WGS ID LNDV01 1/1-396
bchN d Bacteria p Proteobacteria c Gammaproteobacteria o Burkholderiales f Rhodocyclaceae g Methyloversatilis s Methyloversatilis thermotolerans WGS ID ARCV01 1/1-400
bchN GCA 000214035.2 ASM21403v1 protein EGK70007.1 Light-independent protochlorophyllide reductase subunit N Methyloversatilis universalis FAM5 /1-396
bchN GCA 000695095.2 ASM69509v2 protein AMW04576.1 light-independent protochlorophyllide reductase subunit N Gemmatimonas phototrophica /1-399
bchN GCA 000155595.1 ASM15559v1 protein EDX85204.1 light-independent protochlorophyllide reductase N subunit Synechococcus sp. PCC 7335 /1-400
bchN d Bacteria p Cyanobacteria c Cyanobacteriia o Phormidesmiales f Phormidesmiaceae g Phormidesmis s Phormidesmis priestleyi B WGS ID LJZR01 1/1-400
bchN GCA 000332215.1 ASM33221v1 protein ELS34171.1 Light-independent protochlorophyllide reductase subunit N Pseudanabaena biceps PCC 7429 /1-399
bchN d Bacteria p Cyanobacteria c Cyanobacteriia o Pseudanabaenales f Pseudanabaenaceae g Pseudanabaena s Pseudanabaena sp002251945 WGS ID NDHW01 1/1-400
bchN d Bacteria p Cyanobacteria c Cyanobacteriia o Cyanobacteriales f Nostocaceae g LE011-02 s LE011-02 sp000312705 WGS ID LJOP01 1/1-400
bchN GCA 000312705.1 ASM31270v1 protein AFW94757.1 light-independent protochlorophyllide reductase subunit N Anabaena sp. 90 /1-400
bchN GCA 000015585.1 ASM1558v1 protein ABM62401.1 light-independent protochlorophyllide reductase N subunit Halorhodospira halophila SL1 /1-397
bchN d Bacteria p Proteobacteria c Gammaproteobacteria o Chromatiales f Chromatiaceae g Lamprocystis s Lamprocystis purpurea WGS ID ARBC01 1/14-413
bchN GCA 000327045.1 ASM32704v1 protein AGA91716.1 light-independent protochlorophyllide reductase N subunit Thioflavicoccus mobilis 8321 /1-396
bchN d Bacteria p Proteobacteria c Gammaproteobacteria o Chromatiales f Chromatiaceae g Thiohalocapsa s Thiohalocapsa sp001469165 WGS ID JABX01 1/10-409
bchN GCA 000228725.3 ASM22872v3 protein EIC20309.1 light-independent protochlorophyllide reductase subunit N Thiorhodovibrio sp. 970 /10-409
bchN d Bacteria p Proteobacteria c Gammaproteobacteria o Chromatiales f Chromatiaceae g Thiorhodovibrio s Thiorhodovibrio sp000228725 WGS ID AFWS01 1/10-409
bchN GCA 000158175.1 ASM15817v1 protein EED35519.1 light-independent protochlorophyllide reductase N subunit Luminiphilus syltensis NOR5-1B /1-400
bchN GCA 000764025.1 ASM76402v1 protein KGE05221.1 Light-independent protochlorophyllide reductase subunit N Pseudohaliea rubra DSM 19751 /1-401
bchN d Bacteria p Proteobacteria c Gammaproteobacteria o Pseudomonadales f Halieaceae g Pseudohaliea s Pseudohaliea rubra WGS ID AUVB01 1/1-401
bchN GCA 000158155.1 ASM15815v1 protein EED32392.1 light-independent protochlorophyllide reductase N subunit gamma proteobacterium NOR5-3 /1-399
bchN d Bacteria p Proteobacteria c Gammaproteobacteria o Pseudomonadales f Halieaceae g Congregibacter s Congregibacter litoralis WGS ID AAOA01 1/1-399
bchN GCA 000153125.2 ASM15312v2 protein EAQ96256.1 ferredoxin protochlorophyllide reductase subunit N Congregibacter litoralis KT71 /1-398
bchN GCA 000227505.4 ASM22750v4 protein EHQ56862.1 light-independent protochlorophyllide reductase N subunit gamma proteobacterium HIMB55 /1-400
bchN GCA 000169115.1 ASM16911v1 protein EAW41203.1 light-independent protochlorophyllide reductase subunit N marine gamma proteobacterium HTCC2080 /2-401
bchN d Bacteria p Proteobacteria c Gammaproteobacteria o Pseudomonadales f Halieaceae g Luminiphilus s Luminiphilus sp000169115 WGS ID AAVV01 1/2-401
bchN d Bacteria p Proteobacteria c Gammaproteobacteria o Chromatiales f Chromatiaceae g Chromatium s Chromatium okenii WGS ID PPGH01 1/20-419
bchN GCA 000227745.3 ASM22774v3 protein AFL74355.1 ferredoxin protochlorophyllide reductase subunit N Thiocystis violascens DSM 198 /1-399
bchN GCA 000224005.3 ASM22400v3 protein AHF04834.1 light-independent protochlorophyllide reductase subunit N Marichromatium purpuratum 984 /1-399
bchN d Bacteria p Proteobacteria c Gammaproteobacteria o Chromatiales f Chromatiaceae g Marichromatium s Marichromatium gracile WGS ID LSYU01 1/22-436
bchN d Bacteria p Proteobacteria c Gammaproteobacteria o Chromatiales f Chromatiaceae g Thiocapsa s Thiocapsa marina WGS ID AFWV01 1/1-398
bchN GCA 000223985.2 ASM22398v1 protein EGV19624.1 Light-independent protochlorophyllide reductase subunit N Thiocapsa marina 5811 /1-399
bchN GCA 000025485.1 ASM2548v1 protein ADC63551.1 light-independent protochlorophyllide reductase N subunit Allochromatium vinosum DSM 180 /1-398
bchN d Bacteria p Proteobacteria c Gammaproteobacteria o Chromatiales f Chromatiaceae g Allochromatium s Allochromatium warmingii WGS ID FNOW01 1/12-411
bchN d Bacteria p Proteobacteria c Gammaproteobacteria o Chromatiales f Chromatiaceae g Thiorhodococcus s Thiorhodococcus drewsii WGS ID AFWT01 1/1-399
bchN GCA 000585215.1 Thiorhodococcus sp. protein EXJ15537.1 Light-independent protochlorophyllide reductase subunit N Imhoffiella purpurea /1-399

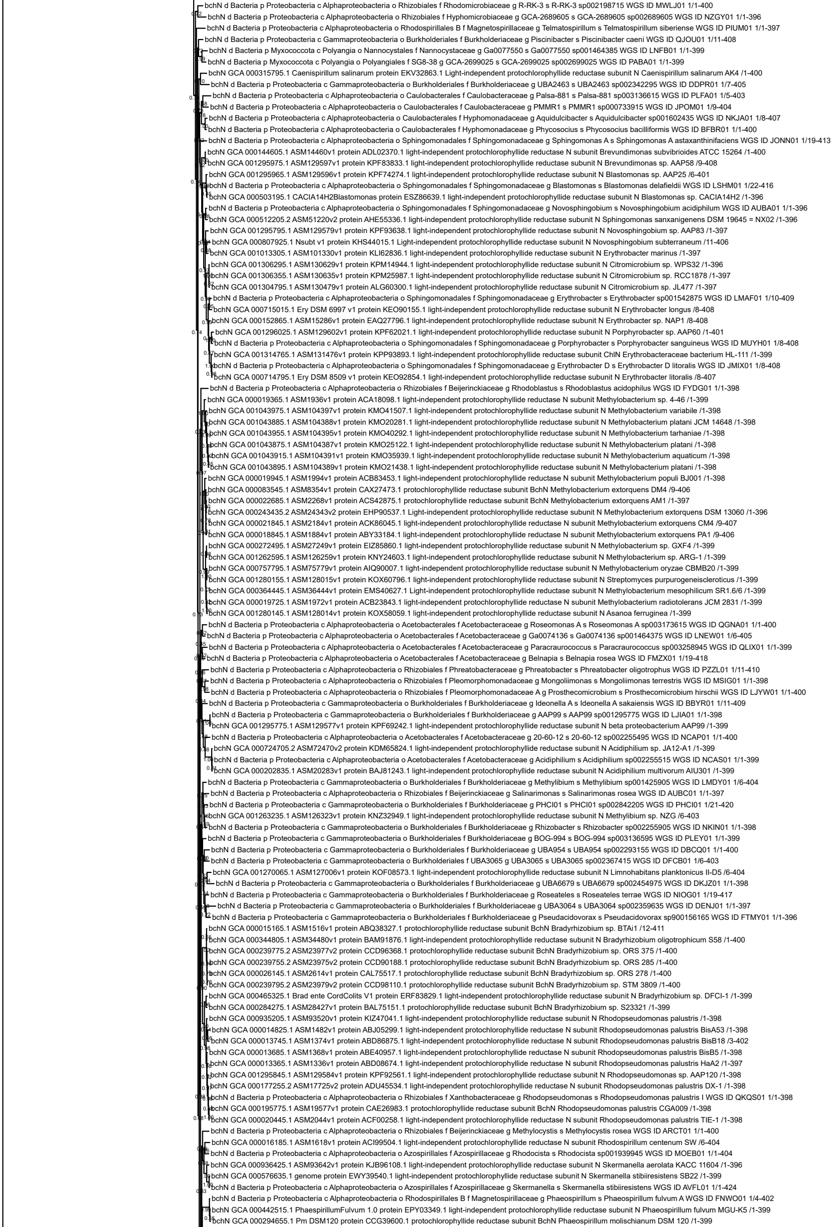
bchN d Bacteria p Proteobacteria c Alphaproteobacteria o Rhizobiales f Rhodomicrobiaceae g R-RK-3 s R-RK-3 sp002198715 WGS ID MWLJ01 1/1-400
bchN d Bacteria p Proteobacteria c Alphaproteobacteria o Rhizobiales f Hyphomicrobiaceae g GCA-2689605 s GCA-2689605 sp002689605 WGS ID NZGY01 1/1-396
bchN d Bacteria p Proteobacteria c Alphaproteobacteria o Rhodospirillales B f Magnetospirillaceae g Telmatospirillum s Telmatospirillum siberiense WGS ID PIUM01 1/1-397
bchN d Bacteria p Proteobacteria c Gammaproteobacteria o Burkholderiales f Burkholderiaceae g Piscinibacter s Piscinibacter caeni WGS ID QJOU01 1/11-408
bchN d Bacteria p Myxococcota c Polyangia o Nannocystales f Nannocystaceae g Ga0077550 s Ga0077550 sp001464385 WGS ID LNFB01 1/1-399
bchN d Bacteria p Myxococcota c Polyangia o Polyangiales f SG8-38 g GCA-2699025 s GCA-2699025 sp002699025 WGS ID PABA01 1/1-399
bchN GCA 000315795.1 Caenispirillum salinarum protein EKV32863.1 Light-independent protochlorophyllide reductase subunit N Caenispirillum salinarum AK4 /1-400
bchN d Bacteria p Proteobacteria c Gammaproteobacteria o Burkholderiales f Burkholderiaceae g UBA2463 s UBA2463 sp002342295 WGS ID DDPR01 1/7-405
bchN d Bacteria p Proteobacteria c Alphaproteobacteria o Caulobacterales f Caulobacteraceae g Palsa-881 s Palsa-881 sp003136615 WGS ID PLFA01 1/5-403
bchN d Bacteria p Proteobacteria c Alphaproteobacteria o Caulobacterales f Caulobacteraceae g PMMR1 s PMMR1 sp000733915 WGS ID JPOM01 1/9-404
bchN d Bacteria p Proteobacteria c Alphaproteobacteria o Caulobacterales f Hyphomonadaceae g Aquidulcibacter s Aquidulcibacter sp001602435 WGS ID NKJA01 1/8-407
bchN d Bacteria p Proteobacteria c Alphaproteobacteria o Caulobacterales f Hyphomonadaceae g Phycosocius s Phycosocius bacilliformis WGS ID BFBR01 1/1-400
bchN d Bacteria p Proteobacteria c Alphaproteobacteria o Sphingomonadales f Sphingomonadaceae g Sphingomonas A s Sphingomonas A astaxanthinifaciens WGS ID JONN01 1/19-413
bchN GCA 000144605.1 ASM14460v1 protein ADL02370.1 light-independent protochlorophyllide reductase N subunit Brevundimonas subvibrioides ATCC 15264 /1-400
bchN GCA 001295975.1 ASM129597v1 protein KPF83833.1 light-independent protochlorophyllide reductase subunit N Brevundimonas sp. AAP58 /9-408
bchN GCA 001295965.1 ASM129596v1 protein KPF74274.1 light-independent protochlorophyllide reductase subunit N Blastomonas sp. AAP25 /6-401
bchN d Bacteria p Proteobacteria c Alphaproteobacteria o Sphingomonadales f Sphingomonadaceae g Blastomonas s Blastomonas delafieldii WGS ID LSHM01 1/22-416
bchN GCA 000503195.1 CACIA14H2Blastomonas protein ESZ86639.1 light-independent protochlorophyllide reductase subunit N Blastomonas sp. CACIA14H2 /1-396
bchN d Bacteria p Proteobacteria c Alphaproteobacteria o Sphingomonadales f Sphingomonadaceae g Novosphingobium s Novosphingobium acidiphilum WGS ID AUBA01 1/1-396
bchN GCA 000512205.2 ASM51220v2 protein AHE55336.1 light-independent protochlorophyllide reductase subunit N Sphingomonas sanxanigenens DSM 19645 = NX02 /1-396
bchN GCA 001295795.1 ASM129579v1 protein KPF93638.1 light-independent protochlorophyllide reductase subunit N Novosphingobium sp. AAP83 /1-397
bchN GCA 000807925.1 Nsubt v1 protein KHS44015.1 Light-independent protochlorophyllide reductase subunit N Novosphingobium subterraneum /11-406
bchN GCA 001013305.1 ASM101330v1 protein KLI62836.1 light-independent protochlorophyllide reductase subunit N Erythrobacter marinus /1-397
bchN GCA 001306295.1 ASM130629v1 protein KPM14944.1 light-independent protochlorophyllide reductase subunit N Citromicrobium sp. WPS32 /1-396
bchN GCA 001306355.1 ASM130635v1 protein KPM25987.1 light-independent protochlorophyllide reductase subunit N Citromicrobium sp. RCC1878 /1-397
bchN GCA 001304795.1 ASM130479v1 protein ALG60300.1 light-independent protochlorophyllide reductase subunit N Citromicrobium sp. JL477 /1-397
bchN d Bacteria p Proteobacteria c Alphaproteobacteria o Sphingomonadales f Sphingomonadaceae g Erythrobacter s Erythrobacter sp001542875 WGS ID LMAF01 1/10-409
bchN GCA 000715015.1 Ery DSM 6997 v1 protein KEO90155.1 light-independent protochlorophyllide reductase subunit N Erythrobacter longus /8-408
bchN GCA 000152865.1 ASM15286v1 protein EAQ27796.1 light-independent protochlorophyllide reductase subunit N Erythrobacter sp. NAP1 /8-408
bchN GCA 001296025.1 ASM129602v1 protein KPF62021.1 light-independent protochlorophyllide reductase subunit N Porphyrobacter sp. AAP60 /1-401
bchN d Bacteria p Proteobacteria c Alphaproteobacteria o Sphingomonadales f Sphingomonadaceae g Porphyrobacter s Porphyrobacter sanguineus WGS ID MUYH01 1/8-408
bchN GCA 001314765.1 ASM131476v1 protein KPP93893.1 light-independent protochlorophyllide reductase subunit N Erythrobacteraceae bacterium HL-111 /1-399
bchN d Bacteria p Proteobacteria c Alphaproteobacteria o Sphingomonadales f Sphingomonadaceae g Erythrobacter D s Erythrobacter D litoralis WGS ID JMIX01 1/8-408
bchN GCA 000714795.1 Ery DSM 8509 v1 protein KEO92854.1 light-independent protochlorophyllide reductase subunit N Erythrobacter litoralis /8-407
bchN d Bacteria p Proteobacteria c Alphaproteobacteria o Rhizobiales f Beijerinckiaceae g Rhodoblastus s Rhodoblastus acidophilus WGS ID FYDG01 1/1-398
bchN GCA 000019365.1 ASM1936v1 protein ACA18098.1 light-independent protochlorophyllide reductase N subunit Methylobacterium sp. 4-46 /1-399
bchN GCA 001043975.1 ASM104397v1 protein KMO41507.1 light-independent protochlorophyllide reductase subunit N Methylobacterium variabile /1-398
bchN GCA 001043885.1 ASM104388v1 protein KMO20281.1 light-independent protochlorophyllide reductase subunit N Methylobacterium platani JCM 14648 /1-398
bchN GCA 001043955.1 ASM104395v1 protein KMO40292.1 light-independent protochlorophyllide reductase subunit N Methylobacterium tarhaniae /1-398
bchN GCA 001043875.1 ASM104387v1 protein KMO25122.1 light-independent protochlorophyllide reductase subunit N Methylobacterium platani /1-398
bchN GCA 001043915.1 ASM104391v1 protein KMO35939.1 light-independent protochlorophyllide reductase subunit N Methylobacterium aquaticum /1-398
bchN GCA 001043895.1 ASM104389v1 protein KMO21438.1 light-independent protochlorophyllide reductase subunit N Methylobacterium platani /1-398
bchN GCA 000019945.1 ASM1994v1 protein ACB83453.1 light-independent protochlorophyllide reductase N subunit Methylobacterium populi BJ001 /1-398
bchN GCA 000083545.1 ASM8354v1 protein CAX27473.1 protochlorophyllide reductase subunit BchN Methylobacterium extorquens DM4 /9-406
bchN GCA 000022685.1 ASM2268v1 protein ACS42875.1 protochlorophyllide reductase subunit BchN Methylobacterium extorquens AM1 /1-397
bchN GCA 000243435.2 ASM24343v2 protein EHP90537.1 Light-independent protochlorophyllide reductase subunit N Methylobacterium extorquens DSM 13060 /1-396
bchN GCA 000021845.1 ASM2184v1 protein ACK86045.1 light-independent protochlorophyllide reductase N subunit Methylobacterium extorquens CM4 /9-407
bchN GCA 000018845.1 ASM1884v1 protein ABY33184.1 light-independent protochlorophyllide reductase N subunit Methylobacterium extorquens PA1 /9-406
bchN GCA 000272495.1 ASM27249v1 protein EIZ85860.1 light-independent protochlorophyllide reductase subunit N Methylobacterium sp. GXF4 /1-399
bchN GCA 001262595.1 ASM126259v1 protein KNY24603.1 light-independent protochlorophyllide reductase subunit N Methylobacterium sp. ARG-1 /1-399
bchN GCA 000757795.1 ASM75779v1 protein AIQ90007.1 light-independent protochlorophyllide reductase subunit N Methylobacterium oryzae CBMB20 /1-399
bchN GCA 001280155.1 ASM128015v1 protein KOX60796.1 light-independent protochlorophyllide reductase subunit N Streptomyces purpurogeneiscleroticus /1-399
bchN GCA 000364445.1 ASM36444v1 protein EMS40627.1 Light-independent protochlorophyllide reductase subunit N Methylobacterium mesophilicum SR1.6/6 /1-399
bchN GCA 000019725.1 ASM1972v1 protein ACB23843.1 light-independent protochlorophyllide reductase N subunit Methylobacterium radiotolerans JCM 2831 /1-399
bchN GCA 001280145.1 ASM128014v1 protein KOX58059.1 light-independent protochlorophyllide reductase subunit N Asanoa ferruginea /1-399
bchN d Bacteria p Proteobacteria c Alphaproteobacteria o Acetobacterales f Acetobacteraceae g Roseomonas A s Roseomonas A sp003173615 WGS ID QGNA01 1/1-400
bchN d Bacteria p Proteobacteria c Alphaproteobacteria o Acetobacterales f Acetobacteraceae g Ga0074136 s Ga0074136 sp001464375 WGS ID LNEW01 1/6-405
bchN d Bacteria p Proteobacteria c Alphaproteobacteria o Acetobacterales f Acetobacteraceae g Paracraurococcus s Paracraurococcus sp003258945 WGS ID QLIX01 1/1-399
bchN d Bacteria p Proteobacteria c Alphaproteobacteria o Acetobacterales f Acetobacteraceae g Belnapia s Belnapia rosea WGS ID FMZX01 1/19-418
bchN d Bacteria p Proteobacteria c Alphaproteobacteria o Rhizobiales f Phreatobacteraceae g Phreatobacter s Phreatobacter oligotrophus WGS ID PZZL01 1/11-410
bchN d Bacteria p Proteobacteria c Alphaproteobacteria o Rhizobiales f Pleomorphomonadaceae g Mongoliimonas s Mongoliimonas terrestris WGS ID MSIG01 1/1-398
bchN d Bacteria p Proteobacteria c Alphaproteobacteria o Rhizobiales f Pleomorphomonadaceae A g Prosthecomicrobium s Prosthecomicrobium hirschii WGS ID LJYW01 1/1-400
bchN d Bacteria p Proteobacteria c Gammaproteobacteria o Burkholderiales f Burkholderiaceae g Ideonella A s Ideonella A sakaiensis WGS ID BBYR01 1/11-409
bchN d Bacteria p Proteobacteria c Gammaproteobacteria o Burkholderiales f Burkholderiaceae g AAP99 s AAP99 sp001295775 WGS ID LJIA01 1/1-398
bchN GCA 001295775.1 ASM129577v1 protein KPF69242.1 light-independent protochlorophyllide reductase subunit N beta proteobacterium AAP99 /1-399
bchN d Bacteria p Proteobacteria c Alphaproteobacteria o Acetobacterales f Acetobacteraceae g 20-60-12 s 20-60-12 sp002255495 WGS ID NCAP01 1/1-400
bchN GCA 000724705.2 ASM72470v2 protein KDM65824.1 light-independent protochlorophyllide reductase subunit N Acidiphilium sp. JA12-A1 /1-399
bchN d Bacteria p Proteobacteria c Alphaproteobacteria o Acetobacterales f Acetobacteraceae g Acidiphilium s Acidiphilium sp002255515 WGS ID NCAS01 1/1-399
bchN GCA 000202835.1 ASM20283v1 protein BAJ81243.1 light-independent protochlorophyllide reductase subunit N Acidiphilium multivorum AIU301 /1-399
bchN d Bacteria p Proteobacteria c Gammaproteobacteria o Burkholderiales f Burkholderiaceae g Methylibium s Methylibium sp001425905 WGS ID LMDY01 1/6-404
bchN d Bacteria p Proteobacteria c Alphaproteobacteria o Rhizobiales f Beijerinckiaceae g Salinarimonas s Salinarimonas rosea WGS ID AUBC01 1/1-397
bchN d Bacteria p Proteobacteria c Gammaproteobacteria o Burkholderiales f Burkholderiaceae g PHCI01 s PHCI01 sp002842205 WGS ID PHCI01 1/21-420
bchN GCA 001263235.1 ASM126323v1 protein KNZ32949.1 light-independent protochlorophyllide reductase subunit N Methylibium sp. NZG /6-403
bchN d Bacteria p Proteobacteria c Gammaproteobacteria o Burkholderiales f Burkholderiaceae g Rhizobacter s Rhizobacter sp002255905 WGS ID NKIN01 1/1-398
bchN d Bacteria p Proteobacteria c Gammaproteobacteria o Burkholderiales f Burkholderiaceae g BOG-994 s BOG-994 sp003136595 WGS ID PLEY01 1/1-399
bchN d Bacteria p Proteobacteria c Gammaproteobacteria o Burkholderiales f Burkholderiaceae g UBA954 s UBA954 sp002293155 WGS ID DBCQ01 1/1-400
bchN d Bacteria p Proteobacteria c Gammaproteobacteria o Burkholderiales f UBA3065 g UBA3065 s UBA3065 sp002367415 WGS ID DFCB01 1/6-403
bchN GCA 001270065.1 ASM127006v1 protein KOF08573.1 light-independent protochlorophyllide reductase subunit N Limnohabitans planktonicus II-D5 /6-404
bchN d Bacteria p Proteobacteria c Gammaproteobacteria o Burkholderiales f Burkholderiaceae g UBA6679 s UBA6679 sp002454975 WGS ID DKJZ01 1/1-398
bchN d Bacteria p Proteobacteria c Gammaproteobacteria o Burkholderiales f Burkholderiaceae g Roseateles s Roseateles terrae WGS ID NIOG01 1/19-417
bchN d Bacteria p Proteobacteria c Gammaproteobacteria o Burkholderiales f Burkholderiaceae g UBA3064 s UBA3064 sp002359635 WGS ID DENJ01 1/1-397
bchN d Bacteria p Proteobacteria c Gammaproteobacteria o Burkholderiales f Burkholderiaceae g Pseudacidovorax s Pseudacidovorax sp900156165 WGS ID FTMY01 1/1-396
bchN GCA 000015165.1 ASM1516v1 protein ABQ38327.1 protochlorophyllide reductase subunit BchN Bradyrhizobium sp. BTAi1 /12-411
bchN GCA 000344805.1 ASM34480v1 protein BAM91876.1 light-independent protochlorophyllide reductase subunit N Bradyrhizobium oligotrophicum S58 /1-400
bchN GCA 000239775.2 ASM23977v2 protein CCD96368.1 protochlorophyllide reductase subunit BchN Bradyrhizobium sp. ORS 375 /1-400
bchN GCA 000239755.2 ASM23975v2 protein CCD90188.1 protochlorophyllide reductase subunit BchN Bradyrhizobium sp. ORS 285 /1-400
bchN GCA 000026145.1 ASM2614v1 protein CAL75517.1 protochlorophyllide reductase subunit BchN Bradyrhizobium sp. ORS 278 /1-400
bchN GCA 000239795.2 ASM23979v2 protein CCD98110.1 protochlorophyllide reductase subunit BchN Bradyrhizobium sp. STM 3809 /1-400
bchN GCA 000465325.1 Brad ente CordColits V1 protein ERF83829.1 light-independent protochlorophyllide reductase subunit N Bradyrhizobium sp. DFCI-1 /1-399
bchN GCA 000284275.1 ASM28427v1 protein BAL75151.1 protochlorophyllide reductase subunit BchN Bradyrhizobium sp. S23321 /1-399
bchN GCA 000935205.1 ASM93520v1 protein KIZ47041.1 light-independent protochlorophyllide reductase subunit N Rhodopseudomonas palustris /1-398
bchN GCA 000014825.1 ASM1482v1 protein ABJ05299.1 light-independent protochlorophyllide reductase N subunit Rhodopseudomonas palustris BisA53 /1-398
bchN GCA 000013745.1 ASM1374v1 protein ABD86875.1 light-independent protochlorophyllide reductase N subunit Rhodopseudomonas palustris BisB18 /3-402
bchN GCA 000013685.1 ASM1368v1 protein ABE40957.1 light-independent protochlorophyllide reductase N subunit Rhodopseudomonas palustris BisB5 /1-398
bchN GCA 000013365.1 ASM1336v1 protein ABD08674.1 light-independent protochlorophyllide reductase N subunit Rhodopseudomonas palustris HaA2 /1-397
bchN GCA 001295845.1 ASM129584v1 protein KPF92561.1 light-independent protochlorophyllide reductase subunit N Rhodopseudomonas sp. AAP120 /1-398
bchN GCA 000177255.2 ASM17725v2 protein ADU45534.1 light-independent protochlorophyllide reductase N subunit Rhodopseudomonas palustris DX-1 /1-398
bchN d Bacteria p Proteobacteria c Alphaproteobacteria o Rhizobiales f Xanthobacteraceae g Rhodopseudomonas s Rhodopseudomonas palustris I WGS ID QKQS01 1/1-398
bchN GCA 000195775.1 ASM19577v1 protein CAE26983.1 protochlorophyllide reductase subunit BchN Rhodopseudomonas palustris CGA009 /1-398
bchN GCA 000020445.1 ASM2044v1 protein ACF00258.1 light-independent protochlorophyllide reductase N subunit Rhodopseudomonas palustris TIE-1 /1-398
bchN d Bacteria p Proteobacteria c Alphaproteobacteria o Rhizobiales f Beijerinckiaceae g Methylocystis s Methylocystis rosea WGS ID ARCT01 1/1-400
bchN GCA 000016185.1 ASM1618v1 protein ACI99504.1 light-independent protochlorophyllide reductase N subunit Rhodospirillum centenum SW /6-404
bchN d Bacteria p Proteobacteria c Alphaproteobacteria o Azospirillales f Azospirillaceae g Rhodocista s Rhodocista sp001939945 WGS ID MOEB01 1/1-404
bchN GCA 000936425.1 ASM93642v1 protein KJB96108.1 light-independent protochlorophyllide reductase subunit N Skermanella aerolata KACC 11604 /1-396
bchN GCA 000576635.1 genome protein EWY39540.1 light-independent protochlorophyllide reductase subunit N Skermanella stibiiresistens SB22 /1-399
bchN d Bacteria p Proteobacteria c Alphaproteobacteria o Azospirillales f Azospirillaceae g Skermanella s Skermanella stibiiresistens WGS ID AVFL01 1/1-424
bchN d Bacteria p Proteobacteria c Alphaproteobacteria o Rhodospirillales B f Magnetospirillaceae g Phaeospirillum s Phaeospirillum fulvum A WGS ID FNWO01 1/4-402
bchN GCA 000442515.1 PhaespirillumFulvum 1.0 protein EPY03349.1 light-independent protochlorophyllide reductase subunit N Phaeospirillum fulvum MGU-K5 /1-399
bchN GCA 000294655.1 Pm DSM120 protein CCG39600.1 protochlorophyllide reductase subunit BchN Phaeospirillum molischianum DSM 120 /1-399

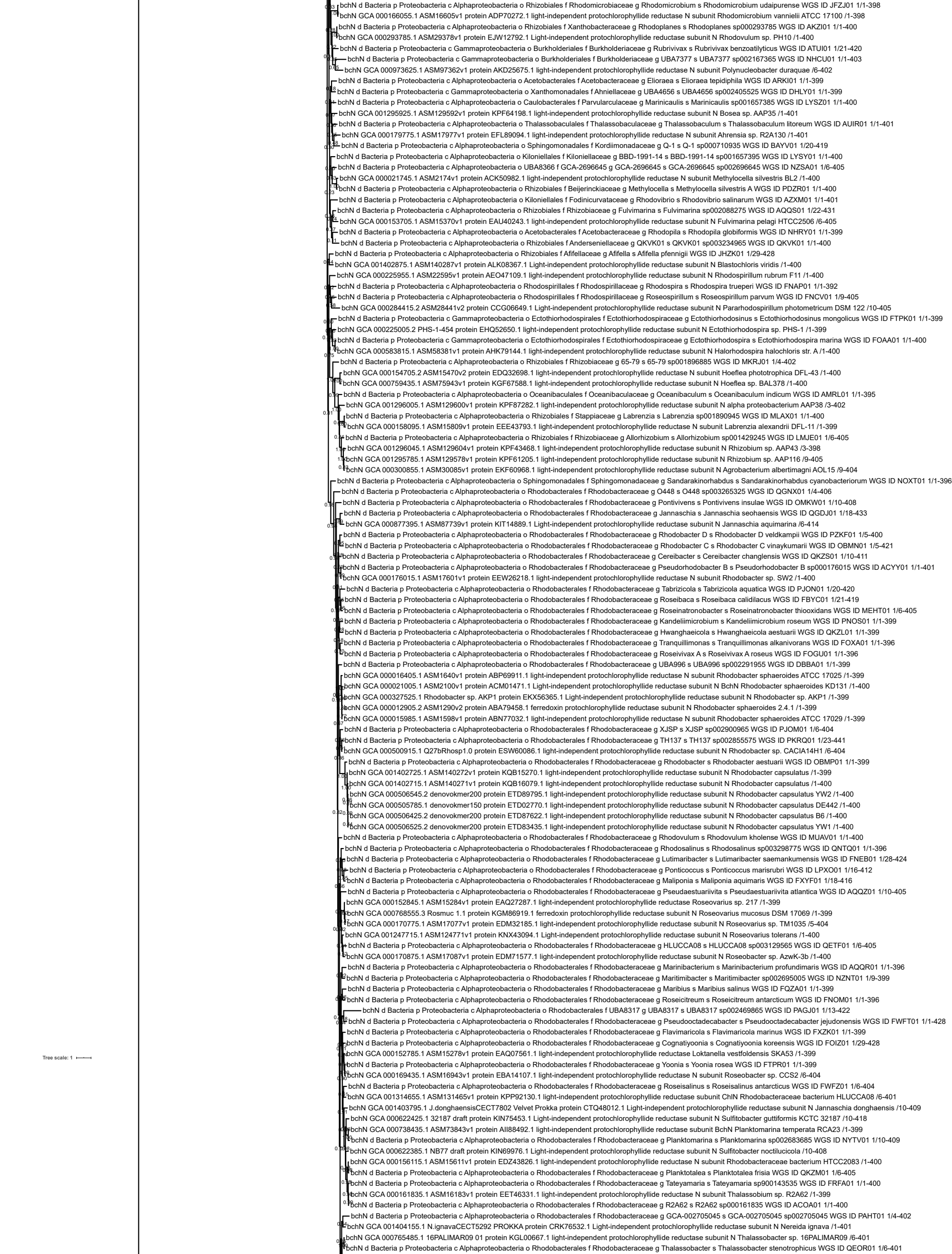
bchN d Bacteria p Proteobacteria c Alphaproteobacteria o Rhizobiales f Rhodomicrobiaceae g Rhodomicrobium s Rhodomicrobium udaipurense WGS ID JFZJ01 1/1-398
bchN GCA 000166055.1 ASM16605v1 protein ADP70272.1 light-independent protochlorophyllide reductase N subunit Rhodomicrobium vannielii ATCC 17100 /1-398
bchN d Bacteria p Proteobacteria c Alphaproteobacteria o Rhizobiales f Xanthobacteraceae g Rhodoplanes s Rhodoplanes sp000293785 WGS ID AKZI01 1/1-400
bchN GCA 000293785.1 ASM29378v1 protein EJW12792.1 Light-independent protochlorophyllide reductase subunit N Rhodovulum sp. PH10 /1-400
bchN d Bacteria p Proteobacteria c Gammaproteobacteria o Burkholderiales f Burkholderiaceae g Rubrivivax s Rubrivivax benzoatilyticus WGS ID ATUI01 1/21-420
bchN d Bacteria p Proteobacteria c Gammaproteobacteria o Burkholderiales f Burkholderiaceae g UBA7377 s UBA7377 sp002167365 WGS ID NHCU01 1/1-403
bchN GCA 000973625.1 ASM97362v1 protein AKD25675.1 light-independent protochlorophyllide reductase N subunit Polynucleobacter duraquae /6-402
bchN d Bacteria p Proteobacteria c Alphaproteobacteria o Acetobacterales f Acetobacteraceae g Elioraea s Elioraea tepidiphila WGS ID ARKI01 1/1-399
bchN d Bacteria p Proteobacteria c Gammaproteobacteria o Xanthomonadales f Ahniellaceae g UBA4656 s UBA4656 sp002405525 WGS ID DHLY01 1/1-399
bchN d Bacteria p Proteobacteria c Alphaproteobacteria o Caulobacterales f Parvularculaceae g Marinicaulis s Marinicaulis sp001657385 WGS ID LYSZ01 1/1-400
bchN GCA 001295925.1 ASM129592v1 protein KPF64198.1 light-independent protochlorophyllide reductase subunit N Bosea sp. AAP35 /1-401
bchN d Bacteria p Proteobacteria c Alphaproteobacteria o Thalassobaculales f Thalassobaculaceae g Thalassobaculum s Thalassobaculum litoreum WGS ID AUIR01 1/1-401
bchN GCA 000179775.1 ASM17977v1 protein EFL89094.1 light-independent protochlorophyllide reductase N subunit Ahrensia sp. R2A130 /1-401
bchN d Bacteria p Proteobacteria c Alphaproteobacteria o Sphingomonadales f Kordiimonadaceae g Q-1 s Q-1 sp000710935 WGS ID BAYV01 1/20-419
bchN d Bacteria p Proteobacteria c Alphaproteobacteria o Kiloniellales f Kiloniellaceae g BBD-1991-14 s BBD-1991-14 sp001657395 WGS ID LYSY01 1/1-400
bchN d Bacteria p Proteobacteria c Alphaproteobacteria o UBA8366 f GCA-2696645 g GCA-2696645 s GCA-2696645 sp002696645 WGS ID NZSA01 1/6-405
bchN GCA 000021745.1 ASM2174v1 protein ACK50982.1 light-independent protochlorophyllide reductase N subunit Methylocella silvestris BL2 /1-400
bchN d Bacteria p Proteobacteria c Alphaproteobacteria o Rhizobiales f Beijerinckiaceae g Methylocella s Methylocella silvestris A WGS ID PDZR01 1/1-400
bchN d Bacteria p Proteobacteria c Alphaproteobacteria o Kiloniellales f Fodinicurvataceae g Rhodovibrio s Rhodovibrio salinarum WGS ID AZXM01 1/1-401
bchN d Bacteria p Proteobacteria c Alphaproteobacteria o Rhizobiales f Rhizobiaceae g Fulvimarina s Fulvimarina sp002088275 WGS ID AQQS01 1/22-431
bchN GCA 000153705.1 ASM15370v1 protein EAU40243.1 light-independent protochlorophyllide reductase subunit N Fulvimarina pelagi HTCC2506 /6-405
bchN d Bacteria p Proteobacteria c Alphaproteobacteria o Acetobacterales f Acetobacteraceae g Rhodopila s Rhodopila globiformis WGS ID NHRY01 1/1-399
bchN d Bacteria p Proteobacteria c Alphaproteobacteria o Rhizobiales f Anderseniellaceae g QKVK01 s QKVK01 sp003234965 WGS ID QKVK01 1/1-400
bchN d Bacteria p Proteobacteria c Alphaproteobacteria o Rhizobiales f Afifellaceae g Afifella s Afifella pfennigii WGS ID JHZK01 1/29-428
bchN GCA 001402875.1 ASM140287v1 protein ALK08367.1 Light-independent protochlorophyllide reductase subunit N Blastochloris viridis /1-400
bchN GCA 000225955.1 ASM22595v1 protein AEO47109.1 light-independent protochlorophyllide reductase subunit N Rhodospirillum rubrum F11 /1-400
bchN d Bacteria p Proteobacteria c Alphaproteobacteria o Rhodospirillales f Rhodospirillaceae g Rhodospira s Rhodospira trueperi WGS ID FNAP01 1/1-392
bchN d Bacteria p Proteobacteria c Alphaproteobacteria o Rhodospirillales f Rhodospirillaceae g Roseospirillum s Roseospirillum parvum WGS ID FNCV01 1/9-405
bchN GCA 000284415.2 ASM28441v2 protein CCG06649.1 Light-independent protochlorophyllide reductase subunit N Pararhodospirillum photometricum DSM 122 /10-405
bchN d Bacteria p Proteobacteria c Gammaproteobacteria o Ectothiorhodospirales f Ectothiorhodospiraceae g Ectothiorhodosinus s Ectothiorhodosinus mongolicus WGS ID FTPK01 1/1-399
bchN GCA 000225005.2 PHS-1-454 protein EHQ52650.1 light-independent protochlorophyllide reductase subunit N Ectothiorhodospira sp. PHS-1 /1-399
bchN d Bacteria p Proteobacteria c Gammaproteobacteria o Ectothiorhodospirales f Ectothiorhodospiraceae g Ectothiorhodospira s Ectothiorhodospira marina WGS ID FOAA01 1/1-400
bchN GCA 000583815.1 ASM58381v1 protein AHK79144.1 light-independent protochlorophyllide reductase subunit N Halorhodospira halochloris str. A /1-400
bchN d Bacteria p Proteobacteria c Alphaproteobacteria o Rhizobiales f Rhizobiaceae g 65-79 s 65-79 sp001896885 WGS ID MKRJ01 1/4-402
bchN GCA 000154705.2 ASM15470v2 protein EDQ32698.1 light-independent protochlorophyllide reductase N subunit Hoeflea phototrophica DFL-43 /1-400
bchN GCA 000759435.1 ASM75943v1 protein KGF67588.1 light-independent protochlorophyllide reductase subunit N Hoeflea sp. BAL378 /1-400
bchN d Bacteria p Proteobacteria c Alphaproteobacteria o Oceanibaculales f Oceanibaculaceae g Oceanibaculum s Oceanibaculum indicum WGS ID AMRL01 1/1-395
bchN GCA 001296005.1 ASM129600v1 protein KPF87282.1 light-independent protochlorophyllide reductase subunit N alpha proteobacterium AAP38 /3-402
bchN d Bacteria p Proteobacteria c Alphaproteobacteria o Rhizobiales f Stappiaceae g Labrenzia s Labrenzia sp001890945 WGS ID MLAX01 1/1-400
bchN GCA 000158095.1 ASM15809v1 protein EEE43793.1 light-independent protochlorophyllide reductase N subunit Labrenzia alexandrii DFL-11 /1-399
bchN d Bacteria p Proteobacteria c Alphaproteobacteria o Rhizobiales f Rhizobiaceae g Allorhizobium s Allorhizobium sp001429245 WGS ID LMJE01 1/6-405
bchN GCA 001296045.1 ASM129604v1 protein KPF43468.1 light-independent protochlorophyllide reductase subunit N Rhizobium sp. AAP43 /3-398
bchN GCA 001295785.1 ASM129578v1 protein KPF61205.1 light-independent protochlorophyllide reductase subunit N Rhizobium sp. AAP116 /9-405
bchN GCA 000300855.1 ASM30085v1 protein EKF60968.1 light-independent protochlorophyllide reductase subunit N Agrobacterium albertimagni AOL15 /9-404
bchN d Bacteria p Proteobacteria c Alphaproteobacteria o Sphingomonadales f Sphingomonadaceae g Sandarakinorhabdus s Sandarakinorhabdus cyanobacteriorum WGS ID NOXT01 1/1-396
bchN d Bacteria p Proteobacteria c Alphaproteobacteria o Rhodobacterales f Rhodobacteraceae g O448 s O448 sp003265325 WGS ID QGNX01 1/4-406
bchN d Bacteria p Proteobacteria c Alphaproteobacteria o Rhodobacterales f Rhodobacteraceae g Pontivivens s Pontivivens insulae WGS ID OMKW01 1/10-408
bchN d Bacteria p Proteobacteria c Alphaproteobacteria o Rhodobacterales f Rhodobacteraceae g Jannaschia s Jannaschia seohaensis WGS ID QGDJ01 1/18-433
bchN GCA 000877395.1 ASM87739v1 protein KIT14889.1 Light-independent protochlorophyllide reductase subunit N Jannaschia aquimarina /6-414
bchN d Bacteria p Proteobacteria c Alphaproteobacteria o Rhodobacterales f Rhodobacteraceae g Rhodobacter D s Rhodobacter D veldkampii WGS ID PZKF01 1/5-400
bchN d Bacteria p Proteobacteria c Alphaproteobacteria o Rhodobacterales f Rhodobacteraceae g Rhodobacter C s Rhodobacter C vinaykumarii WGS ID OBMN01 1/5-421
bchN d Bacteria p Proteobacteria c Alphaproteobacteria o Rhodobacterales f Rhodobacteraceae g Cereibacter s Cereibacter changlensis WGS ID QKZS01 1/10-411
bchN d Bacteria p Proteobacteria c Alphaproteobacteria o Rhodobacterales f Rhodobacteraceae g Pseudorhodobacter B s Pseudorhodobacter B sp000176015 WGS ID ACYY01 1/1-401
bchN GCA 000176015.1 ASM17601v1 protein EEW26218.1 light-independent protochlorophyllide reductase N subunit Rhodobacter sp. SW2 /1-400
bchN d Bacteria p Proteobacteria c Alphaproteobacteria o Rhodobacterales f Rhodobacteraceae g Tabrizicola s Tabrizicola aquatica WGS ID PJON01 1/20-420
bchN d Bacteria p Proteobacteria c Alphaproteobacteria o Rhodobacterales f Rhodobacteraceae g Roseibaca s Roseibaca calidilacus WGS ID FBYC01 1/21-419
bchN d Bacteria p Proteobacteria c Alphaproteobacteria o Rhodobacterales f Rhodobacteraceae g Roseinatronobacter s Roseinatronobacter thiooxidans WGS ID MEHT01 1/6-405
bchN d Bacteria p Proteobacteria c Alphaproteobacteria o Rhodobacterales f Rhodobacteraceae g Kandeliimicrobium s Kandeliimicrobium roseum WGS ID PNOS01 1/1-399
bchN d Bacteria p Proteobacteria c Alphaproteobacteria o Rhodobacterales f Rhodobacteraceae g Hwanghaeicola s Hwanghaeicola aestuarii WGS ID QKZL01 1/1-399
bchN d Bacteria p Proteobacteria c Alphaproteobacteria o Rhodobacterales f Rhodobacteraceae g Tranquillimonas s Tranquillimonas alkanivorans WGS ID FOXA01 1/1-396
bchN d Bacteria p Proteobacteria c Alphaproteobacteria o Rhodobacterales f Rhodobacteraceae g Roseivivax A s Roseivivax A roseus WGS ID FOGU01 1/1-396
bchN d Bacteria p Proteobacteria c Alphaproteobacteria o Rhodobacterales f Rhodobacteraceae g UBA996 s UBA996 sp002291955 WGS ID DBBA01 1/1-399
bchN GCA 000016405.1 ASM1640v1 protein ABP69911.1 light-independent protochlorophyllide reductase N subunit Rhodobacter sphaeroides ATCC 17025 /1-399
bchN GCA 000021005.1 ASM2100v1 protein ACM01471.1 Light-independent protochlorophyllide reductase subunit N BchN Rhodobacter sphaeroides KD131 /1-400
bchN GCA 000327525.1 Rhodobacter sp. AKP1 protein EKX56365.1 Light-independent protochlorophyllide reductase subunit N Rhodobacter sp. AKP1 /1-399
bchN GCA 000012905.2 ASM1290v2 protein ABA79458.1 ferredoxin protochlorophyllide reductase subunit N Rhodobacter sphaeroides 2.4.1 /1-399
bchN GCA 000015985.1 ASM1598v1 protein ABN77032.1 light-independent protochlorophyllide reductase N subunit Rhodobacter sphaeroides ATCC 17029 /1-399
bchN d Bacteria p Proteobacteria c Alphaproteobacteria o Rhodobacterales f Rhodobacteraceae g XJSP s XJSP sp002900965 WGS ID PJOM01 1/6-404
bchN d Bacteria p Proteobacteria c Alphaproteobacteria o Rhodobacterales f Rhodobacteraceae g TH137 s TH137 sp002855575 WGS ID PKRQ01 1/23-441
bchN GCA 000500915.1 Q27bRhosp1.0 protein ESW60086.1 light-independent protochlorophyllide reductase subunit N Rhodobacter sp. CACIA14H1 /6-404
bchN d Bacteria p Proteobacteria c Alphaproteobacteria o Rhodobacterales f Rhodobacteraceae g Rhodobacter s Rhodobacter aestuarii WGS ID OBMP01 1/1-399
bchN GCA 001402725.1 ASM140272v1 protein KQB15270.1 light-independent protochlorophyllide reductase subunit N Rhodobacter capsulatus /1-399
bchN GCA 001402715.1 ASM140271v1 protein KQB16079.1 light-independent protochlorophyllide reductase subunit N Rhodobacter capsulatus /1-400
bchN GCA 000506545.2 denovokmer200 protein ETD89795.1 light-independent protochlorophyllide reductase subunit N Rhodobacter capsulatus YW2 /1-400
bchN GCA 000505785.1 denovokmer150 protein ETD02770.1 light-independent protochlorophyllide reductase subunit N Rhodobacter capsulatus DE442 /1-400
bchN GCA 000506425.2 denovokmer200 protein ETD87622.1 light-independent protochlorophyllide reductase subunit N Rhodobacter capsulatus B6 /1-400
bchN GCA 000506525.2 denovokmer200 protein ETD83435.1 light-independent protochlorophyllide reductase subunit N Rhodobacter capsulatus YW1 /1-400
bchN d Bacteria p Proteobacteria c Alphaproteobacteria o Rhodobacterales f Rhodobacteraceae g Rhodovulum s Rhodovulum kholense WGS ID MUAV01 1/1-400
bchN d Bacteria p Proteobacteria c Alphaproteobacteria o Rhodobacterales f Rhodobacteraceae g Rhodosalinus s Rhodosalinus sp003298775 WGS ID QNTQ01 1/1-396
bchN d Bacteria p Proteobacteria c Alphaproteobacteria o Rhodobacterales f Rhodobacteraceae g Lutimaribacter s Lutimaribacter saemankumensis WGS ID FNEB01 1/28-424
bchN d Bacteria p Proteobacteria c Alphaproteobacteria o Rhodobacterales f Rhodobacteraceae g Ponticoccus s Ponticoccus marisrubri WGS ID LPXO01 1/16-412
bchN d Bacteria p Proteobacteria c Alphaproteobacteria o Rhodobacterales f Rhodobacteraceae g Maliponia s Maliponia aquimaris WGS ID FXYF01 1/18-416
bchN d Bacteria p Proteobacteria c Alphaproteobacteria o Rhodobacterales f Rhodobacteraceae g Pseudaestuariivita s Pseudaestuariivita atlantica WGS ID AQQZ01 1/10-405
bchN GCA 000152845.1 ASM15284v1 protein EAQ27287.1 light-independent protochlorophyllide reductase Roseovarius sp. 217 /1-399
bchN GCA 000768555.3 Rosmuc 1.1 protein KGM86919.1 ferredoxin protochlorophyllide reductase subunit N Roseovarius mucosus DSM 17069 /1-399
bchN GCA 000170775.1 ASM17077v1 protein EDM32185.1 light-independent protochlorophyllide reductase subunit N Roseovarius sp. TM1035 /5-404
bchN GCA 001247715.1 ASM124771v1 protein KNX43094.1 Light-independent protochlorophyllide reductase subunit N Roseovarius tolerans /1-400
bchN d Bacteria p Proteobacteria c Alphaproteobacteria o Rhodobacterales f Rhodobacteraceae g HLUCCA08 s HLUCCA08 sp003129565 WGS ID QETF01 1/6-405
bchN GCA 000170875.1 ASM17087v1 protein EDM71577.1 light-independent protochlorophyllide reductase subunit N Roseobacter sp. AzwK-3b /1-400
bchN d Bacteria p Proteobacteria c Alphaproteobacteria o Rhodobacterales f Rhodobacteraceae g Marinibacterium s Marinibacterium profundimaris WGS ID AQQR01 1/1-396
bchN d Bacteria p Proteobacteria c Alphaproteobacteria o Rhodobacterales f Rhodobacteraceae g Maritimibacter s Maritimibacter sp002695005 WGS ID NZNT01 1/9-399
bchN d Bacteria p Proteobacteria c Alphaproteobacteria o Rhodobacterales f Rhodobacteraceae g Maribius s Maribius salinus WGS ID FQZA01 1/1-399
bchN d Bacteria p Proteobacteria c Alphaproteobacteria o Rhodobacterales f Rhodobacteraceae g Roseicitreum s Roseicitreum antarcticum WGS ID FNOM01 1/1-396
bchN d Bacteria p Proteobacteria c Alphaproteobacteria o Rhodobacterales f UBA8317 g UBA8317 s UBA8317 sp002469865 WGS ID PAGJ01 1/13-422
bchN d Bacteria p Proteobacteria c Alphaproteobacteria o Rhodobacterales f Rhodobacteraceae g Pseudooctadecabacter s Pseudooctadecabacter jejudonensis WGS ID FWFT01 1/1-428
bchN d Bacteria p Proteobacteria c Alphaproteobacteria o Rhodobacterales f Rhodobacteraceae g Flavimaricola s Flavimaricola marinus WGS ID FXZK01 1/1-399
bchN d Bacteria p Proteobacteria c Alphaproteobacteria o Rhodobacterales f Rhodobacteraceae g Cognatiyoonia s Cognatiyoonia koreensis WGS ID FOIZ01 1/29-428
bchN GCA 000152785.1 ASM15278v1 protein EAQ07561.1 light-independent protochlorophyllide reductase Loktanella vestfoldensis SKA53 /1-399
bchN d Bacteria p Proteobacteria c Alphaproteobacteria o Rhodobacterales f Rhodobacteraceae g Yoonia s Yoonia rosea WGS ID FTPR01 1/1-399
bchN GCA 000169435.1 ASM16943v1 protein EBA14107.1 light-independent protochlorophyllide reductase N subunit Roseobacter sp. CCS2 /6-404
bchN d Bacteria p Proteobacteria c Alphaproteobacteria o Rhodobacterales f Rhodobacteraceae g Roseisalinus s Roseisalinus antarcticus WGS ID FWFZ01 1/6-404
bchN GCA 001314655.1 ASM131465v1 protein KPP92130.1 light-independent protochlorophyllide reductase subunit ChlN Rhodobacteraceae bacterium HLUCCA08 /6-401
bchN GCA 001403795.1 J.donghaensisCECT7802 Velvet Prokka protein CTQ48012.1 Light-independent protochlorophyllide reductase subunit N Jannaschia donghaensis /10-409
bchN GCA 000622425.1 32187 draft protein KIN75453.1 Light-independent protochlorophyllide reductase subunit N Sulfitobacter guttiformis KCTC 32187 /10-418
bchN GCA 000738435.1 ASM73843v1 protein AII88492.1 light-independent protochlorophyllide reductase subunit BchN Planktomarina temperata RCA23 /1-399
bchN d Bacteria p Proteobacteria c Alphaproteobacteria o Rhodobacterales f Rhodobacteraceae g Planktomarina s Planktomarina sp002683685 WGS ID NYTV01 1/10-409
bchN GCA 000622385.1 NB77 draft protein KIN69976.1 Light-independent protochlorophyllide reductase subunit N Sulfitobacter noctilucicola /10-408
bchN GCA 000156115.1 ASM15611v1 protein EDZ43826.1 light-independent protochlorophyllide reductase N subunit Rhodobacteraceae bacterium HTCC2083 /1-400
bchN d Bacteria p Proteobacteria c Alphaproteobacteria o Rhodobacterales f Rhodobacteraceae g Planktotalea s Planktotalea frisia WGS ID QKZM01 1/6-405
bchN d Bacteria p Proteobacteria c Alphaproteobacteria o Rhodobacterales f Rhodobacteraceae g Tateyamaria s Tateyamaria sp900143535 WGS ID FRFA01 1/1-400
bchN GCA 000161835.1 ASM16183v1 protein EET46331.1 light-independent protochlorophyllide reductase subunit N Thalassobium sp. R2A62 /1-399
bchN d Bacteria p Proteobacteria c Alphaproteobacteria o Rhodobacterales f Rhodobacteraceae g R2A62 s R2A62 sp000161835 WGS ID ACOA01 1/1-400
bchN d Bacteria p Proteobacteria c Alphaproteobacteria o Rhodobacterales f Rhodobacteraceae g GCA-002705045 s GCA-002705045 sp002705045 WGS ID PAHT01 1/4-402
bchN GCA 001404155.1 N.ignavaCECT5292 PROKKA protein CRK76532.1 Light-independent protochlorophyllide reductase subunit N Nereida ignava /1-401
bchN GCA 000765485.1 16PALIMAR09 01 protein KGL00667.1 light-independent protochlorophyllide reductase subunit N Thalassobacter sp. 16PALIMAR09 /6-401
bchN d Bacteria p Proteobacteria c Alphaproteobacteria o Rhodobacterales f Rhodobacteraceae g Thalassobacter s Thalassobacter stenotrophicus WGS ID QEOR01 1/6-401
Tree scale: 1

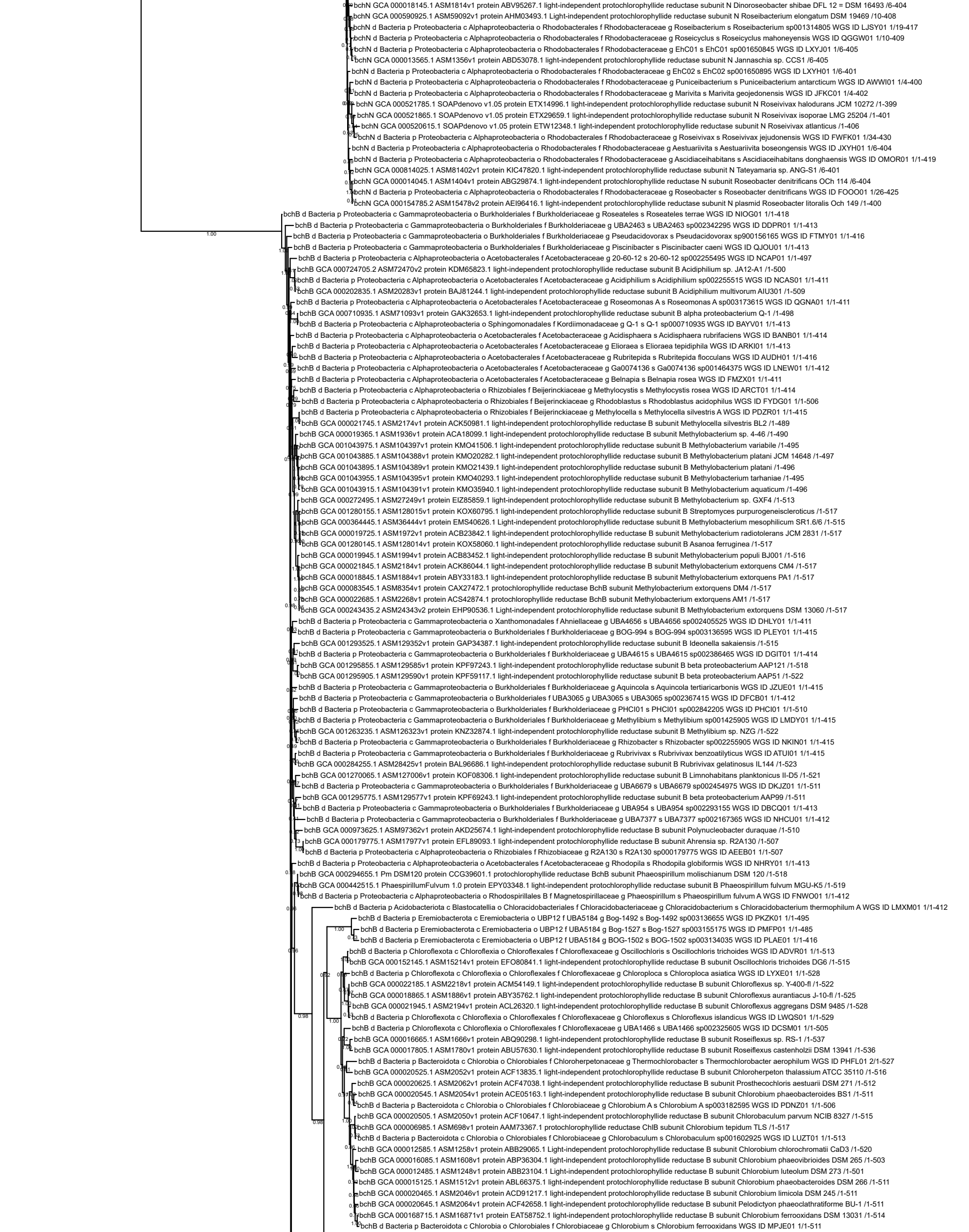
bchN GCA 000018145.1 ASM1814v1 protein ABV95267.1 light-independent protochlorophyllide reductase subunit N Dinoroseobacter shibae DFL 12 = DSM 16493 /6-404
bchN GCA 000590925.1 ASM59092v1 protein AHM03493.1 Light-independent protochlorophyllide reductase subunit N Roseibacterium elongatum DSM 19469 /10-408
bchN d Bacteria p Proteobacteria c Alphaproteobacteria o Rhodobacterales f Rhodobacteraceae g Roseibacterium s Roseibacterium sp001314805 WGS ID LJSY01 1/19-417
bchN d Bacteria p Proteobacteria c Alphaproteobacteria o Rhodobacterales f Rhodobacteraceae g Roseicyclus s Roseicyclus mahoneyensis WGS ID QGGW01 1/10-409
bchN d Bacteria p Proteobacteria c Alphaproteobacteria o Rhodobacterales f Rhodobacteraceae g EhC01 s EhC01 sp001650845 WGS ID LXYJ01 1/6-405
bchN GCA 000013565.1 ASM1356v1 protein ABD53078.1 light-independent protochlorophyllide reductase subunit N Jannaschia sp. CCS1 /6-405
bchN d Bacteria p Proteobacteria c Alphaproteobacteria o Rhodobacterales f Rhodobacteraceae g EhC02 s EhC02 sp001650895 WGS ID LXYH01 1/6-401
bchN d Bacteria p Proteobacteria c Alphaproteobacteria o Rhodobacterales f Rhodobacteraceae g Puniceibacterium s Puniceibacterium antarcticum WGS ID AWWI01 1/4-400
bchN d Bacteria p Proteobacteria c Alphaproteobacteria o Rhodobacterales f Rhodobacteraceae g Marivita s Marivita geojedonensis WGS ID JFKC01 1/4-402
bchN GCA 000521785.1 SOAPdenovo v1.05 protein ETX14996.1 light-independent protochlorophyllide reductase subunit N Roseivivax halodurans JCM 10272 /1-399
bchN GCA 000521865.1 SOAPdenovo v1.05 protein ETX29659.1 light-independent protochlorophyllide reductase subunit N Roseivivax isoporae LMG 25204 /1-401
bchN GCA 000520615.1 SOAPdenovo v1.05 protein ETW12348.1 light-independent protochlorophyllide reductase subunit N Roseivivax atlanticus /1-406
bchN d Bacteria p Proteobacteria c Alphaproteobacteria o Rhodobacterales f Rhodobacteraceae g Roseivivax s Roseivivax jejudonensis WGS ID FWFK01 1/34-430
bchN d Bacteria p Proteobacteria c Alphaproteobacteria o Rhodobacterales f Rhodobacteraceae g Aestuariivita s Aestuariivita boseongensis WGS ID JXYH01 1/6-404
bchN d Bacteria p Proteobacteria c Alphaproteobacteria o Rhodobacterales f Rhodobacteraceae g Ascidiaceihabitans s Ascidiaceihabitans donghaensis WGS ID OMOR01 1/1-419
bchN GCA 000814025.1 ASM81402v1 protein KIC47820.1 light-independent protochlorophyllide reductase subunit N Tateyamaria sp. ANG-S1 /6-401
bchN GCA 000014045.1 ASM1404v1 protein ABG29874.1 light-independent protochlorophyllide reductase N subunit Roseobacter denitrificans OCh 114 /6-404
bchN d Bacteria p Proteobacteria c Alphaproteobacteria o Rhodobacterales f Rhodobacteraceae g Roseobacter s Roseobacter denitrificans WGS ID FOOO01 1/26-425
bchN GCA 000154785.2 ASM15478v2 protein AEI96416.1 light-independent protochlorophyllide reductase subunit N plasmid Roseobacter litoralis Och 149 /1-400
1.00
bchB d Bacteria p Proteobacteria c Gammaproteobacteria o Burkholderiales f Burkholderiaceae g Roseateles s Roseateles terrae WGS ID NIOG01 1/1-418
bchB d Bacteria p Proteobacteria c Gammaproteobacteria o Burkholderiales f Burkholderiaceae g UBA2463 s UBA2463 sp002342295 WGS ID DDPR01 1/1-413
bchB d Bacteria p Proteobacteria c Gammaproteobacteria o Burkholderiales f Burkholderiaceae g Pseudacidovorax s Pseudacidovorax sp900156165 WGS ID FTMY01 1/1-416
bchB d Bacteria p Proteobacteria c Gammaproteobacteria o Burkholderiales f Burkholderiaceae g Piscinibacter s Piscinibacter caeni WGS ID QJOU01 1/1-413
bchB d Bacteria p Proteobacteria c Alphaproteobacteria o Acetobacterales f Acetobacteraceae g 20-60-12 s 20-60-12 sp002255495 WGS ID NCAP01 1/1-497
bchB GCA 000724705.2 ASM72470v2 protein KDM65823.1 light-independent protochlorophyllide reductase subunit B Acidiphilium sp. JA12-A1 /1-500
bchB d Bacteria p Proteobacteria c Alphaproteobacteria o Acetobacterales f Acetobacteraceae g Acidiphilium s Acidiphilium sp002255515 WGS ID NCAS01 1/1-411
bchB GCA 000202835.1 ASM20283v1 protein BAJ81244.1 light-independent protochlorophyllide reductase subunit B Acidiphilium multivorum AIU301 /1-509
bchB d Bacteria p Proteobacteria c Alphaproteobacteria o Acetobacterales f Acetobacteraceae g Roseomonas A s Roseomonas A sp003173615 WGS ID QGNA01 1/1-411
bchB GCA 000710935.1 ASM71093v1 protein GAK32653.1 light-independent protochlorophyllide reductase subunit B alpha proteobacterium Q-1 /1-498
bchB d Bacteria p Proteobacteria c Alphaproteobacteria o Sphingomonadales f Kordiimonadaceae g Q-1 s Q-1 sp000710935 WGS ID BAYV01 1/1-413
bchB d Bacteria p Proteobacteria c Alphaproteobacteria o Acetobacterales f Acetobacteraceae g Acidisphaera s Acidisphaera rubrifaciens WGS ID BANB01 1/1-414
bchB d Bacteria p Proteobacteria c Alphaproteobacteria o Acetobacterales f Acetobacteraceae g Elioraea s Elioraea tepidiphila WGS ID ARKI01 1/1-413
bchB d Bacteria p Proteobacteria c Alphaproteobacteria o Acetobacterales f Acetobacteraceae g Rubritepida s Rubritepida flocculans WGS ID AUDH01 1/1-416
bchB d Bacteria p Proteobacteria c Alphaproteobacteria o Acetobacterales f Acetobacteraceae g Ga0074136 s Ga0074136 sp001464375 WGS ID LNEW01 1/1-412
bchB d Bacteria p Proteobacteria c Alphaproteobacteria o Acetobacterales f Acetobacteraceae g Belnapia s Belnapia rosea WGS ID FMZX01 1/1-411
bchB d Bacteria p Proteobacteria c Alphaproteobacteria o Rhizobiales f Beijerinckiaceae g Methylocystis s Methylocystis rosea WGS ID ARCT01 1/1-414
bchB d Bacteria p Proteobacteria c Alphaproteobacteria o Rhizobiales f Beijerinckiaceae g Rhodoblastus s Rhodoblastus acidophilus WGS ID FYDG01 1/1-506
bchB d Bacteria p Proteobacteria c Alphaproteobacteria o Rhizobiales f Beijerinckiaceae g Methylocella s Methylocella silvestris A WGS ID PDZR01 1/1-415
bchB GCA 000021745.1 ASM2174v1 protein ACK50981.1 light-independent protochlorophyllide reductase B subunit Methylocella silvestris BL2 /1-489
bchB GCA 000019365.1 ASM1936v1 protein ACA18099.1 light-independent protochlorophyllide reductase B subunit Methylobacterium sp. 4-46 /1-490
bchB GCA 001043975.1 ASM104397v1 protein KMO41506.1 light-independent protochlorophyllide reductase subunit B Methylobacterium variabile /1-495
bchB GCA 001043885.1 ASM104388v1 protein KMO20282.1 light-independent protochlorophyllide reductase subunit B Methylobacterium platani JCM 14648 /1-497
bchB GCA 001043895.1 ASM104389v1 protein KMO21439.1 light-independent protochlorophyllide reductase subunit B Methylobacterium platani /1-496
bchB GCA 001043955.1 ASM104395v1 protein KMO40293.1 light-independent protochlorophyllide reductase subunit B Methylobacterium tarhaniae /1-495
bchB GCA 001043915.1 ASM104391v1 protein KMO35940.1 light-independent protochlorophyllide reductase subunit B Methylobacterium aquaticum /1-496
bchB GCA 000272495.1 ASM27249v1 protein EIZ85859.1 light-independent protochlorophyllide reductase subunit B Methylobacterium sp. GXF4 /1-513
bchB GCA 001280155.1 ASM128015v1 protein KOX60795.1 light-independent protochlorophyllide reductase subunit B Streptomyces purpurogeneiscleroticus /1-517
bchB GCA 000364445.1 ASM36444v1 protein EMS40626.1 Light-independent protochlorophyllide reductase subunit B Methylobacterium mesophilicum SR1.6/6 /1-515
bchB GCA 000019725.1 ASM1972v1 protein ACB23842.1 light-independent protochlorophyllide reductase B subunit Methylobacterium radiotolerans JCM 2831 /1-517
bchB GCA 001280145.1 ASM128014v1 protein KOX58060.1 light-independent protochlorophyllide reductase subunit B Asanoa ferruginea /1-517
bchB GCA 000019945.1 ASM1994v1 protein ACB83452.1 light-independent protochlorophyllide reductase B subunit Methylobacterium populi BJ001 /1-516
bchB GCA 000021845.1 ASM2184v1 protein ACK86044.1 light-independent protochlorophyllide reductase B subunit Methylobacterium extorquens CM4 /1-517
bchB GCA 000018845.1 ASM1884v1 protein ABY33183.1 light-independent protochlorophyllide reductase B subunit Methylobacterium extorquens PA1 /1-517
bchB GCA 000083545.1 ASM8354v1 protein CAX27472.1 protochlorophyllide reductase BchB subunit Methylobacterium extorquens DM4 /1-517
bchB GCA 000022685.1 ASM2268v1 protein ACS42874.1 protochlorophyllide reductase BchB subunit Methylobacterium extorquens AM1 /1-517
bchB GCA 000243435.2 ASM24343v2 protein EHP90536.1 Light-independent protochlorophyllide reductase subunit B Methylobacterium extorquens DSM 13060 /1-517
bchB d Bacteria p Proteobacteria c Gammaproteobacteria o Xanthomonadales f Ahniellaceae g UBA4656 s UBA4656 sp002405525 WGS ID DHLY01 1/1-411
bchB d Bacteria p Proteobacteria c Gammaproteobacteria o Burkholderiales f Burkholderiaceae g BOG-994 s BOG-994 sp003136595 WGS ID PLEY01 1/1-415
bchB GCA 001293525.1 ASM129352v1 protein GAP34387.1 light-independent protochlorophyllide reductase subunit B Ideonella sakaiensis /1-515
bchB d Bacteria p Proteobacteria c Gammaproteobacteria o Burkholderiales f Burkholderiaceae g UBA4615 s UBA4615 sp002386465 WGS ID DGIT01 1/1-414
bchB GCA 001295855.1 ASM129585v1 protein KPF97243.1 light-independent protochlorophyllide reductase subunit B beta proteobacterium AAP121 /1-518
bchB GCA 001295905.1 ASM129590v1 protein KPF59117.1 light-independent protochlorophyllide reductase subunit B beta proteobacterium AAP51 /1-522
bchB d Bacteria p Proteobacteria c Gammaproteobacteria o Burkholderiales f Burkholderiaceae g Aquincola s Aquincola tertiaricarbonis WGS ID JZUE01 1/1-415
bchB d Bacteria p Proteobacteria c Gammaproteobacteria o Burkholderiales f UBA3065 g UBA3065 s UBA3065 sp002367415 WGS ID DFCB01 1/1-412
bchB d Bacteria p Proteobacteria c Gammaproteobacteria o Burkholderiales f Burkholderiaceae g PHCI01 s PHCI01 sp002842205 WGS ID PHCI01 1/1-510
bchB d Bacteria p Proteobacteria c Gammaproteobacteria o Burkholderiales f Burkholderiaceae g Methylibium s Methylibium sp001425905 WGS ID LMDY01 1/1-415
bchB GCA 001263235.1 ASM126323v1 protein KNZ32874.1 light-independent protochlorophyllide reductase subunit B Methylibium sp. NZG /1-522
bchB d Bacteria p Proteobacteria c Gammaproteobacteria o Burkholderiales f Burkholderiaceae g Rhizobacter s Rhizobacter sp002255905 WGS ID NKIN01 1/1-415
bchB d Bacteria p Proteobacteria c Gammaproteobacteria o Burkholderiales f Burkholderiaceae g Rubrivivax s Rubrivivax benzoatilyticus WGS ID ATUI01 1/1-415
bchB GCA 000284255.1 ASM28425v1 protein BAL96686.1 light-independent protochlorophyllide reductase subunit B Rubrivivax gelatinosus IL144 /1-523
bchB GCA 001270065.1 ASM127006v1 protein KOF08306.1 light-independent protochlorophyllide reductase subunit B Limnohabitans planktonicus II-D5 /1-521
bchB d Bacteria p Proteobacteria c Gammaproteobacteria o Burkholderiales f Burkholderiaceae g UBA6679 s UBA6679 sp002454975 WGS ID DKJZ01 1/1-511
bchB GCA 001295775.1 ASM129577v1 protein KPF69243.1 light-independent protochlorophyllide reductase subunit B beta proteobacterium AAP99 /1-511
bchB d Bacteria p Proteobacteria c Gammaproteobacteria o Burkholderiales f Burkholderiaceae g UBA954 s UBA954 sp002293155 WGS ID DBCQ01 1/1-413
bchB d Bacteria p Proteobacteria c Gammaproteobacteria o Burkholderiales f Burkholderiaceae g UBA7377 s UBA7377 sp002167365 WGS ID NHCU01 1/1-412
bchB GCA 000973625.1 ASM97362v1 protein AKD25674.1 light-independent protochlorophyllide reductase subunit B Polynucleobacter duraquae /1-510
bchB GCA 000179775.1 ASM17977v1 protein EFL89093.1 light-independent protochlorophyllide reductase B subunit Ahrensia sp. R2A130 /1-507
bchB d Bacteria p Proteobacteria c Alphaproteobacteria o Rhizobiales f Rhizobiaceae g R2A130 s R2A130 sp000179775 WGS ID AEEB01 1/1-507
bchB d Bacteria p Proteobacteria c Alphaproteobacteria o Acetobacterales f Acetobacteraceae g Rhodopila s Rhodopila globiformis WGS ID NHRY01 1/1-413
bchB GCA 000294655.1 Pm DSM120 protein CCG39601.1 protochlorophyllide reductase BchB subunit Phaeospirillum molischianum DSM 120 /1-518
bchB GCA 000442515.1 PhaespirillumFulvum 1.0 protein EPY03348.1 light-independent protochlorophyllide reductase subunit B Phaeospirillum fulvum MGU-K5 /1-519
bchB d Bacteria p Proteobacteria c Alphaproteobacteria o Rhodospirillales B f Magnetospirillaceae g Phaeospirillum s Phaeospirillum fulvum A WGS ID FNWO01 1/1-412
bchB d Bacteria p Acidobacteriota c Blastocatellia o Chloracidobacteriales f Chloracidobacteriaceae g Chloracidobacterium s Chloracidobacterium thermophilum A WGS ID LMXM01 1/1-412
bchB d Bacteria p Eremiobacterota c Eremiobacteria o UBP12 f UBA5184 g Bog-1492 s Bog-1492 sp003136655 WGS ID PKZK01 1/1-495
1.00
bchB d Bacteria p Eremiobacterota c Eremiobacteria o UBP12 f UBA5184 g Bog-1527 s Bog-1527 sp003155175 WGS ID PMFP01 1/1-485
bchB d Bacteria p Eremiobacterota c Eremiobacteria o UBP12 f UBA5184 g BOG-1502 s BOG-1502 sp003134035 WGS ID PLAE01 1/1-416
bchB d Bacteria p Chloroflexota c Chloroflexia o Chloroflexales f Chloroflexaceae g Oscillochloris s Oscillochloris trichoides WGS ID ADVR01 1/1-513
bchB GCA 000152145.1 ASM15214v1 protein EFO80841.1 light-independent protochlorophyllide reductase B subunit Oscillochloris trichoides DG6 /1-515
bchB d Bacteria p Chloroflexota c Chloroflexia o Chloroflexales f Chloroflexaceae g Chloroploca s Chloroploca asiatica WGS ID LYXE01 1/1-528
bchB GCA 000022185.1 ASM2218v1 protein ACM54149.1 light-independent protochlorophyllide reductase B subunit Chloroflexus sp. Y-400-fl /1-522
bchB GCA 000018865.1 ASM1886v1 protein ABY35762.1 light-independent protochlorophyllide reductase B subunit Chloroflexus aurantiacus J-10-fl /1-525
bchB GCA 000021945.1 ASM2194v1 protein ACL26320.1 light-independent protochlorophyllide reductase B subunit Chloroflexus aggregans DSM 9485 /1-528
bchB d Bacteria p Chloroflexota c Chloroflexia o Chloroflexales f Chloroflexaceae g Chloroflexus s Chloroflexus islandicus WGS ID LWQS01 1/1-529
0.98
1.00
bchB d Bacteria p Chloroflexota c Chloroflexia o Chloroflexales f Chloroflexaceae g UBA1466 s UBA1466 sp002325605 WGS ID DCSM01 1/1-505
bchB GCA 000016665.1 ASM1666v1 protein ABQ90298.1 light-independent protochlorophyllide reductase B subunit Roseiflexus sp. RS-1 /1-537
bchB GCA 000017805.1 ASM1780v1 protein ABU57630.1 light-independent protochlorophyllide reductase B subunit Roseiflexus castenholzii DSM 13941 /1-536
bchB d Bacteria p Bacteroidota c Chlorobia o Chlorobiales f Chloroherpetonaceae g Thermochlorobacter s Thermochlorobacter aerophilum WGS ID PHFL01 2/1-527
bchB GCA 000020525.1 ASM2052v1 protein ACF13835.1 light-independent protochlorophyllide reductase B subunit Chloroherpeton thalassium ATCC 35110 /1-516
bchB GCA 000020625.1 ASM2062v1 protein ACF47038.1 light-independent protochlorophyllide reductase B subunit Prosthecochloris aestuarii DSM 271 /1-512
bchB GCA 000020545.1 ASM2054v1 protein ACE05163.1 light-independent protochlorophyllide reductase B subunit Chlorobium phaeobacteroides BS1 /1-511
bchB d Bacteria p Bacteroidota c Chlorobia o Chlorobiales f Chlorobiaceae g Chlorobium A s Chlorobium A sp003182595 WGS ID PDNZ01 1/1-506
0.98
bchB GCA 000020505.1 ASM2050v1 protein ACF10647.1 light-independent protochlorophyllide reductase B subunit Chlorobaculum parvum NCIB 8327 /1-515
bchB GCA 000006985.1 ASM698v1 protein AAM73367.1 protochlorophyllide reductase ChlB subunit Chlorobium tepidum TLS /1-517
bchB d Bacteria p Bacteroidota c Chlorobia o Chlorobiales f Chlorobiaceae g Chlorobaculum s Chlorobaculum sp001602925 WGS ID LUZT01 1/1-513
bchB GCA 000012585.1 ASM1258v1 protein ABB29065.1 light-independent protochlorophyllide reductase B subunit Chlorobium chlorochromatii CaD3 /1-520
bchB GCA 000016085.1 ASM1608v1 protein ABP36304.1 light-independent protochlorophyllide reductase B subunit Chlorobium phaeovibrioides DSM 265 /1-503
bchB GCA 000012485.1 ASM1248v1 protein ABB23104.1 Light-independent protochlorophyllide reductase B subunit Chlorobium luteolum DSM 273 /1-501
bchB GCA 000015125.1 ASM1512v1 protein ABL66375.1 light-independent protochlorophyllide reductase B subunit Chlorobium phaeobacteroides DSM 266 /1-511
bchB GCA 000020465.1 ASM2046v1 protein ACD91217.1 light-independent protochlorophyllide reductase B subunit Chlorobium limicola DSM 245 /1-511
bchB GCA 000020645.1 ASM2064v1 protein ACF42658.1 light-independent protochlorophyllide reductase B subunit Pelodictyon phaeoclathratiforme BU-1 /1-511
bchB GCA 000168715.1 ASM16871v1 protein EAT58752.1 light-independent protochlorophyllide reductase B subunit Chlorobium ferrooxidans DSM 13031 /1-514
bchB d Bacteria p Bacteroidota c Chlorobia o Chlorobiales f Chlorobiaceae g Chlorobium s Chlorobium ferrooxidans WGS ID MPJE01 1/1-511

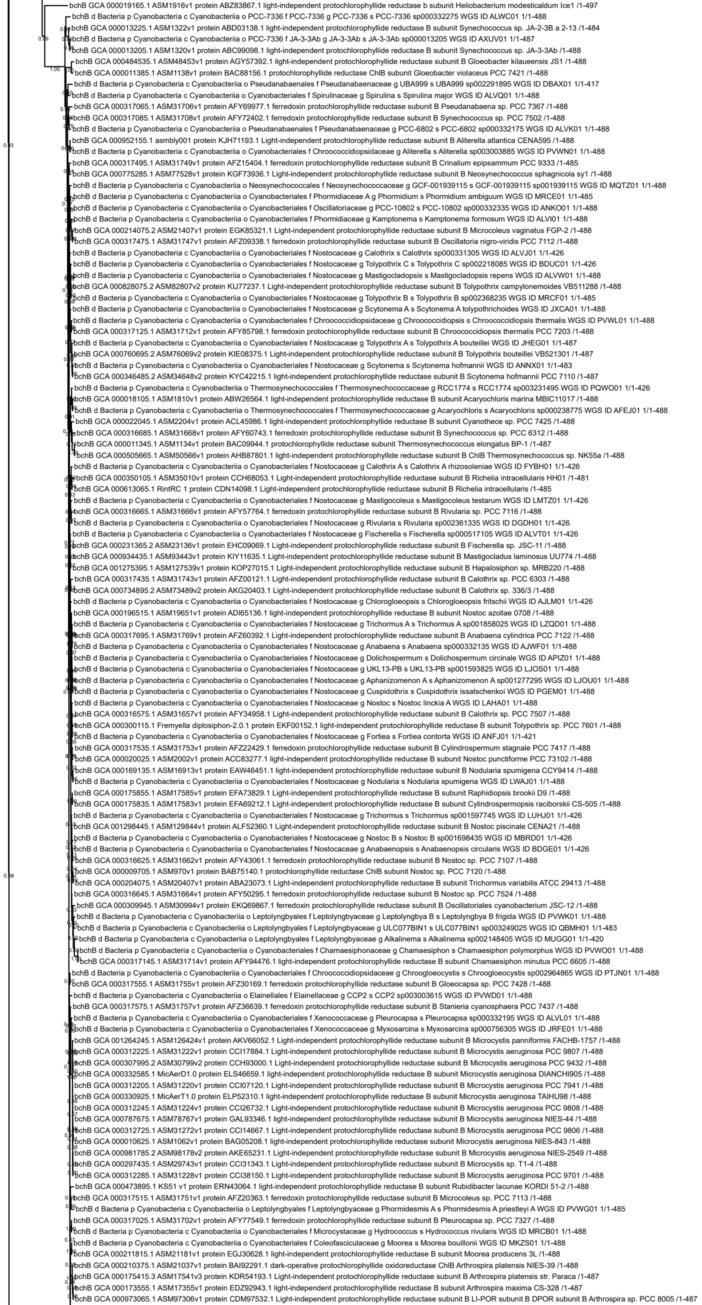
bchB GCA 000019165.1 ASM1916v1 protein ABZ83867.1 light-independent protochlorophyllide reductase b subunit Heliobacterium modesticaldum Ice1 /1-497
bchB d Bacteria p Cyanobacteria c Cyanobacteriia o PCC-7336 f PCC-7336 g PCC-7336 s PCC-7336 sp000332275 WGS ID ALWC01 1/1-488
bchB GCA 000013225.1 ASM1322v1 protein ABD03138.1 light-independent protochlorophyllide reductase B subunit Synechococcus sp. JA-2-3B a 2-13 /1-484
bchB d Bacteria p Cyanobacteria c Cyanobacteriia o PCC-7336 f JA-3-3Ab g JA-3-3Ab s JA-3-3Ab sp000013205 WGS ID AXUV01 1/1-487
bchB GCA 000013205.1 ASM1320v1 protein ABC99098.1 light-independent protochlorophyllide reductase B subunit Synechococcus sp. JA-3-3Ab /1-488
bchB GCA 000484535.1 ASM48453v1 protein AGY57392.1 light-independent protochlorophyllide reductase subunit B Gloeobacter kilaueensis JS1 /1-488
bchB GCA 000011385.1 ASM1138v1 protein BAC88156.1 protochlorophyllide reductase ChlB subunit Gloeobacter violaceus PCC 7421 /1-488
bchB d Bacteria p Cyanobacteria c Cyanobacteriia o Pseudanabaenales f Pseudanabaenaceae g UBA999 s UBA999 sp002291895 WGS ID DBAX01 1/1-417
bchB d Bacteria p Cyanobacteria c Cyanobacteriia o Cyanobacteriales f Spirulinaceae g Spirulina s Spirulina major WGS ID ALVQ01 1/1-488
bchB GCA 000317065.1 ASM31706v1 protein AFY69977.1 ferredoxin protochlorophyllide reductase subunit B Pseudanabaena sp. PCC 7367 /1-488
bchB GCA 000317085.1 ASM31708v1 protein AFY72402.1 ferredoxin protochlorophyllide reductase subunit B Synechococcus sp. PCC 7502 /1-488
bchB d Bacteria p Cyanobacteria c Cyanobacteriia o Pseudanabaenales f Pseudanabaenaceae g PCC-6802 s PCC-6802 sp000332175 WGS ID ALVK01 1/1-488
bchB GCA 000952155.1 asmbly001 protein KJH71193.1 Light-independent protochlorophyllide reductase subunit B Aliterella atlantica CENA595 /1-488
bchB d Bacteria p Cyanobacteria c Cyanobacteriia o Cyanobacteriales f Chroococcidiopsidaceae g Aliterella s Aliterella sp003003885 WGS ID PVWN01 1/1-488
bchB GCA 000317495.1 ASM31749v1 protein AFZ15404.1 ferredoxin protochlorophyllide reductase subunit B Crinalium epipsammum PCC 9333 /1-485
bchB GCA 000775285.1 ASM77528v1 protein KGF73936.1 Light-independent protochlorophyllide reductase subunit B Neosynechococcus sphagnicola sy1 /1-488
bchB d Bacteria p Cyanobacteria c Cyanobacteriia o Neosynechococcales f Neosynechococcaceae g GCF-001939115 s GCF-001939115 sp001939115 WGS ID MQTZ01 1/1-488
bchB d Bacteria p Cyanobacteria c Cyanobacteriia o Cyanobacteriales f Phormidiaceae A g Phormidium s Phormidium ambiguum WGS ID MRCE01 1/1-485
bchB d Bacteria p Cyanobacteria c Cyanobacteriia o Cyanobacteriales f Oscillatoriaceae g PCC-10802 s PCC-10802 sp000332335 WGS ID ANKO01 1/1-488
bchB d Bacteria p Cyanobacteria c Cyanobacteriia o Cyanobacteriales f Phormidiaceae g Kamptonema s Kamptonema formosum WGS ID ALVI01 1/1-488
bchB GCA 000214075.2 ASM21407v1 protein EGK85321.1 Light-independent protochlorophyllide reductase subunit B Microcoleus vaginatus FGP-2 /1-488
bchB GCA 000317475.1 ASM31747v1 protein AFZ09338.1 ferredoxin protochlorophyllide reductase subunit B Oscillatoria nigro-viridis PCC 7112 /1-488
bchB d Bacteria p Cyanobacteria c Cyanobacteriia o Cyanobacteriales f Nostocaceae g Calothrix s Calothrix sp000331305 WGS ID ALVJ01 1/1-426
bchB d Bacteria p Cyanobacteria c Cyanobacteriia o Cyanobacteriales f Nostocaceae g Tolypothrix C s Tolypothrix C sp002218085 WGS ID BDUC01 1/1-426
bchB d Bacteria p Cyanobacteria c Cyanobacteriia o Cyanobacteriales f Nostocaceae g Mastigocladopsis s Mastigocladopsis repens WGS ID ALVW01 1/1-488
bchB GCA 000828075.2 ASM82807v2 protein KIJ77237.1 Light-independent protochlorophyllide reductase subunit B Tolypothrix campylonemoides VB511288 /1-488
bchB d Bacteria p Cyanobacteria c Cyanobacteriia o Cyanobacteriales f Nostocaceae g Tolypothrix B s Tolypothrix B sp002368235 WGS ID MRCF01 1/1-485
bchB d Bacteria p Cyanobacteria c Cyanobacteriia o Cyanobacteriales f Nostocaceae g Scytonema A s Scytonema A tolypothrichoides WGS ID JXCA01 1/1-488
bchB d Bacteria p Cyanobacteria c Cyanobacteriia o Cyanobacteriales f Chroococcidiopsidaceae g Chroococcidiopsis s Chroococcidiopsis thermalis WGS ID PVWL01 1/1-488
bchB GCA 000317125.1 ASM31712v1 protein AFY85798.1 ferredoxin protochlorophyllide reductase subunit B Chroococcidiopsis thermalis PCC 7203 /1-488
bchB d Bacteria p Cyanobacteria c Cyanobacteriia o Cyanobacteriales f Nostocaceae g Tolypothrix A s Tolypothrix A bouteillei WGS ID JHEG01 1/1-487
bchB GCA 000760695.2 ASM76069v2 protein KIE08375.1 Light-independent protochlorophyllide reductase subunit B Tolypothrix bouteillei VB521301 /1-487
bchB d Bacteria p Cyanobacteria c Cyanobacteriia o Cyanobacteriales f Nostocaceae g Scytonema s Scytonema hofmannii WGS ID ANNX01 1/1-483
bchB GCA 000346485.2 ASM34648v2 protein KYC42215.1 light-independent protochlorophyllide reductase subunit B Scytonema hofmannii PCC 7110 /1-487
bchB d Bacteria p Cyanobacteria c Cyanobacteriia o Thermosynechococcales f Thermosynechococcaceae g RCC1774 s RCC1774 sp003231495 WGS ID PQWO01 1/1-426
bchB GCA 000018105.1 ASM1810v1 protein ABW26564.1 light-independent protochlorophyllide reductase B subunit Acaryochloris marina MBIC11017 /1-488
bchB d Bacteria p Cyanobacteria c Cyanobacteriia o Thermosynechococcales f Thermosynechococcaceae g Acaryochloris s Acaryochloris sp000238775 WGS ID AFEJ01 1/1-488
bchB GCA 000022045.1 ASM2204v1 protein ACL45986.1 light-independent protochlorophyllide reductase B subunit Cyanothece sp. PCC 7425 /1-488
bchB GCA 000316685.1 ASM31668v1 protein AFY60743.1 ferredoxin protochlorophyllide reductase subunit B Synechococcus sp. PCC 6312 /1-488
bchB GCA 000011345.1 ASM1134v1 protein BAC09944.1 protochlorophyllide reductase subunit Thermosynechococcus elongatus BP-1 /1-487
bchB GCA 000505665.1 ASM50566v1 protein AHB87801.1 light-independent protochlorophyllide reductase subunit B ChlB Thermosynechococcus sp. NK55a /1-488
bchB d Bacteria p Cyanobacteria c Cyanobacteriia o Cyanobacteriales f Nostocaceae g Calothrix A s Calothrix A rhizosoleniae WGS ID FYBH01 1/1-426
bchB GCA 000350105.1 ASM35010v1 protein CCH68053.1 Light-independent protochlorophyllide reductase subunit B Richelia intracellularis HH01 /1-481
bchB GCA 000613065.1 RintRC 1 protein CDN14098.1 Light-independent protochlorophyllide reductase subunit B Richelia intracellularis /1-485
bchB d Bacteria p Cyanobacteria c Cyanobacteriia o Cyanobacteriales f Nostocaceae g Mastigocoleus s Mastigocoleus testarum WGS ID LMTZ01 1/1-426
bchB GCA 000316665.1 ASM31666v1 protein AFY57764.1 ferredoxin protochlorophyllide reductase subunit B Rivularia sp. PCC 7116 /1-488
bchB d Bacteria p Cyanobacteria c Cyanobacteriia o Cyanobacteriales f Nostocaceae g Rivularia s Rivularia sp002361335 WGS ID DGDH01 1/1-426
bchB d Bacteria p Cyanobacteria c Cyanobacteriia o Cyanobacteriales f Nostocaceae g Fischerella s Fischerella sp000517105 WGS ID ALVT01 1/1-426
bchB GCA 000231365.2 ASM23136v1 protein EHC09069.1 Light-independent protochlorophyllide reductase subunit B Fischerella sp. JSC-11 /1-488
bchB GCA 000934435.1 ASM93443v1 protein KIY11635.1 Light-independent protochlorophyllide reductase subunit B Mastigocladus laminosus UU774 /1-488
bchB GCA 001275395.1 ASM127539v1 protein KOP27015.1 Light-independent protochlorophyllide reductase subunit B Hapalosiphon sp. MRB220 /1-488
bchB GCA 000317435.1 ASM31743v1 protein AFZ00121.1 Light-independent protochlorophyllide reductase subunit B Calothrix sp. PCC 6303 /1-488
bchB GCA 000734895.2 ASM73489v2 protein AKG20403.1 Light-independent protochlorophyllide reductase subunit B Calothrix sp. 336/3 /1-488
bchB d Bacteria p Cyanobacteria c Cyanobacteriia o Cyanobacteriales f Nostocaceae g Chlorogloeopsis s Chlorogloeopsis fritschii WGS ID AJLM01 1/1-426
bchB GCA 000196515.1 ASM19651v1 protein ADI65136.1 light-independent protochlorophyllide reductase B subunit Nostoc azollae 0708 /1-488
bchB d Bacteria p Cyanobacteria c Cyanobacteriia o Cyanobacteriales f Nostocaceae g Trichormus A s Trichormus A sp001858025 WGS ID LZQD01 1/1-488
bchB GCA 000317695.1 ASM31769v1 protein AFZ60392.1 Light-independent protochlorophyllide reductase subunit B Anabaena cylindrica PCC 7122 /1-488
bchB d Bacteria p Cyanobacteria c Cyanobacteriia o Cyanobacteriales f Nostocaceae g Anabaena s Anabaena sp000332135 WGS ID AJWF01 1/1-488
bchB d Bacteria p Cyanobacteria c Cyanobacteriia o Cyanobacteriales f Nostocaceae g Dolichospermum s Dolichospermum circinale WGS ID APIZ01 1/1-488
bchB d Bacteria p Cyanobacteria c Cyanobacteriia o Cyanobacteriales f Nostocaceae g UKL13-PB s UKL13-PB sp001593825 WGS ID LJOS01 1/1-488
bchB d Bacteria p Cyanobacteria c Cyanobacteriia o Cyanobacteriales f Nostocaceae g Aphanizomenon A s Aphanizomenon A sp001277295 WGS ID LJOU01 1/1-488
bchB d Bacteria p Cyanobacteria c Cyanobacteriia o Cyanobacteriales f Nostocaceae g Cuspidothrix s Cuspidothrix issatschenkoi WGS ID PGEM01 1/1-488
bchB d Bacteria p Cyanobacteria c Cyanobacteriia o Cyanobacteriales f Nostocaceae g Nostoc s Nostoc linckia A WGS ID LAHA01 1/1-488
bchB GCA 000316575.1 ASM31657v1 protein AFY34958.1 Light-independent protochlorophyllide reductase subunit B Calothrix sp. PCC 7507 /1-488
bchB GCA 000300115.1 Fremyella diplosiphon-2.0.1 protein EKF00152.1 light-independent protochlorophyllide reductase B subunit Tolypothrix sp. PCC 7601 /1-488
bchB d Bacteria p Cyanobacteria c Cyanobacteriia o Cyanobacteriales f Nostocaceae g Fortiea s Fortiea contorta WGS ID ANFJ01 1/1-421
bchB GCA 000317535.1 ASM31753v1 protein AFZ22429.1 ferredoxin protochlorophyllide reductase subunit B Cylindrospermum stagnale PCC 7417 /1-488
bchB GCA 000020025.1 ASM2002v1 protein ACC83277.1 light-independent protochlorophyllide reductase B subunit Nostoc punctiforme PCC 73102 /1-488
bchB GCA 000169135.1 ASM16913v1 protein EAW46451.1 light-independent protochlorophyllide reductase subunit B Nodularia spumigena CCY9414 /1-488
bchB d Bacteria p Cyanobacteria c Cyanobacteriia o Cyanobacteriales f Nostocaceae g Nodularia s Nodularia spumigena WGS ID LWAJ01 1/1-488
bchB GCA 000175855.1 ASM17585v1 protein EFA73829.1 Light-independent protochlorophyllide reductase B subunit Raphidiopsis brookii D9 /1-488
bchB GCA 000175835.1 ASM17583v1 protein EFA69212.1 Light-independent protochlorophyllide reductase B subunit Cylindrospermopsis raciborskii CS-505 /1-488
bchB d Bacteria p Cyanobacteria c Cyanobacteriia o Cyanobacteriales f Nostocaceae g Trichormus s Trichormus sp001597745 WGS ID LUHJ01 1/1-426
bchB GCA 001298445.1 ASM129844v1 protein ALF52360.1 Light-independent protochlorophyllide reductase subunit B Nostoc piscinale CENA21 /1-488
bchB d Bacteria p Cyanobacteria c Cyanobacteriia o Cyanobacteriales f Nostocaceae g Nostoc B s Nostoc B sp001698435 WGS ID MBRD01 1/1-426
bchB d Bacteria p Cyanobacteria c Cyanobacteriia o Cyanobacteriales f Nostocaceae g Anabaenopsis s Anabaenopsis circularis WGS ID BDGE01 1/1-426
bchB GCA 000316625.1 ASM31662v1 protein AFY43061.1 ferredoxin protochlorophyllide reductase subunit B Nostoc sp. PCC 7107 /1-488
bchB GCA 000009705.1 ASM970v1 protein BAB75140.1 protochlorophyllide reductase ChlB subunit Nostoc sp. PCC 7120 /1-488
bchB GCA 000204075.1 ASM20407v1 protein ABA23073.1 light-independent protochlorophyllide reductase B subunit Trichormus variabilis ATCC 29413 /1-488
bchB GCA 000316645.1 ASM31664v1 protein AFY50295.1 ferredoxin protochlorophyllide reductase subunit B Nostoc sp. PCC 7524 /1-488
bchB GCA 000309945.1 ASM30994v1 protein EKQ69867.1 ferredoxin protochlorophyllide reductase subunit B Oscillatoriales cyanobacterium JSC-12 /1-488
bchB d Bacteria p Cyanobacteria c Cyanobacteriia o Leptolyngbyales f Leptolyngbyaceae g Leptolyngbya B s Leptolyngbya B frigida WGS ID PVWK01 1/1-488
bchB d Bacteria p Cyanobacteria c Cyanobacteriia o Leptolyngbyales f Leptolyngbyaceae g ULC077BIN1 s ULC077BIN1 sp003249025 WGS ID QBMH01 1/1-483
bchB d Bacteria p Cyanobacteria c Cyanobacteriia o Leptolyngbyales f Leptolyngbyaceae g Alkalinema s Alkalinema sp002148405 WGS ID MUGG01 1/1-420
bchB d Bacteria p Cyanobacteria c Cyanobacteriia o Cyanobacteriales f Chamaesiphonaceae g Chamaesiphon s Chamaesiphon polymorphus WGS ID PVWO01 1/1-488
bchB GCA 000317145.1 ASM31714v1 protein AFY94476.1 light-independent protochlorophyllide reductase subunit B Chamaesiphon minutus PCC 6605 /1-488
bchB d Bacteria p Cyanobacteria c Cyanobacteriia o Cyanobacteriales f Chroococcidiopsidaceae g Chroogloeocystis s Chroogloeocystis sp002964865 WGS ID PTJN01 1/1-488
bchB GCA 000317555.1 ASM31755v1 protein AFZ30169.1 ferredoxin protochlorophyllide reductase subunit B Gloeocapsa sp. PCC 7428 /1-488
bchB d Bacteria p Cyanobacteria c Cyanobacteriia o Elainellales f Elainellaceae g CCP2 s CCP2 sp003003615 WGS ID PVWD01 1/1-488
bchB GCA 000317575.1 ASM31757v1 protein AFZ36639.1 ferredoxin protochlorophyllide reductase subunit B Stanieria cyanosphaera PCC 7437 /1-488
bchB d Bacteria p Cyanobacteria c Cyanobacteriia o Cyanobacteriales f Xenococcaceae g Pleurocapsa s Pleurocapsa sp000332195 WGS ID ALVL01 1/1-488
bchB d Bacteria p Cyanobacteria c Cyanobacteriia o Cyanobacteriales f Xenococcaceae g Myxosarcina s Myxosarcina sp000756305 WGS ID JRFE01 1/1-488
bchB GCA 001264245.1 ASM126424v1 protein AKV66052.1 Light-independent protochlorophyllide reductase subunit B Microcystis panniformis FACHB-1757 /1-488
bchB GCA 000312225.1 ASM31222v1 protein CCI17884.1 Light-independent protochlorophyllide reductase subunit B Microcystis aeruginosa PCC 9807 /1-488
bchB GCA 000307995.2 ASM30799v2 protein CCH93000.1 Light-independent protochlorophyllide reductase subunit B Microcystis aeruginosa PCC 9432 /1-488
bchB GCA 000332585.1 MicAerD1.0 protein ELS46659.1 light-independent protochlorophyllide reductase B subunit Microcystis aeruginosa DIANCHI905 /1-488
bchB GCA 000312205.1 ASM31220v1 protein CCI07120.1 Light-independent protochlorophyllide reductase subunit B Microcystis aeruginosa PCC 7941 /1-488
bchB GCA 000330925.1 MicAerT1.0 protein ELP52310.1 light-independent protochlorophyllide reductase subunit Microcystis aeruginosa TAIHU98 /1-488
bchB GCA 000312245.1 ASM31224v1 protein CCI26732.1 Light-independent protochlorophyllide reductase subunit B Microcystis aeruginosa PCC 9808 /1-488
bchB GCA 000787675.1 ASM78767v1 protein GAL93346.1 light-independent protochlorophyllide reductase subunit B Microcystis aeruginosa NIES-44 /1-488
bchB GCA 000312725.1 ASM31272v1 protein CCI14667.1 Light-independent protochlorophyllide reductase subunit B Microcystis aeruginosa PCC 9806 /1-488
bchB GCA 000010625.1 ASM1062v1 protein BAG05208.1 light-independent protochlorophyllide reductase subunit Microcystis aeruginosa NIES-843 /1-488
bchB GCA 000981785.2 ASM98178v2 protein AKE65231.1 Light-independent protochlorophyllide reductase subunit B Microcystis aeruginosa NIES-2549 /1-488
bchB GCA 000297435.1 ASM29743v1 protein CCI31343.1 Light-independent protochlorophyllide reductase subunit B Microcystis sp. T1-4 /1-488
bchB GCA 000312285.1 ASM31228v1 protein CCI38150.1 Light-independent protochlorophyllide reductase subunit B Microcystis aeruginosa PCC 9701 /1-488
bchB GCA 000473895.1 KS51 v1 protein ERN43064.1 light-independent protochlorophyllide reductase B subunit Rubidibacter lacunae KORDI 51-2 /1-488
bchB GCA 000317515.1 ASM31751v1 protein AFZ20363.1 ferredoxin protochlorophyllide reductase subunit B Microcoleus sp. PCC 7113 /1-488
bchB d Bacteria p Cyanobacteria c Cyanobacteriia o Leptolyngbyales f Leptolyngbyaceae g Phormidesmis A s Phormidesmis A priestleyi A WGS ID PVWG01 1/1-485
bchB GCA 000317025.1 ASM31702v1 protein AFY77549.1 ferredoxin protochlorophyllide reductase subunit B Pleurocapsa sp. PCC 7327 /1-488
bchB d Bacteria p Cyanobacteria c Cyanobacteriia o Cyanobacteriales f Microcystaceae g Hydrococcus s Hydrococcus rivularis WGS ID MRCB01 1/1-488
bchB d Bacteria p Cyanobacteria c Cyanobacteriia o Cyanobacteriales f Coleofasciculaceae g Moorea s Moorea bouillonii WGS ID MKZS01 1/1-488
bchB GCA 000211815.1 ASM21181v1 protein EGJ30628.1 light-independent protochlorophyllide reductase B subunit Moorea producens 3L /1-488
bchB GCA 000210375.1 ASM21037v1 protein BAI92291.1 dark-operative protochlorophyllide oxidoreductase ChlB Arthrospira platensis NIES-39 /1-488
bchB GCA 000175415.3 ASM17541v3 protein KDR54193.1 Light-independent protochlorophyllide reductase subunit B Arthrospira platensis str. Paraca /1-487
bchB GCA 000173555.1 ASM17355v1 protein EDZ92943.1 light-independent protochlorophyllide reductase subunit B Arthrospira maxima CS-328 /1-487
bchB GCA 000973065.1 ASM97306v1 protein CDM97532.1 Light-independent protochlorophyllide reductase subunit B LI-POR subunit B DPOR subunit B Arthrospira sp. PCC 8005 /1-487

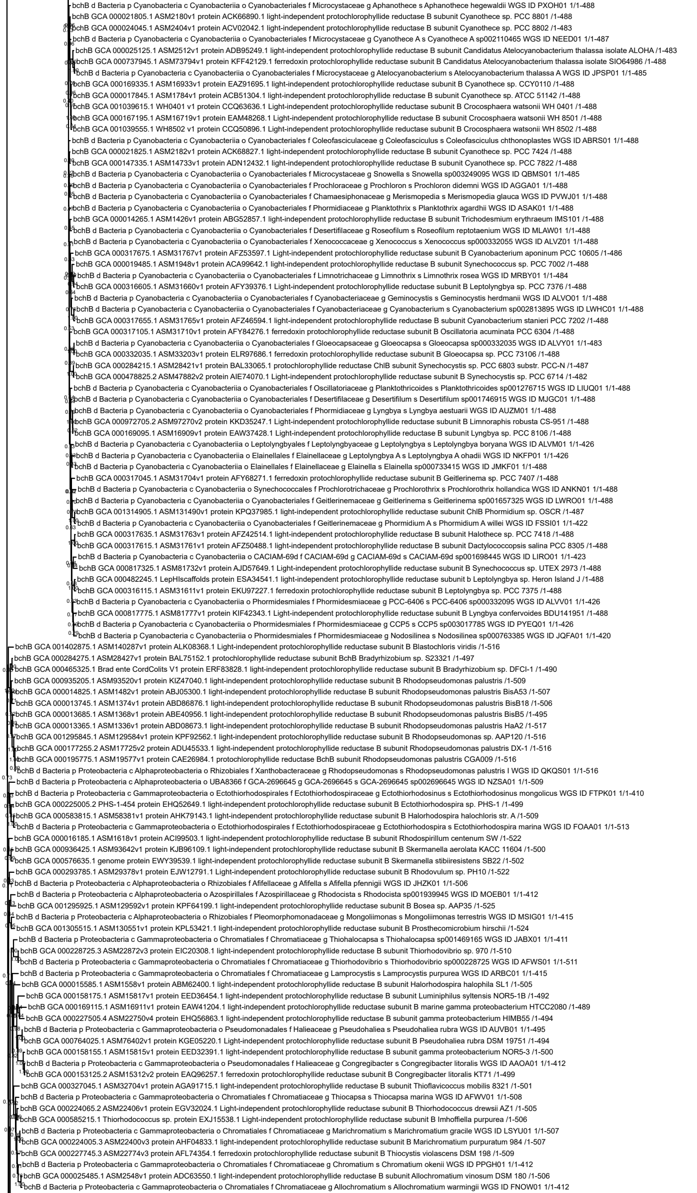
bchB d Bacteria p Cyanobacteria c Cyanobacteriia o Cyanobacteriales f Microcystaceae g Aphanothece s Aphanothece hegewaldii WGS ID PXOH01 1/1-488
bchB GCA 000021805.1 ASM2180v1 protein ACK66890.1 light-independent protochlorophyllide reductase B subunit Cyanothece sp. PCC 8801 /1-488
bchB GCA 000024045.1 ASM2404v1 protein ACV02042.1 light-independent protochlorophyllide reductase B subunit Cyanothece sp. PCC 8802 /1-483
bchB d Bacteria p Cyanobacteria c Cyanobacteriia o Cyanobacteriales f Microcystaceae g Cyanothece A s Cyanothece A sp002110465 WGS ID NEED01 1/1-487
bchB GCA 000025125.1 ASM2512v1 protein ADB95249.1 light-independent protochlorophyllide reductase B subunit Candidatus Atelocyanobacterium thalassa isolate ALOHA /1-483
bchB GCA 000737945.1 ASM73794v1 protein KFF42129.1 ferredoxin protochlorophyllide reductase subunit B Candidatus Atelocyanobacterium thalassa isolate SIO64986 /1-488
bchB d Bacteria p Cyanobacteria c Cyanobacteriia o Cyanobacteriales f Microcystaceae g Atelocyanobacterium s Atelocyanobacterium thalassa A WGS ID JPSP01 1/1-485
bchB GCA 000169335.1 ASM16933v1 protein EAZ91695.1 light-independent protochlorophyllide reductase subunit B Cyanothece sp. CCY0110 /1-488
bchB GCA 000017845.1 ASM1784v1 protein ACB51304.1 light-independent protochlorophyllide reductase B subunit Cyanothece sp. ATCC 51142 /1-488
bchB GCA 001039615.1 WH0401 v1 protein CCQ63636.1 Light-independent protochlorophyllide reductase subunit B Crocosphaera watsonii WH 0401 /1-488
bchB GCA 000167195.1 ASM16719v1 protein EAM48268.1 Light-independent protochlorophyllide reductase B subunit Crocosphaera watsonii WH 8501 /1-488
bchB GCA 001039555.1 WH8502 v1 protein CCQ50896.1 Light-independent protochlorophyllide reductase subunit B Crocosphaera watsonii WH 8502 /1-488
bchB d Bacteria p Cyanobacteria c Cyanobacteriia o Cyanobacteriales f Coleofasciculaceae g Coleofasciculus s Coleofasciculus chthonoplastes WGS ID ABRS01 1/1-488
bchB GCA 000021825.1 ASM2182v1 protein ACK68827.1 light-independent protochlorophyllide reductase B subunit Cyanothece sp. PCC 7424 /1-488
bchB GCA 000147335.1 ASM14733v1 protein ADN12432.1 light-independent protochlorophyllide reductase B subunit Cyanothece sp. PCC 7822 /1-488
bchB d Bacteria p Cyanobacteria c Cyanobacteriia o Cyanobacteriales f Microcystaceae g Snowella s Snowella sp003249095 WGS ID QBMS01 1/1-485
bchB d Bacteria p Cyanobacteria c Cyanobacteriia o Cyanobacteriales f Prochloraceae g Prochloron s Prochloron didemni WGS ID AGGA01 1/1-488
bchB d Bacteria p Cyanobacteria c Cyanobacteriia o Cyanobacteriales f Chamaesiphonaceae g Merismopedia s Merismopedia glauca WGS ID PVWJ01 1/1-488
bchB d Bacteria p Cyanobacteria c Cyanobacteriia o Cyanobacteriales f Phormidiaceae g Planktothrix s Planktothrix agardhii WGS ID ASAK01 1/1-488
bchB GCA 000014265.1 ASM1426v1 protein ABG52857.1 light-independent protochlorophyllide reductase B subunit Trichodesmium erythraeum IMS101 /1-488
bchB d Bacteria p Cyanobacteria c Cyanobacteriia o Cyanobacteriales f Desertifilaceae g Roseofilum s Roseofilum reptotaenium WGS ID MLAW01 1/1-488
bchB d Bacteria p Cyanobacteria c Cyanobacteriia o Cyanobacteriales f Xenococcaceae g Xenococcus s Xenococcus sp000332055 WGS ID ALVZ01 1/1-488
bchB GCA 000317675.1 ASM31767v1 protein AFZ53597.1 Light-independent protochlorophyllide reductase subunit B Cyanobacterium aponinum PCC 10605 /1-486
bchB GCA 000019485.1 ASM1948v1 protein ACA99642.1 light-independent protochlorophyllide reductase B subunit Synechococcus sp. PCC 7002 /1-488
bchB d Bacteria p Cyanobacteria c Cyanobacteriia o Cyanobacteriales f Limnotrichaceae g Limnothrix s Limnothrix rosea WGS ID MRBY01 1/1-484
bchB GCA 000316605.1 ASM31660v1 protein AFY39376.1 Light-independent protochlorophyllide reductase subunit B Leptolyngbya sp. PCC 7376 /1-488
bchB d Bacteria p Cyanobacteria c Cyanobacteriia o Cyanobacteriales f Cyanobacteriaceae g Geminocystis s Geminocystis herdmanii WGS ID ALVO01 1/1-488
bchB d Bacteria p Cyanobacteria c Cyanobacteriia o Cyanobacteriales f Cyanobacteriaceae g Cyanobacterium s Cyanobacterium sp002813895 WGS ID LWHC01 1/1-488
bchB GCA 000317655.1 ASM31765v1 protein AFZ46594.1 Light-independent protochlorophyllide reductase subunit B Cyanobacterium stanieri PCC 7202 /1-488
bchB GCA 000317105.1 ASM31710v1 protein AFY84276.1 ferredoxin protochlorophyllide reductase subunit B Oscillatoria acuminata PCC 6304 /1-488
bchB d Bacteria p Cyanobacteria c Cyanobacteriia o Cyanobacteriales f Gloeocapsaceae g Gloeocapsa s Gloeocapsa sp000332035 WGS ID ALVY01 1/1-483
bchB GCA 000332035.1 ASM33203v1 protein ELR97686.1 ferredoxin protochlorophyllide reductase subunit B Gloeocapsa sp. PCC 73106 /1-488
bchB GCA 000284215.1 ASM28421v1 protein BAL33065.1 protochlorophyllide reductase ChlB subunit Synechocystis sp. PCC 6803 substr. PCC-N /1-487
bchB GCA 000478825.2 ASM47882v2 protein AIE74070.1 Light-independent protochlorophyllide reductase subunit B Synechocystis sp. PCC 6714 /1-482
bchB d Bacteria p Cyanobacteria c Cyanobacteriia o Cyanobacteriales f Oscillatoriaceae g Planktothricoides s Planktothricoides sp001276715 WGS ID LIUQ01 1/1-488
bchB d Bacteria p Cyanobacteria c Cyanobacteriia o Cyanobacteriales f Desertifilaceae g Desertifilum s Desertifilum sp001746915 WGS ID MJGC01 1/1-488
bchB d Bacteria p Cyanobacteria c Cyanobacteriia o Cyanobacteriales f Phormidiaceae g Lyngbya s Lyngbya aestuarii WGS ID AUZM01 1/1-488
bchB GCA 000972705.2 ASM97270v2 protein KKD35247.1 Light-independent protochlorophyllide reductase subunit B Limnoraphis robusta CS-951 /1-488
bchB GCA 000169095.1 ASM16909v1 protein EAW37428.1 Light-independent protochlorophyllide reductase B subunit Lyngbya sp. PCC 8106 /1-488
bchB d Bacteria p Cyanobacteria c Cyanobacteriia o Leptolyngbyales f Leptolyngbyaceae g Leptolyngbya s Leptolyngbya boryana WGS ID ALVM01 1/1-426
bchB d Bacteria p Cyanobacteria c Cyanobacteriia o Elainellales f Elainellaceae g Leptolyngbya A s Leptolyngbya A ohadii WGS ID NKFP01 1/1-426
bchB d Bacteria p Cyanobacteria c Cyanobacteriia o Elainellales f Elainellaceae g Elainella s Elainella sp000733415 WGS ID JMKF01 1/1-488
bchB GCA 000317045.1 ASM31704v1 protein AFY68271.1 ferredoxin protochlorophyllide reductase subunit B Geitlerinema sp. PCC 7407 /1-488
bchB d Bacteria p Cyanobacteria c Cyanobacteriia o Synechococcales f Prochlorotrichaceae g Prochlorothrix s Prochlorothrix hollandica WGS ID ANKN01 1/1-488
bchB d Bacteria p Cyanobacteria c Cyanobacteriia o Cyanobacteriales f Geitlerinemaceae g Geitlerinema s Geitlerinema sp001657325 WGS ID LWRO01 1/1-488
bchB GCA 001314905.1 ASM131490v1 protein KPQ37985.1 light-independent protochlorophyllide reductase subunit ChlB Phormidium sp. OSCR /1-487
bchB d Bacteria p Cyanobacteria c Cyanobacteriia o Cyanobacteriales f Geitlerinemaceae g Phormidium A s Phormidium A willei WGS ID FSSI01 1/1-422
bchB GCA 000317635.1 ASM31763v1 protein AFZ42514.1 light-independent protochlorophyllide reductase B subunit Halothece sp. PCC 7418 /1-488
bchB GCA 000317615.1 ASM31761v1 protein AFZ50488.1 light-independent protochlorophyllide reductase B subunit Dactylococcopsis salina PCC 8305 /1-488
bchB d Bacteria p Cyanobacteria c Cyanobacteriia o CACIAM-69d f CACIAM-69d g CACIAM-69d s CACIAM-69d sp001698445 WGS ID LIRO01 1/1-423
bchB GCA 000817325.1 ASM81732v1 protein AJD57649.1 Light-independent protochlorophyllide reductase subunit B Synechococcus sp. UTEX 2973 /1-488
bchB GCA 000482245.1 LepHIscaffolds protein ESA34541.1 light-independent protochlorophyllide reductase subunit b Leptolyngbya sp. Heron Island J /1-488
bchB GCA 000316115.1 ASM31611v1 protein EKU97227.1 ferredoxin protochlorophyllide reductase subunit B Leptolyngbya sp. PCC 7375 /1-488
bchB d Bacteria p Cyanobacteria c Cyanobacteriia o Phormidesmiales f Phormidesmiaceae g PCC-6406 s PCC-6406 sp000332095 WGS ID ALVV01 1/1-426
bchB GCA 000817775.1 ASM81777v1 protein KIF42343.1 Light-independent protochlorophyllide reductase subunit B Lyngbya confervoides BDU141951 /1-488
bchB d Bacteria p Cyanobacteria c Cyanobacteriia o Phormidesmiales f Phormidesmiaceae g CCP5 s CCP5 sp003017785 WGS ID PYEQ01 1/1-426
bchB d Bacteria p Cyanobacteria c Cyanobacteriia o Phormidesmiales f Phormidesmiaceae g Nodosilinea s Nodosilinea sp000763385 WGS ID JQFA01 1/1-420
bchB GCA 001402875.1 ASM140287v1 protein ALK08368.1 Light-independent protochlorophyllide reductase subunit B Blastochloris viridis /1-516
bchB GCA 000284275.1 ASM28427v1 protein BAL75152.1 protochlorophyllide reductase subunit BchB Bradyrhizobium sp. S23321 /1-497
bchB GCA 000465325.1 Brad ente CordColits V1 protein ERF83828.1 light-independent protochlorophyllide reductase subunit B Bradyrhizobium sp. DFCI-1 /1-490
bchB GCA 000935205.1 ASM93520v1 protein KIZ47040.1 light-independent protochlorophyllide reductase subunit B Rhodopseudomonas palustris /1-509
bchB GCA 000014825.1 ASM1482v1 protein ABJ05300.1 light-independent protochlorophyllide reductase B subunit Rhodopseudomonas palustris BisA53 /1-507
bchB GCA 000013745.1 ASM1374v1 protein ABD86876.1 light-independent protochlorophyllide reductase B subunit Rhodopseudomonas palustris BisB18 /1-506
bchB GCA 000013685.1 ASM1368v1 protein ABE40956.1 light-independent protochlorophyllide reductase B subunit Rhodopseudomonas palustris BisB5 /1-495
bchB GCA 000013365.1 ASM1336v1 protein ABD08673.1 light-independent protochlorophyllide reductase B subunit Rhodopseudomonas palustris HaA2 /1-517
bchB GCA 001295845.1 ASM129584v1 protein KPF92562.1 light-independent protochlorophyllide reductase subunit B Rhodopseudomonas sp. AAP120 /1-516
bchB GCA 000177255.2 ASM17725v2 protein ADU45533.1 light-independent protochlorophyllide reductase B subunit Rhodopseudomonas palustris DX-1 /1-516
bchB GCA 000195775.1 ASM19577v1 protein CAE26984.1 protochlorophyllide reductase BchB subunit Rhodopseudomonas palustris CGA009 /1-516
bchB d Bacteria p Proteobacteria c Alphaproteobacteria o Rhizobiales f Xanthobacteraceae g Rhodopseudomonas s Rhodopseudomonas palustris I WGS ID QKQS01 1/1-516
bchB d Bacteria p Proteobacteria c Alphaproteobacteria o UBA8366 f GCA-2696645 g GCA-2696645 s GCA-2696645 sp002696645 WGS ID NZSA01 1/1-509
bchB d Bacteria p Proteobacteria c Gammaproteobacteria o Ectothiorhodospirales f Ectothiorhodospiraceae g Ectothiorhodosinus s Ectothiorhodosinus mongolicus WGS ID FTPK01 1/1-410
bchB GCA 000225005.2 PHS-1-454 protein EHQ52649.1 light-independent protochlorophyllide reductase subunit B Ectothiorhodospira sp. PHS-1 /1-499
bchB GCA 000583815.1 ASM58381v1 protein AHK79143.1 light-independent protochlorophyllide reductase subunit B Halorhodospira halochloris str. A /1-509
bchB d Bacteria p Proteobacteria c Gammaproteobacteria o Ectothiorhodospirales f Ectothiorhodospiraceae g Ectothiorhodospira s Ectothiorhodospira marina WGS ID FOAA01 1/1-513
bchB GCA 000016185.1 ASM1618v1 protein ACI99503.1 light-independent protochlorophyllide reductase B subunit Rhodospirillum centenum SW /1-522
bchB GCA 000936425.1 ASM93642v1 protein KJB96109.1 light-independent protochlorophyllide reductase subunit B Skermanella aerolata KACC 11604 /1-500
bchB GCA 000576635.1 genome protein EWY39539.1 light-independent protochlorophyllide reductase subunit B Skermanella stibiiresistens SB22 /1-502
bchB GCA 000293785.1 ASM29378v1 protein EJW12791.1 Light-independent protochlorophyllide reductase subunit B Rhodovulum sp. PH10 /1-522
bchB d Bacteria p Proteobacteria c Alphaproteobacteria o Rhizobiales f Afifellaceae g Afifella s Afifella pfennigii WGS ID JHZK01 1/1-506
bchB d Bacteria p Proteobacteria c Alphaproteobacteria o Azospirillales f Azospirillaceae g Rhodocista s Rhodocista sp001939945 WGS ID MOEB01 1/1-412
bchB GCA 001295925.1 ASM129592v1 protein KPF64199.1 light-independent protochlorophyllide reductase subunit B Bosea sp. AAP35 /1-525
bchB d Bacteria p Proteobacteria c Alphaproteobacteria o Rhizobiales f Pleomorphomonadaceae g Mongoliimonas s Mongoliimonas terrestris WGS ID MSIG01 1/1-415
bchB GCA 001305515.1 ASM130551v1 protein KPL53421.1 light-independent protochlorophyllide reductase subunit B Prosthecomicrobium hirschii /1-524
bchB d Bacteria p Proteobacteria c Gammaproteobacteria o Chromatiales f Chromatiaceae g Thiohalocapsa s Thiohalocapsa sp001469165 WGS ID JABX01 1/1-411
bchB GCA 000228725.3 ASM22872v3 protein EIC20308.1 light-independent protochlorophyllide reductase B subunit Thiorhodovibrio sp. 970 /1-510
bchB d Bacteria p Proteobacteria c Gammaproteobacteria o Chromatiales f Chromatiaceae g Thiorhodovibrio s Thiorhodovibrio sp000228725 WGS ID AFWS01 1/1-511
bchB d Bacteria p Proteobacteria c Gammaproteobacteria o Chromatiales f Chromatiaceae g Lamprocystis s Lamprocystis purpurea WGS ID ARBC01 1/1-415
bchB GCA 000015585.1 ASM1558v1 protein ABM62400.1 light-independent protochlorophyllide reductase B subunit Halorhodospira halophila SL1 /1-505
bchB GCA 000158175.1 ASM15817v1 protein EED36454.1 light-independent protochlorophyllide reductase B subunit Luminiphilus syltensis NOR5-1B /1-492
bchB GCA 000169115.1 ASM16911v1 protein EAW41204.1 light-independent protochlorophyllide reductase subunit B marine gamma proteobacterium HTCC2080 /1-489
bchB GCA 000227505.4 ASM22750v4 protein EHQ56863.1 light-independent protochlorophyllide reductase B subunit gamma proteobacterium HIMB55 /1-494
bchB d Bacteria p Proteobacteria c Gammaproteobacteria o Pseudomonadales f Halieaceae g Pseudohaliea s Pseudohaliea rubra WGS ID AUVB01 1/1-495
bchB GCA 000764025.1 ASM76402v1 protein KGE05220.1 Light-independent protochlorophyllide reductase subunit B Pseudohaliea rubra DSM 19751 /1-494
bchB GCA 000158155.1 ASM15815v1 protein EED32391.1 light-independent protochlorophyllide reductase B subunit gamma proteobacterium NOR5-3 /1-500
bchB d Bacteria p Proteobacteria c Gammaproteobacteria o Pseudomonadales f Halieaceae g Congregibacter s Congregibacter litoralis WGS ID AAOA01 1/1-412
bchB GCA 000153125.2 ASM15312v2 protein EAQ96257.1 ferredoxin protochlorophyllide reductase subunit B Congregibacter litoralis KT71 /1-499
bchB GCA 000327045.1 ASM32704v1 protein AGA91715.1 light-independent protochlorophyllide reductase B subunit Thioflavicoccus mobilis 8321 /1-501
bchB d Bacteria p Proteobacteria c Gammaproteobacteria o Chromatiales f Chromatiaceae g Thiocapsa s Thiocapsa marina WGS ID AFWV01 1/1-508
bchB GCA 000224065.2 ASM22406v1 protein EGV32024.1 Light-independent protochlorophyllide reductase subunit B Thiorhodococcus drewsii AZ1 /1-505
bchB GCA 000585215.1 Thiorhodococcus sp. protein EXJ15538.1 Light-independent protochlorophyllide reductase subunit B Imhoffiella purpurea /1-506
bchB d Bacteria p Proteobacteria c Gammaproteobacteria o Chromatiales f Chromatiaceae g Marichromatium s Marichromatium gracile WGS ID LSYU01 1/1-507
bchB GCA 000224005.3 ASM22400v3 protein AHF04833.1 light-independent protochlorophyllide reductase subunit B Marichromatium purpuratum 984 /1-507
bchB GCA 000227745.3 ASM22774v3 protein AFL74354.1 ferredoxin protochlorophyllide reductase subunit B Thiocystis violascens DSM 198 /1-509
bchB d Bacteria p Proteobacteria c Gammaproteobacteria o Chromatiales f Chromatiaceae g Chromatium s Chromatium okenii WGS ID PPGH01 1/1-412
bchB GCA 000025485.1 ASM2548v1 protein ADC63550.1 light-independent protochlorophyllide reductase B subunit Allochromatium vinosum DSM 180 /1-506
bchB d Bacteria p Proteobacteria c Gammaproteobacteria o Chromatiales f Chromatiaceae g Allochromatium s Allochromatium warmingii WGS ID FNOW01 1/1-412

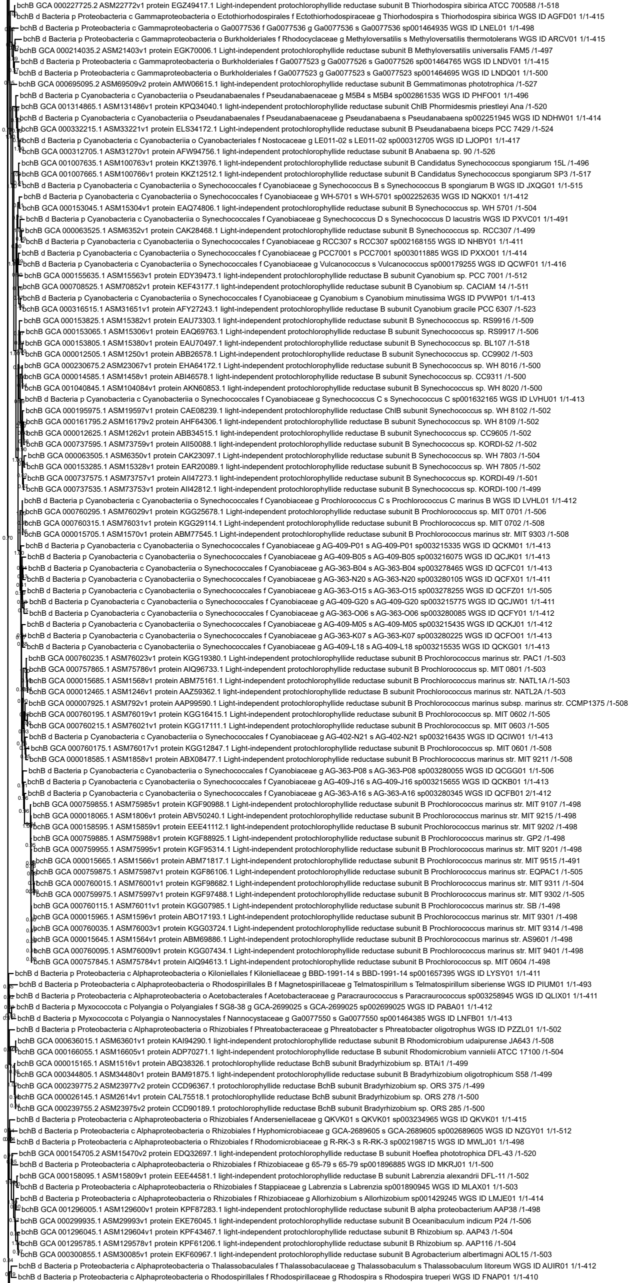
bchB GCA 000227725.2 ASM22772v1 protein EGZ49417.1 Light-independent protochlorophyllide reductase subunit B Thiorhodospira sibirica ATCC 700588 /1-518
bchB d Bacteria p Proteobacteria c Gammaproteobacteria o Ectothiorhodospirales f Ectothiorhodospiraceae g Thiorhodospira s Thiorhodospira sibirica WGS ID AGFD01 1/1-415
bchB d Bacteria p Proteobacteria c Gammaproteobacteria o Ga0077536 f Ga0077536 g Ga0077536 s Ga0077536 sp001464935 WGS ID LNEL01 1/1-498
bchB d Bacteria p Proteobacteria c Gammaproteobacteria o Burkholderiales f Rhodocyclaceae g Methyloversatilis s Methyloversatilis thermotolerans WGS ID ARCV01 1/1-415
bchB GCA 000214035.2 ASM21403v1 protein EGK70006.1 Light-independent protochlorophyllide reductase subunit B Methyloversatilis universalis FAM5 /1-497
bchB d Bacteria p Proteobacteria c Gammaproteobacteria o Burkholderiales f Ga0077523 g Ga0077526 s Ga0077526 sp001464765 WGS ID LNDV01 1/1-415
bchB d Bacteria p Proteobacteria c Gammaproteobacteria o Burkholderiales f Ga0077523 g Ga0077523 s Ga0077523 sp001464695 WGS ID LNDQ01 1/1-500
bchB GCA 000695095.2 ASM69509v2 protein AMW06615.1 light-independent protochlorophyllide reductase subunit B Gemmatimonas phototrophica /1-527
bchB d Bacteria p Cyanobacteria c Cyanobacteriia o Pseudanabaenales f Pseudanabaenaceae g M5B4 s M5B4 sp002861535 WGS ID PHFO01 1/1-496
bchB GCA 001314865.1 ASM131486v1 protein KPQ34040.1 light-independent protochlorophyllide reductase subunit ChlB Phormidesmis priestleyi Ana /1-520
bchB d Bacteria p Cyanobacteria c Cyanobacteriia o Pseudanabaenales f Pseudanabaenaceae g Pseudanabaena s Pseudanabaena sp002251945 WGS ID NDHW01 1/1-414
bchB GCA 000332215.1 ASM33221v1 protein ELS34172.1 Light-independent protochlorophyllide reductase subunit B Pseudanabaena biceps PCC 7429 /1-524
bchB d Bacteria p Cyanobacteria c Cyanobacteriia o Cyanobacteriales f Nostocaceae g LE011-02 s LE011-02 sp000312705 WGS ID LJOP01 1/1-417
bchB GCA 000312705.1 ASM31270v1 protein AFW94756.1 light-independent protochlorophyllide reductase subunit B Anabaena sp. 90 /1-526
bchB GCA 001007635.1 ASM100763v1 protein KKZ13976.1 light-independent protochlorophyllide reductase subunit B Candidatus Synechococcus spongiarum 15L /1-496
bchB GCA 001007665.1 ASM100766v1 protein KKZ12512.1 light-independent protochlorophyllide reductase subunit B Candidatus Synechococcus spongiarum SP3 /1-517
bchB d Bacteria p Cyanobacteria c Cyanobacteriia o Synechococcales f Cyanobiaceae g Synechococcus B s Synechococcus B spongiarum B WGS ID JXQG01 1/1-515
bchB d Bacteria p Cyanobacteria c Cyanobacteriia o Synechococcales f Cyanobiaceae g WH-5701 s WH-5701 sp002252635 WGS ID NQKX01 1/1-412
bchB GCA 000153045.1 ASM15304v1 protein EAQ74806.1 light-independent protochlorophyllide reductase subunit B Synechococcus sp. WH 5701 /1-504
bchB d Bacteria p Cyanobacteria c Cyanobacteriia o Synechococcales f Cyanobiaceae g Synechococcus D s Synechococcus D lacustris WGS ID PXVC01 1/1-491
bchB GCA 000063525.1 ASM6352v1 protein CAK28468.1 Light-independent protochlorophyllide reductase subunit B Synechococcus sp. RCC307 /1-499
bchB d Bacteria p Cyanobacteria c Cyanobacteriia o Synechococcales f Cyanobiaceae g RCC307 s RCC307 sp002168155 WGS ID NHBY01 1/1-411
bchB d Bacteria p Cyanobacteria c Cyanobacteriia o Synechococcales f Cyanobiaceae g PCC7001 s PCC7001 sp003011885 WGS ID PXXO01 1/1-414
bchB d Bacteria p Cyanobacteria c Cyanobacteriia o Synechococcales f Cyanobiaceae g Vulcanococcus s Vulcanococcus sp000179255 WGS ID QCWF01 1/1-416
bchB GCA 000155635.1 ASM15563v1 protein EDY39473.1 light-independent protochlorophyllide reductase B subunit Cyanobium sp. PCC 7001 /1-512
bchB GCA 000708525.1 ASM70852v1 protein KEF43177.1 light-independent protochlorophyllide reductase subunit B Cyanobium sp. CACIAM 14 /1-511
bchB d Bacteria p Cyanobacteria c Cyanobacteriia o Synechococcales f Cyanobiaceae g Cyanobium s Cyanobium minutissima WGS ID PVWP01 1/1-413
bchB GCA 000316515.1 ASM31651v1 protein AFY27243.1 light-independent protochlorophyllide reductase B subunit Cyanobium gracile PCC 6307 /1-523
bchB GCA 000153825.1 ASM15382v1 protein EAU73303.1 light-independent protochlorophyllide reductase subunit B Synechococcus sp. RS9916 /1-509
bchB GCA 000153065.1 ASM15306v1 protein EAQ69763.1 Light-independent protochlorophyllide reductase B subunit Synechococcus sp. RS9917 /1-506
bchB GCA 000153805.1 ASM15380v1 protein EAU70497.1 light-independent protochlorophyllide reductase subunit B Synechococcus sp. BL107 /1-518
bchB GCA 000012505.1 ASM1250v1 protein ABB26578.1 Light-independent protochlorophyllide reductase B subunit Synechococcus sp. CC9902 /1-503
bchB GCA 000230675.2 ASM23067v1 protein EHA64172.1 Light-independent protochlorophyllide reductase subunit B Synechococcus sp. WH 8016 /1-500
bchB GCA 000014585.1 ASM1458v1 protein ABI46578.1 light-independent protochlorophyllide reductase B subunit Synechococcus sp. CC9311 /1-500
bchB GCA 001040845.1 ASM104084v1 protein AKN60853.1 light-independent protochlorophyllide reductase subunit B Synechococcus sp. WH 8020 /1-500
bchB d Bacteria p Cyanobacteria c Cyanobacteriia o Synechococcales f Cyanobiaceae g Synechococcus C s Synechococcus C sp001632165 WGS ID LVHU01 1/1-413
bchB GCA 000195975.1 ASM19597v1 protein CAE08239.1 light-independent protochlorophyllide reductase ChlB subunit Synechococcus sp. WH 8102 /1-502
bchB GCA 000161795.2 ASM16179v2 protein AHF64306.1 light-independent protochlorophyllide reductase B subunit Synechococcus sp. WH 8109 /1-502
bchB GCA 000012625.1 ASM1262v1 protein ABB34515.1 light-independent protochlorophyllide reductase subunit B Synechococcus sp. CC9605 /1-502
bchB GCA 000737595.1 ASM73759v1 protein AII50088.1 light-independent protochlorophyllide reductase subunit B Synechococcus sp. KORDI-52 /1-502
bchB GCA 000063505.1 ASM6350v1 protein CAK23097.1 Light-independent protochlorophyllide reductase subunit B Synechococcus sp. WH 7803 /1-504
bchB GCA 000153285.1 ASM15328v1 protein EAR20089.1 light-independent protochlorophyllide reductase subunit B Synechococcus sp. WH 7805 /1-502
bchB GCA 000737575.1 ASM73757v1 protein AII47273.1 light-independent protochlorophyllide reductase subunit B Synechococcus sp. KORDI-49 /1-501
bchB GCA 000737535.1 ASM73753v1 protein AII42812.1 light-independent protochlorophyllide reductase subunit B Synechococcus sp. KORDI-100 /1-499
bchB d Bacteria p Cyanobacteria c Cyanobacteriia o Synechococcales f Cyanobiaceae g Prochlorococcus C s Prochlorococcus C marinus B WGS ID LVHL01 1/1-412
bchB GCA 000760295.1 ASM76029v1 protein KGG25678.1 Light-independent protochlorophyllide reductase subunit B Prochlorococcus sp. MIT 0701 /1-506
bchB GCA 000760315.1 ASM76031v1 protein KGG29114.1 Light-independent protochlorophyllide reductase subunit B Prochlorococcus sp. MIT 0702 /1-508
bchB GCA 000015705.1 ASM1570v1 protein ABM77545.1 Light-independent protochlorophyllide reductase subunit B Prochlorococcus marinus str. MIT 9303 /1-508
bchB d Bacteria p Cyanobacteria c Cyanobacteriia o Synechococcales f Cyanobiaceae g AG-409-P01 s AG-409-P01 sp003215335 WGS ID QCKM01 1/1-413
bchB d Bacteria p Cyanobacteria c Cyanobacteriia o Synechococcales f Cyanobiaceae g AG-409-B05 s AG-409-B05 sp003216075 WGS ID QCJK01 1/1-413
bchB d Bacteria p Cyanobacteria c Cyanobacteriia o Synechococcales f Cyanobiaceae g AG-363-B04 s AG-363-B04 sp003278465 WGS ID QCFC01 1/1-413
bchB d Bacteria p Cyanobacteria c Cyanobacteriia o Synechococcales f Cyanobiaceae g AG-363-N20 s AG-363-N20 sp003280105 WGS ID QCFX01 1/1-411
bchB d Bacteria p Cyanobacteria c Cyanobacteriia o Synechococcales f Cyanobiaceae g AG-363-O15 s AG-363-O15 sp003278255 WGS ID QCFZ01 1/1-505
bchB d Bacteria p Cyanobacteria c Cyanobacteriia o Synechococcales f Cyanobiaceae g AG-409-G20 s AG-409-G20 sp003215775 WGS ID QCJW01 1/1-411
bchB d Bacteria p Cyanobacteria c Cyanobacteriia o Synechococcales f Cyanobiaceae g AG-363-O06 s AG-363-O06 sp003280085 WGS ID QCFY01 1/1-412
bchB d Bacteria p Cyanobacteria c Cyanobacteriia o Synechococcales f Cyanobiaceae g AG-409-M05 s AG-409-M05 sp003215435 WGS ID QCKJ01 1/1-412
bchB d Bacteria p Cyanobacteria c Cyanobacteriia o Synechococcales f Cyanobiaceae g AG-363-K07 s AG-363-K07 sp003280225 WGS ID QCFO01 1/1-413
bchB d Bacteria p Cyanobacteria c Cyanobacteriia o Synechococcales f Cyanobiaceae g AG-409-L18 s AG-409-L18 sp003215535 WGS ID QCKG01 1/1-413
bchB GCA 000760235.1 ASM76023v1 protein KGG19380.1 Light-independent protochlorophyllide reductase subunit B Prochlorococcus marinus str. PAC1 /1-503
bchB GCA 000757865.1 ASM75786v1 protein AIQ96733.1 Light-independent protochlorophyllide reductase subunit B Prochlorococcus sp. MIT 0801 /1-503
bchB GCA 000015685.1 ASM1568v1 protein ABM75161.1 Light-independent protochlorophyllide reductase subunit B Prochlorococcus marinus str. NATL1A /1-503
bchB GCA 000012465.1 ASM1246v1 protein AAZ59362.1 light-independent protochlorophyllide reductase B subunit Prochlorococcus marinus str. NATL2A /1-503
bchB GCA 000007925.1 ASM792v1 protein AAP99590.1 Light-independent protochlorophyllide reductase subunit B Prochlorococcus marinus subsp. marinus str. CCMP1375 /1-508
bchB GCA 000760195.1 ASM76019v1 protein KGG16415.1 Light-independent protochlorophyllide reductase subunit B Prochlorococcus sp. MIT 0602 /1-505
bchB GCA 000760215.1 ASM76021v1 protein KGG17111.1 Light-independent protochlorophyllide reductase subunit B Prochlorococcus sp. MIT 0603 /1-505
bchB d Bacteria p Cyanobacteria c Cyanobacteriia o Synechococcales f Cyanobiaceae g AG-402-N21 s AG-402-N21 sp003216435 WGS ID QCIW01 1/1-413
bchB GCA 000760175.1 ASM76017v1 protein KGG12847.1 Light-independent protochlorophyllide reductase subunit B Prochlorococcus sp. MIT 0601 /1-508
bchB GCA 000018585.1 ASM1858v1 protein ABX08477.1 Light-independent protochlorophyllide reductase subunit B Prochlorococcus marinus str. MIT 9211 /1-508
bchB d Bacteria p Cyanobacteria c Cyanobacteriia o Synechococcales f Cyanobiaceae g AG-363-P08 s AG-363-P08 sp003280055 WGS ID QCGG01 1/1-506
bchB d Bacteria p Cyanobacteria c Cyanobacteriia o Synechococcales f Cyanobiaceae g AG-409-J16 s AG-409-J16 sp003215655 WGS ID QCKB01 1/1-413
bchB d Bacteria p Cyanobacteria c Cyanobacteriia o Synechococcales f Cyanobiaceae g AG-363-A16 s AG-363-A16 sp003280345 WGS ID QCFB01 2/1-412
bchB GCA 000759855.1 ASM75985v1 protein KGF90988.1 Light-independent protochlorophyllide reductase subunit B Prochlorococcus marinus str. MIT 9107 /1-498
bchB GCA 000018065.1 ASM1806v1 protein ABV50240.1 Light-independent protochlorophyllide reductase subunit B Prochlorococcus marinus str. MIT 9215 /1-498
bchB GCA 000158595.1 ASM15859v1 protein EEE41112.1 light-independent protochlorophyllide reductase subunit B Prochlorococcus marinus str. MIT 9202 /1-498
bchB GCA 000759885.1 ASM75988v1 protein KGF88925.1 Light-independent protochlorophyllide reductase subunit B Prochlorococcus marinus str. GP2 /1-498
bchB GCA 000759955.1 ASM75995v1 protein KGF95314.1 Light-independent protochlorophyllide reductase subunit B Prochlorococcus marinus str. MIT 9201 /1-498
bchB GCA 000015665.1 ASM1566v1 protein ABM71817.1 Light-independent protochlorophyllide reductase subunit B Prochlorococcus marinus str. MIT 9515 /1-491
bchB GCA 000759875.1 ASM75987v1 protein KGF86106.1 Light-independent protochlorophyllide reductase subunit B Prochlorococcus marinus str. EQPAC1 /1-505
bchB GCA 000760015.1 ASM76001v1 protein KGF98682.1 Light-independent protochlorophyllide reductase subunit B Prochlorococcus marinus str. MIT 9311 /1-504
bchB GCA 000759975.1 ASM75997v1 protein KGF97488.1 Light-independent protochlorophyllide reductase subunit B Prochlorococcus marinus str. MIT 9302 /1-505
bchB GCA 000760115.1 ASM76011v1 protein KGG07985.1 Light-independent protochlorophyllide reductase subunit B Prochlorococcus marinus str. SB /1-498
bchB GCA 000015965.1 ASM1596v1 protein ABO17193.1 Light-independent protochlorophyllide reductase subunit B Prochlorococcus marinus str. MIT 9301 /1-498
bchB GCA 000760035.1 ASM76003v1 protein KGG03724.1 Light-independent protochlorophyllide reductase subunit B Prochlorococcus marinus str. MIT 9314 /1-498
bchB GCA 000015645.1 ASM1564v1 protein ABM69886.1 Light-independent protochlorophyllide reductase subunit B Prochlorococcus marinus str. AS9601 /1-498
bchB GCA 000760095.1 ASM76009v1 protein KGG07434.1 Light-independent protochlorophyllide reductase subunit B Prochlorococcus marinus str. MIT 9401 /1-498
bchB GCA 000757845.1 ASM75784v1 protein AIQ94613.1 Light-independent protochlorophyllide reductase subunit B Prochlorococcus sp. MIT 0604 /1-498
bchB d Bacteria p Proteobacteria c Alphaproteobacteria o Kiloniellales f Kiloniellaceae g BBD-1991-14 s BBD-1991-14 sp001657395 WGS ID LYSY01 1/1-411
bchB d Bacteria p Proteobacteria c Alphaproteobacteria o Rhodospirillales B f Magnetospirillaceae g Telmatospirillum s Telmatospirillum siberiense WGS ID PIUM01 1/1-493
bchB d Bacteria p Proteobacteria c Alphaproteobacteria o Acetobacterales f Acetobacteraceae g Paracraurococcus s Paracraurococcus sp003258945 WGS ID QLIX01 1/1-411
bchB d Bacteria p Myxococcota c Polyangia o Polyangiales f SG8-38 g GCA-2699025 s GCA-2699025 sp002699025 WGS ID PABA01 1/1-412
bchB d Bacteria p Myxococcota c Polyangia o Nannocystales f Nannocystaceae g Ga0077550 s Ga0077550 sp001464385 WGS ID LNFB01 1/1-413
bchB d Bacteria p Proteobacteria c Alphaproteobacteria o Rhizobiales f Phreatobacteraceae g Phreatobacter s Phreatobacter oligotrophus WGS ID PZZL01 1/1-502
bchB GCA 000636015.1 ASM63601v1 protein KAI94290.1 light-independent protochlorophyllide reductase subunit B Rhodomicrobium udaipurense JA643 /1-508
bchB GCA 000166055.1 ASM16605v1 protein ADP70271.1 light-independent protochlorophyllide reductase B subunit Rhodomicrobium vannielii ATCC 17100 /1-504
bchB GCA 000015165.1 ASM1516v1 protein ABQ38326.1 protochlorophyllide reductase BchB subunit Bradyrhizobium sp. BTAi1 /1-499
bchB GCA 000344805.1 ASM34480v1 protein BAM91875.1 light-independent protochlorophyllide reductase subunit B Bradyrhizobium oligotrophicum S58 /1-499
bchB GCA 000239775.2 ASM23977v2 protein CCD96367.1 protochlorophyllide reductase BchB subunit Bradyrhizobium sp. ORS 375 /1-499
bchB GCA 000026145.1 ASM2614v1 protein CAL75518.1 protochlorophyllide reductase BchB subunit Bradyrhizobium sp. ORS 278 /1-500
bchB GCA 000239755.2 ASM23975v2 protein CCD90189.1 protochlorophyllide reductase BchB subunit Bradyrhizobium sp. ORS 285 /1-500
bchB d Bacteria p Proteobacteria c Alphaproteobacteria o Rhizobiales f Anderseniellaceae g QKVK01 s QKVK01 sp003234965 WGS ID QKVK01 1/1-415
bchB d Bacteria p Proteobacteria c Alphaproteobacteria o Rhizobiales f Hyphomicrobiaceae g GCA-2689605 s GCA-2689605 sp002689605 WGS ID NZGY01 1/1-512
bchB d Bacteria p Proteobacteria c Alphaproteobacteria o Rhizobiales f Rhodomicrobiaceae g R-RK-3 s R-RK-3 sp002198715 WGS ID MWLJ01 1/1-498
bchB GCA 000154705.2 ASM15470v2 protein EDQ32697.1 light-independent protochlorophyllide reductase subunit B Hoeflea phototrophica DFL-43 /1-520
bchB d Bacteria p Proteobacteria c Alphaproteobacteria o Rhizobiales f Rhizobiaceae g 65-79 s 65-79 sp001896885 WGS ID MKRJ01 1/1-500
bchB GCA 000158095.1 ASM15809v1 protein EEE44581.1 light-independent protochlorophyllide reductase B subunit Labrenzia alexandrii DFL-11 /1-502
bchB d Bacteria p Proteobacteria c Alphaproteobacteria o Rhizobiales f Stappiaceae g Labrenzia s Labrenzia sp001890945 WGS ID MLAX01 1/1-503
bchB d Bacteria p Proteobacteria c Alphaproteobacteria o Rhizobiales f Rhizobiaceae g Allorhizobium s Allorhizobium sp001429245 WGS ID LMJE01 1/1-414
bchB GCA 001296005.1 ASM129600v1 protein KPF87283.1 light-independent protochlorophyllide reductase subunit B alpha proteobacterium AAP38 /1-498
bchB GCA 000299935.1 ASM29993v1 protein EKE76045.1 light-independent protochlorophyllide reductase subunit B Oceanibaculum indicum P24 /1-506
bchB GCA 001296045.1 ASM129604v1 protein KPF43467.1 light-independent protochlorophyllide reductase subunit B Rhizobium sp. AAP43 /1-504
bchB GCA 001295785.1 ASM129578v1 protein KPF61206.1 light-independent protochlorophyllide reductase subunit B Rhizobium sp. AAP116 /1-504
bchB GCA 000300855.1 ASM30085v1 protein EKF60967.1 light-independent protochlorophyllide reductase subunit B Agrobacterium albertimagni AOL15 /1-503
bchB d Bacteria p Proteobacteria c Alphaproteobacteria o Thalassobaculales f Thalassobaculaceae g Thalassobaculum s Thalassobaculum litoreum WGS ID AUIR01 1/1-412
bchB d Bacteria p Proteobacteria c Alphaproteobacteria o Rhodospirillales f Rhodospirillaceae g Rhodospira s Rhodospira trueperi WGS ID FNAP01 1/1-410

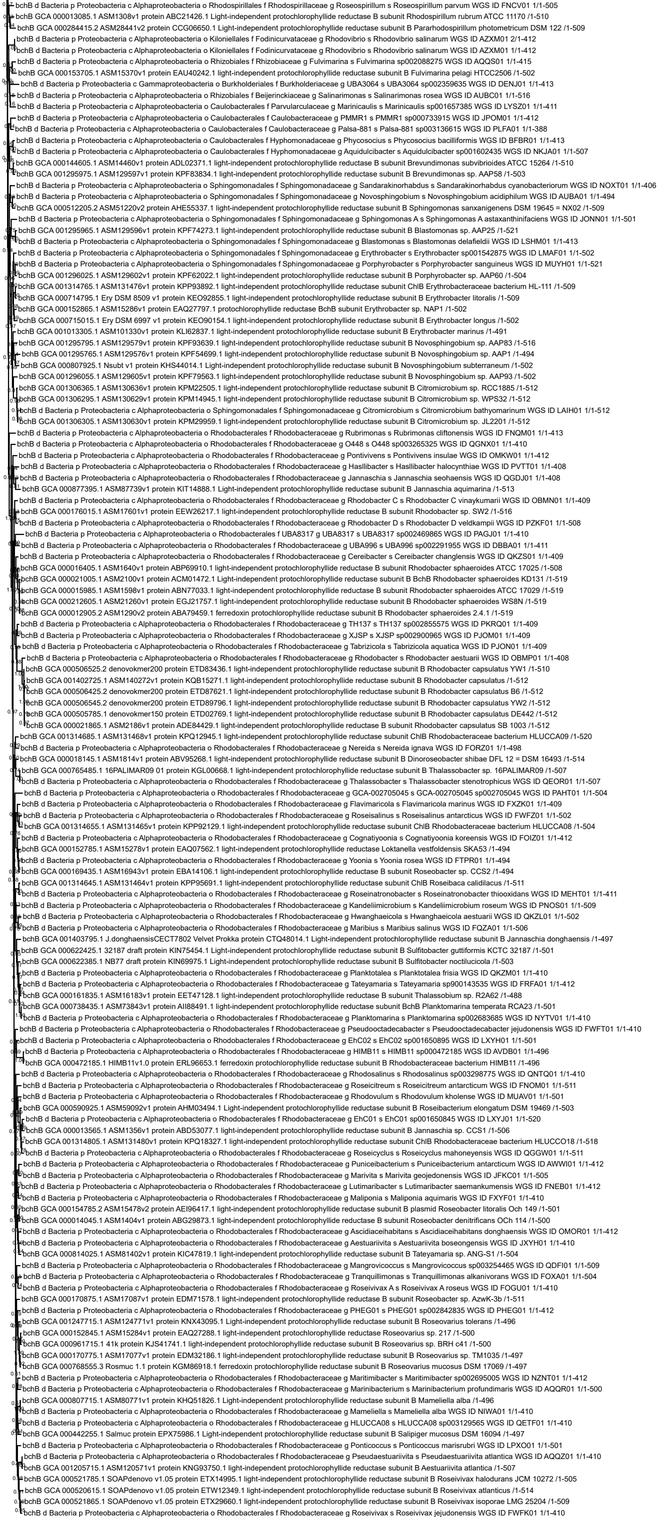
bchB d Bacteria p Proteobacteria c Alphaproteobacteria o Rhodospirillales f Rhodospirillaceae g Roseospirillum s Roseospirillum parvum WGS ID FNCV01 1/1-505
bchB GCA 000013085.1 ASM1308v1 protein ABC21426.1 Light-independent protochlorophyllide reductase B subunit Rhodospirillum rubrum ATCC 11170 /1-510
bchB GCA 000284415.2 ASM28441v2 protein CCG06650.1 Light-independent protochlorophyllide reductase subunit B Pararhodospirillum photometricum DSM 122 /1-509
bchB d Bacteria p Proteobacteria c Alphaproteobacteria o Kiloniellales f Fodinicurvataceae g Rhodovibrio s Rhodovibrio salinarum WGS ID AZXM01 2/1-412
bchB d Bacteria p Proteobacteria c Alphaproteobacteria o Kiloniellales f Fodinicurvataceae g Rhodovibrio s Rhodovibrio salinarum WGS ID AZXM01 1/1-412
bchB d Bacteria p Proteobacteria c Alphaproteobacteria o Rhizobiales f Rhizobiaceae g Fulvimarina s Fulvimarina sp002088275 WGS ID AQQS01 1/1-415
bchB GCA 000153705.1 ASM15370v1 protein EAU40242.1 light-independent protochlorophyllide reductase subunit B Fulvimarina pelagi HTCC2506 /1-502
bchB d Bacteria p Proteobacteria c Gammaproteobacteria o Burkholderiales f Burkholderiaceae g UBA3064 s UBA3064 sp002359635 WGS ID DENJ01 1/1-413
bchB d Bacteria p Proteobacteria c Alphaproteobacteria o Rhizobiales f Beijerinckiaceae g Salinarimonas s Salinarimonas rosea WGS ID AUBC01 1/1-516
bchB d Bacteria p Proteobacteria c Alphaproteobacteria o Caulobacterales f Parvularculaceae g Marinicaulis s Marinicaulis sp001657385 WGS ID LYSZ01 1/1-411
bchB d Bacteria p Proteobacteria c Alphaproteobacteria o Caulobacterales f Caulobacteraceae g PMMR1 s PMMR1 sp000733915 WGS ID JPOM01 1/1-412
bchB d Bacteria p Proteobacteria c Alphaproteobacteria o Caulobacterales f Caulobacteraceae g Palsa-881 s Palsa-881 sp003136615 WGS ID PLFA01 1/1-388
bchB d Bacteria p Proteobacteria c Alphaproteobacteria o Caulobacterales f Hyphomonadaceae g Phycosocius s Phycosocius bacilliformis WGS ID BFBR01 1/1-413
bchB d Bacteria p Proteobacteria c Alphaproteobacteria o Caulobacterales f Hyphomonadaceae g Aquidulcibacter s Aquidulcibacter sp001602435 WGS ID NKJA01 1/1-507
bchB GCA 000144605.1 ASM14460v1 protein ADL02371.1 light-independent protochlorophyllide reductase B subunit Brevundimonas subvibrioides ATCC 15264 /1-510
bchB GCA 001295975.1 ASM129597v1 protein KPF83834.1 light-independent protochlorophyllide reductase subunit B Brevundimonas sp. AAP58 /1-503
bchB d Bacteria p Proteobacteria c Alphaproteobacteria o Sphingomonadales f Sphingomonadaceae g Sandarakinorhabdus s Sandarakinorhabdus cyanobacteriorum WGS ID NOXT01 1/1-406
bchB d Bacteria p Proteobacteria c Alphaproteobacteria o Sphingomonadales f Sphingomonadaceae g Novosphingobium s Novosphingobium acidiphilum WGS ID AUBA01 1/1-494
bchB GCA 000512205.2 ASM51220v2 protein AHE55337.1 light-independent protochlorophyllide reductase subunit B Sphingomonas sanxanigenens DSM 19645 = NX02 /1-509
bchB d Bacteria p Proteobacteria c Alphaproteobacteria o Sphingomonadales f Sphingomonadaceae g Sphingomonas A s Sphingomonas A astaxanthinifaciens WGS ID JONN01 1/1-501
bchB GCA 001295965.1 ASM129596v1 protein KPF74273.1 light-independent protochlorophyllide reductase subunit B Blastomonas sp. AAP25 /1-521
bchB d Bacteria p Proteobacteria c Alphaproteobacteria o Sphingomonadales f Sphingomonadaceae g Blastomonas s Blastomonas delafieldii WGS ID LSHM01 1/1-413
bchB d Bacteria p Proteobacteria c Alphaproteobacteria o Sphingomonadales f Sphingomonadaceae g Erythrobacter s Erythrobacter sp001542875 WGS ID LMAF01 1/1-502
bchB d Bacteria p Proteobacteria c Alphaproteobacteria o Sphingomonadales f Sphingomonadaceae g Porphyrobacter s Porphyrobacter sanguineus WGS ID MUYH01 1/1-521
bchB GCA 001296025.1 ASM129602v1 protein KPF62022.1 light-independent protochlorophyllide reductase subunit B Porphyrobacter sp. AAP60 /1-504
bchB GCA 001314765.1 ASM131476v1 protein KPP93892.1 light-independent protochlorophyllide reductase subunit ChlB Erythrobacteraceae bacterium HL-111 /1-509
bchB GCA 000714795.1 Ery DSM 8509 v1 protein KEO92855.1 light-independent protochlorophyllide reductase subunit B Erythrobacter litoralis /1-509
bchB GCA 000152865.1 ASM15286v1 protein EAQ27797.1 protochlorophyllide reductase BchB subunit Erythrobacter sp. NAP1 /1-502
bchB GCA 000715015.1 Ery DSM 6997 v1 protein KEO90154.1 light-independent protochlorophyllide reductase subunit B Erythrobacter longus /1-502
bchB GCA 001013305.1 ASM101330v1 protein KLI62837.1 light-independent protochlorophyllide reductase subunit B Erythrobacter marinus /1-491
bchB GCA 001295795.1 ASM129579v1 protein KPF93639.1 light-independent protochlorophyllide reductase subunit B Novosphingobium sp. AAP83 /1-516
bchB GCA 001295765.1 ASM129576v1 protein KPF54699.1 light-independent protochlorophyllide reductase subunit B Novosphingobium sp. AAP1 /1-494
bchB GCA 000807925.1 Nsubt v1 protein KHS44014.1 Light-independent protochlorophyllide reductase subunit B Novosphingobium subterraneum /1-502
bchB GCA 001296055.1 ASM129605v1 protein KPF79563.1 light-independent protochlorophyllide reductase subunit B Novosphingobium sp. AAP93 /1-502
bchB GCA 001306365.1 ASM130636v1 protein KPM22505.1 light-independent protochlorophyllide reductase subunit B Citromicrobium sp. RCC1885 /1-512
bchB GCA 001306295.1 ASM130629v1 protein KPM14945.1 light-independent protochlorophyllide reductase subunit B Citromicrobium sp. WPS32 /1-512
bchB d Bacteria p Proteobacteria c Alphaproteobacteria o Sphingomonadales f Sphingomonadaceae g Citromicrobium s Citromicrobium bathyomarinum WGS ID LAIH01 1/1-512
bchB GCA 001306305.1 ASM130630v1 protein KPM29959.1 light-independent protochlorophyllide reductase subunit B Citromicrobium sp. JL2201 /1-512
bchB d Bacteria p Proteobacteria c Alphaproteobacteria o Rhodobacterales f Rhodobacteraceae g Rubrimonas s Rubrimonas cliftonensis WGS ID FNQM01 1/1-413
bchB d Bacteria p Proteobacteria c Alphaproteobacteria o Rhodobacterales f Rhodobacteraceae g O448 s O448 sp003265325 WGS ID QGNX01 1/1-410
bchB d Bacteria p Proteobacteria c Alphaproteobacteria o Rhodobacterales f Rhodobacteraceae g Pontivivens s Pontivivens insulae WGS ID OMKW01 1/1-412
bchB d Bacteria p Proteobacteria c Alphaproteobacteria o Rhodobacterales f Rhodobacteraceae g Hasllibacter s Hasllibacter halocynthiae WGS ID PVTT01 1/1-408
bchB d Bacteria p Proteobacteria c Alphaproteobacteria o Rhodobacterales f Rhodobacteraceae g Jannaschia s Jannaschia seohaensis WGS ID QGDJ01 1/1-408
bchB GCA 000877395.1 ASM87739v1 protein KIT14888.1 Light-independent protochlorophyllide reductase subunit B Jannaschia aquimarina /1-513
bchB d Bacteria p Proteobacteria c Alphaproteobacteria o Rhodobacterales f Rhodobacteraceae g Rhodobacter C s Rhodobacter C vinaykumarii WGS ID OBMN01 1/1-409
bchB GCA 000176015.1 ASM17601v1 protein EEW26217.1 light-independent protochlorophyllide reductase B subunit Rhodobacter sp. SW2 /1-516
bchB d Bacteria p Proteobacteria c Alphaproteobacteria o Rhodobacterales f Rhodobacteraceae g Rhodobacter D s Rhodobacter D veldkampii WGS ID PZKF01 1/1-508
bchB d Bacteria p Proteobacteria c Alphaproteobacteria o Rhodobacterales f UBA8317 g UBA8317 s UBA8317 sp002469865 WGS ID PAGJ01 1/1-410
bchB d Bacteria p Proteobacteria c Alphaproteobacteria o Rhodobacterales f Rhodobacteraceae g UBA996 s UBA996 sp002291955 WGS ID DBBA01 1/1-411
bchB d Bacteria p Proteobacteria c Alphaproteobacteria o Rhodobacterales f Rhodobacteraceae g Cereibacter s Cereibacter changlensis WGS ID QKZS01 1/1-409
bchB GCA 000016405.1 ASM1640v1 protein ABP69910.1 light-independent protochlorophyllide reductase B subunit Rhodobacter sphaeroides ATCC 17025 /1-508
bchB GCA 000021005.1 ASM2100v1 protein ACM01472.1 Light-independent protochlorophyllide reductase subunit B BchB Rhodobacter sphaeroides KD131 /1-519
bchB GCA 000015985.1 ASM1598v1 protein ABN77033.1 light-independent protochlorophyllide reductase B subunit Rhodobacter sphaeroides ATCC 17029 /1-519
bchB GCA 000212605.1 ASM21260v1 protein EGJ21757.1 light-independent protochlorophyllide reductase subunit B Rhodobacter sphaeroides WS8N /1-519
bchB GCA 000012905.2 ASM1290v2 protein ABA79459.1 ferredoxin protochlorophyllide reductase subunit B Rhodobacter sphaeroides 2.4.1 /1-519
bchB d Bacteria p Proteobacteria c Alphaproteobacteria o Rhodobacterales f Rhodobacteraceae g TH137 s TH137 sp002855575 WGS ID PKRQ01 1/1-409
bchB d Bacteria p Proteobacteria c Alphaproteobacteria o Rhodobacterales f Rhodobacteraceae g XJSP s XJSP sp002900965 WGS ID PJOM01 1/1-409
bchB d Bacteria p Proteobacteria c Alphaproteobacteria o Rhodobacterales f Rhodobacteraceae g Tabrizicola s Tabrizicola aquatica WGS ID PJON01 1/1-409
bchB d Bacteria p Proteobacteria c Alphaproteobacteria o Rhodobacterales f Rhodobacteraceae g Rhodobacter s Rhodobacter aestuarii WGS ID OBMP01 1/1-408
bchB GCA 000506525.2 denovokmer200 protein ETD83436.1 light-independent protochlorophyllide reductase subunit B Rhodobacter capsulatus YW1 /1-510
bchB GCA 001402725.1 ASM140272v1 protein KQB15271.1 light-independent protochlorophyllide reductase subunit B Rhodobacter capsulatus /1-512
bchB GCA 000506425.2 denovokmer200 protein ETD87621.1 light-independent protochlorophyllide reductase subunit B Rhodobacter capsulatus B6 /1-512
bchB GCA 000506545.2 denovokmer200 protein ETD89796.1 light-independent protochlorophyllide reductase subunit B Rhodobacter capsulatus YW2 /1-512
bchB GCA 000505785.1 denovokmer150 protein ETD02769.1 light-independent protochlorophyllide reductase subunit B Rhodobacter capsulatus DE442 /1-512
bchB GCA 000021865.1 ASM2186v1 protein ADE84429.1 light-independent protochlorophyllide reductase B subunit Rhodobacter capsulatus SB 1003 /1-512
bchB GCA 001314685.1 ASM131468v1 protein KPQ12945.1 light-independent protochlorophyllide reductase subunit ChlB Rhodobacteraceae bacterium HLUCCA09 /1-520
bchB d Bacteria p Proteobacteria c Alphaproteobacteria o Rhodobacterales f Rhodobacteraceae g Nereida s Nereida ignava WGS ID FORZ01 1/1-498
bchB GCA 000018145.1 ASM1814v1 protein ABV95268.1 light-independent protochlorophyllide reductase subunit B Dinoroseobacter shibae DFL 12 = DSM 16493 /1-514
bchB GCA 000765485.1 16PALIMAR09 01 protein KGL00668.1 light-independent protochlorophyllide reductase subunit B Thalassobacter sp. 16PALIMAR09 /1-507
bchB d Bacteria p Proteobacteria c Alphaproteobacteria o Rhodobacterales f Rhodobacteraceae g Thalassobacter s Thalassobacter stenotrophicus WGS ID QEOR01 1/1-507
bchB d Bacteria p Proteobacteria c Alphaproteobacteria o Rhodobacterales f Rhodobacteraceae g GCA-002705045 s GCA-002705045 sp002705045 WGS ID PAHT01 1/1-504
bchB d Bacteria p Proteobacteria c Alphaproteobacteria o Rhodobacterales f Rhodobacteraceae g Flavimaricola s Flavimaricola marinus WGS ID FXZK01 1/1-409
bchB d Bacteria p Proteobacteria c Alphaproteobacteria o Rhodobacterales f Rhodobacteraceae g Roseisalinus s Roseisalinus antarcticus WGS ID FWFZ01 1/1-502
bchB GCA 001314655.1 ASM131465v1 protein KPP92129.1 light-independent protochlorophyllide reductase subunit ChlB Rhodobacteraceae bacterium HLUCCA08 /1-504
bchB d Bacteria p Proteobacteria c Alphaproteobacteria o Rhodobacterales f Rhodobacteraceae g Cognatiyoonia s Cognatiyoonia koreensis WGS ID FOIZ01 1/1-412
bchB GCA 000152785.1 ASM15278v1 protein EAQ07562.1 light-independent protochlorophyllide reductase Loktanella vestfoldensis SKA53 /1-494
bchB d Bacteria p Proteobacteria c Alphaproteobacteria o Rhodobacterales f Rhodobacteraceae g Yoonia s Yoonia rosea WGS ID FTPR01 1/1-494
bchB GCA 000169435.1 ASM16943v1 protein EBA14106.1 light-independent protochlorophyllide reductase B subunit Roseobacter sp. CCS2 /1-494
bchB GCA 001314645.1 ASM131464v1 protein KPP95691.1 light-independent protochlorophyllide reductase subunit ChlB Roseibaca calidilacus /1-511
bchB d Bacteria p Proteobacteria c Alphaproteobacteria o Rhodobacterales f Rhodobacteraceae g Roseinatronobacter s Roseinatronobacter thiooxidans WGS ID MEHT01 1/1-411
bchB d Bacteria p Proteobacteria c Alphaproteobacteria o Rhodobacterales f Rhodobacteraceae g Kandeliimicrobium s Kandeliimicrobium roseum WGS ID PNOS01 1/1-509
bchB d Bacteria p Proteobacteria c Alphaproteobacteria o Rhodobacterales f Rhodobacteraceae g Hwanghaeicola s Hwanghaeicola aestuarii WGS ID QKZL01 1/1-502
bchB d Bacteria p Proteobacteria c Alphaproteobacteria o Rhodobacterales f Rhodobacteraceae g Maribius s Maribius salinus WGS ID FQZA01 1/1-506
bchB GCA 001403795.1 J.donghaensisCECT7802 Velvet Prokka protein CTQ48014.1 Light-independent protochlorophyllide reductase subunit B Jannaschia donghaensis /1-497
bchB GCA 000622425.1 32187 draft protein KIN75454.1 Light-independent protochlorophyllide reductase subunit B Sulfitobacter guttiformis KCTC 32187 /1-501
bchB GCA 000622385.1 NB77 draft protein KIN69975.1 Light-independent protochlorophyllide reductase subunit B Sulfitobacter noctilucicola /1-503
bchB d Bacteria p Proteobacteria c Alphaproteobacteria o Rhodobacterales f Rhodobacteraceae g Planktotalea s Planktotalea frisia WGS ID QKZM01 1/1-410
bchB d Bacteria p Proteobacteria c Alphaproteobacteria o Rhodobacterales f Rhodobacteraceae g Tateyamaria s Tateyamaria sp900143535 WGS ID FRFA01 1/1-412
bchB GCA 000161835.1 ASM16183v1 protein EET47128.1 light-independent protochlorophyllide reductase B subunit Thalassobium sp. R2A62 /1-488
bchB GCA 000738435.1 ASM73843v1 protein AII88491.1 light-independent protochlorophyllide reductase subunit BchB Planktomarina temperata RCA23 /1-501
bchB d Bacteria p Proteobacteria c Alphaproteobacteria o Rhodobacterales f Rhodobacteraceae g Planktomarina s Planktomarina sp002683685 WGS ID NYTV01 1/1-410
bchB d Bacteria p Proteobacteria c Alphaproteobacteria o Rhodobacterales f Rhodobacteraceae g Pseudooctadecabacter s Pseudooctadecabacter jejudonensis WGS ID FWFT01 1/1-410
bchB d Bacteria p Proteobacteria c Alphaproteobacteria o Rhodobacterales f Rhodobacteraceae g EhC02 s EhC02 sp001650895 WGS ID LXYH01 1/1-501
bchB d Bacteria p Proteobacteria c Alphaproteobacteria o Rhodobacterales f Rhodobacteraceae g HIMB11 s HIMB11 sp000472185 WGS ID AVDB01 1/1-496
bchB GCA 000472185.1 HIMB11v1.0 protein ERL96653.1 ferredoxin protochlorophyllide reductase subunit B Rhodobacteraceae bacterium HIMB11 /1-496
bchB d Bacteria p Proteobacteria c Alphaproteobacteria o Rhodobacterales f Rhodobacteraceae g Rhodosalinus s Rhodosalinus sp003298775 WGS ID QNTQ01 1/1-410
bchB d Bacteria p Proteobacteria c Alphaproteobacteria o Rhodobacterales f Rhodobacteraceae g Roseicitreum s Roseicitreum antarcticum WGS ID FNOM01 1/1-511
bchB d Bacteria p Proteobacteria c Alphaproteobacteria o Rhodobacterales f Rhodobacteraceae g Rhodovulum s Rhodovulum kholense WGS ID MUAV01 1/1-501
bchB GCA 000590925.1 ASM59092v1 protein AHM03494.1 Light-independent protochlorophyllide reductase subunit B Roseibacterium elongatum DSM 19469 /1-503
bchB d Bacteria p Proteobacteria c Alphaproteobacteria o Rhodobacterales f Rhodobacteraceae g EhC01 s EhC01 sp001650845 WGS ID LXYJ01 1/1-520
bchB GCA 000013565.1 ASM1356v1 protein ABD53077.1 light-independent protochlorophyllide reductase subunit B Jannaschia sp. CCS1 /1-506
bchB GCA 001314805.1 ASM131480v1 protein KPQ18327.1 light-independent protochlorophyllide reductase subunit ChlB Rhodobacteraceae bacterium HLUCCO18 /1-518
bchB d Bacteria p Proteobacteria c Alphaproteobacteria o Rhodobacterales f Rhodobacteraceae g Roseicyclus s Roseicyclus mahoneyensis WGS ID QGGW01 1/1-511
bchB d Bacteria p Proteobacteria c Alphaproteobacteria o Rhodobacterales f Rhodobacteraceae g Puniceibacterium s Puniceibacterium antarcticum WGS ID AWWI01 1/1-412
bchB d Bacteria p Proteobacteria c Alphaproteobacteria o Rhodobacterales f Rhodobacteraceae g Marivita s Marivita geojedonensis WGS ID JFKC01 1/1-505
bchB d Bacteria p Proteobacteria c Alphaproteobacteria o Rhodobacterales f Rhodobacteraceae g Lutimaribacter s Lutimaribacter saemankumensis WGS ID FNEB01 1/1-412
bchB d Bacteria p Proteobacteria c Alphaproteobacteria o Rhodobacterales f Rhodobacteraceae g Maliponia s Maliponia aquimaris WGS ID FXYF01 1/1-410
bchB GCA 000154785.2 ASM15478v2 protein AEI96417.1 light-independent protochlorophyllide reductase subunit B plasmid Roseobacter litoralis Och 149 /1-501
bchB GCA 000014045.1 ASM1404v1 protein ABG29873.1 light-independent protochlorophyllide reductase subunit B Roseobacter denitrificans OCh 114 /1-500
bchB d Bacteria p Proteobacteria c Alphaproteobacteria o Rhodobacterales f Rhodobacteraceae g Ascidiaceihabitans s Ascidiaceihabitans donghaensis WGS ID OMOR01 1/1-412
bchB d Bacteria p Proteobacteria c Alphaproteobacteria o Rhodobacterales f Rhodobacteraceae g Aestuariivita s Aestuariivita boseongensis WGS ID JXYH01 1/1-410
bchB GCA 000814025.1 ASM81402v1 protein KIC47819.1 light-independent protochlorophyllide reductase subunit B Tateyamaria sp. ANG-S1 /1-504
bchB d Bacteria p Proteobacteria c Alphaproteobacteria o Rhodobacterales f Rhodobacteraceae g Mangrovicoccus s Mangrovicoccus sp003254465 WGS ID QDFI01 1/1-509
bchB d Bacteria p Proteobacteria c Alphaproteobacteria o Rhodobacterales f Rhodobacteraceae g Tranquillimonas s Tranquillimonas alkanivorans WGS ID FOXA01 1/1-504
bchB d Bacteria p Proteobacteria c Alphaproteobacteria o Rhodobacterales f Rhodobacteraceae g Roseivivax A s Roseivivax A roseus WGS ID FOGU01 1/1-410
bchB GCA 000170875.1 ASM17087v1 protein EDM71578.1 light-independent protochlorophyllide reductase B subunit Roseobacter sp. AzwK-3b /1-511
bchB d Bacteria p Proteobacteria c Alphaproteobacteria o Rhodobacterales f Rhodobacteraceae g PHEG01 s PHEG01 sp002842835 WGS ID PHEG01 1/1-412
bchB GCA 001247715.1 ASM124771v1 protein KNX43095.1 Light-independent protochlorophyllide reductase subunit B Roseovarius tolerans /1-496
bchB GCA 000152845.1 ASM15284v1 protein EAQ27288.1 light-independent protochlorophyllide reductase Roseovarius sp. 217 /1-500
bchB GCA 000961715.1 41k protein KJS41741.1 light-independent protochlorophyllide reductase subunit B Roseovarius sp. BRH c41 /1-500
bchB GCA 000170775.1 ASM17077v1 protein EDM32186.1 light-independent protochlorophyllide reductase subunit B Roseovarius sp. TM1035 /1-497
bchB GCA 000768555.3 Rosmuc 1.1 protein KGM86918.1 ferredoxin protochlorophyllide reductase subunit B Roseovarius mucosus DSM 17069 /1-497
bchB d Bacteria p Proteobacteria c Alphaproteobacteria o Rhodobacterales f Rhodobacteraceae g Maritimibacter s Maritimibacter sp002695005 WGS ID NZNT01 1/1-412
bchB d Bacteria p Proteobacteria c Alphaproteobacteria o Rhodobacterales f Rhodobacteraceae g Marinibacterium s Marinibacterium profundimaris WGS ID AQQR01 1/1-500
bchB GCA 000807715.1 ASM80771v1 protein KHQ51826.1 Light-independent protochlorophyllide reductase subunit B Mameliella alba /1-496
bchB d Bacteria p Proteobacteria c Alphaproteobacteria o Rhodobacterales f Rhodobacteraceae g Mameliella s Mameliella alba WGS ID NIWA01 1/1-410
bchB d Bacteria p Proteobacteria c Alphaproteobacteria o Rhodobacterales f Rhodobacteraceae g HLUCCA08 s HLUCCA08 sp003129565 WGS ID QETF01 1/1-410
bchB GCA 000442255.1 Salmuc protein EPX75986.1 Light-independent protochlorophyllide reductase subunit B Salipiger mucosus DSM 16094 /1-497
bchB d Bacteria p Proteobacteria c Alphaproteobacteria o Rhodobacterales f Rhodobacteraceae g Ponticoccus s Ponticoccus marisrubri WGS ID LPXO01 1/1-501
bchB d Bacteria p Proteobacteria c Alphaproteobacteria o Rhodobacterales f Rhodobacteraceae g Pseudaestuariivita s Pseudaestuariivita atlantica WGS ID AQQZ01 1/1-410
bchB GCA 001205715.1 ASM120571v1 protein KNG93750.1 light-independent protochlorophyllide reductase subunit B Aestuariivita atlantica /1-507
bchB GCA 000521785.1 SOAPdenovo v1.05 protein ETX14995.1 light-independent protochlorophyllide reductase subunit B Roseivivax halodurans JCM 10272 /1-505
bchB GCA 000520615.1 SOAPdenovo v1.05 protein ETW12349.1 light-independent protochlorophyllide reductase subunit B Roseivivax atlanticus /1-514
bchB GCA 000521865.1 SOAPdenovo v1.05 protein ETX29660.1 light-independent protochlorophyllide reductase subunit B Roseivivax isoporae LMG 25204 /1-509
bchB d Bacteria p Proteobacteria c Alphaproteobacteria o Rhodobacterales f Rhodobacteraceae g Roseivivax s Roseivivax jejudonensis WGS ID FWFK01 1/1-410
