# Supplementary material for: Granick revisited: Synthesizing evolutionary and ecological evidence for the late origin of bacteriochlorophyll via ghost lineages and horizontal gene transfer
Source: PLoS One. 2021 Jan 28;16(1):e0239248. doi: 10.1371/journal.pone.0239248 (PMC7842958; doi:10.1371/journal.pone.0239248)

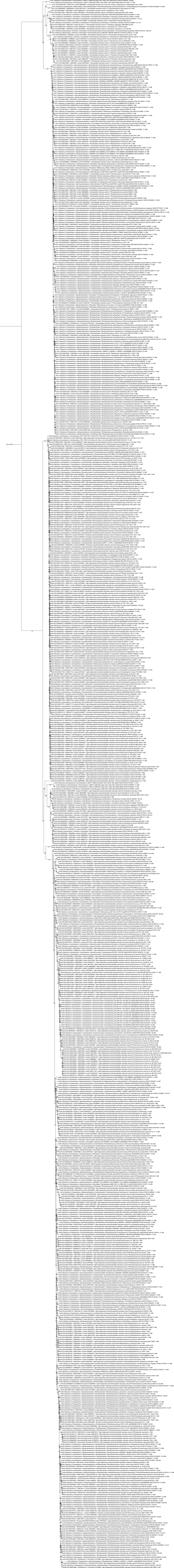

Supplement: S16 Fig — (PDF) [file pone.0239248.s016.pdf]
